# Synthesis and Characterization of Cerium-Oxo Clusters Capped by Acetylacetonate

**DOI:** 10.1021/acs.inorgchem.3c02141

**Published:** 2023-10-04

**Authors:** Anamar Blanes-Díaz, Mohammad Shohel, Natalie T. Rice, Ida Piedmonte, Morgan A. McDonald, Kaveh Jorabchi, Stosh A. Kozimor, Jeffery A. Bertke, May Nyman, Karah E. Knope

**Affiliations:** † Department of Chemistry, Georgetown University, 37th and O Streets NW, Washington, D.C. 20057, United States; ‡ Department of Chemistry, Oregon State University, Corvallis, Oregon 97331, United States; § Los Alamos National Laboratory (LANL), P.O. Box 1663, Los Alamos, New Mexico 87545, United States

## Abstract

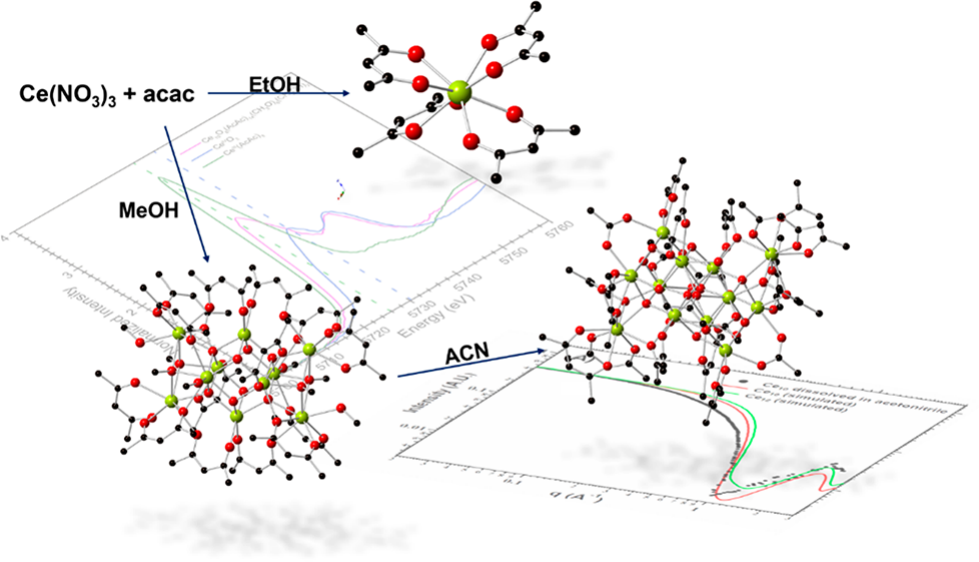

Cerium-oxo clusters have applications in fields ranging from catalysis to electronics and also hold the potential to inform on aspects of actinide chemistry. Toward this end, a cerium-acetylacetonate (acac^1–^) monomeric molecule, Ce(acac)_4_ (**Ce-1**), and two acac^1–^-decorated cerium-oxo clusters, [Ce_10_O_8_(acac)_14_(CH_3_O)_6_(CH_3_OH)_2_]·10.5MeOH (**Ce-10**) and [Ce_12_O_12_(OH)_4_(acac)_16_(CH_3_COO)_2_]·6(CH_3_CN) (**Ce-12**), were prepared and structurally characterized. The Ce(acac)_4_ monomer contains Ce^IV^. Crystallographic data and bond valence summation values for the **Ce-10** and **Ce-12** clusters are consistent with both clusters having a mixture of Ce^III^ and Ce^IV^ cations. Ce L_3_-edge X-ray absorption spectroscopy, performed on **Ce-10**, showed contributions from both Ce^III^ and Ce^IV^. The **Ce-10** cluster is built from a hexameric cluster, with six Ce^IV^ sites, that is capped by two dimeric Ce^III^ units. By comparison, **Ce-12**, which formed upon dissolution of **Ce-10** in acetonitrile, consists of a central decamer built from edge sharing Ce^IV^ hexameric units, and two monomeric Ce^III^ sites that are bound on the outer corners of the inner Ce_10_ core. Electrospray ionization mass spectrometry data for solutions prepared by dissolving **Ce-10** in acetonitrile showed that the major ions could be attributed to Ce_10_ clusters that differed primarily in the number of acac^1–^, OH^1–^, MeO^1–^, and O^2–^ ligands. Small angle X-ray scattering measurements for **Ce-10** dissolved in acetonitrile showed structural units slightly larger than either Ce_10_ or Ce_12_ in solution, likely due to aggregation. Taken together, these results suggest that the acetylacetonate supported clusters can support diverse solution-phase speciation in organic solutions that could lead to stabilization of higher order cerium containing clusters, such as cluster sizes that are greater than the Ce_10_ and Ce_12_ reported herein.

## Abstract 2

The synthesis and solid-state structural chemistry of a cerium^IV^-acetylacetonate monomeric unit, and two unique cerium^III/IV^-oxo clusters, Ce_10_ and Ce_12_, are described. The solution stability of Ce_10_ was examined using electrospray ionization mass spectrometry, small-angle X-ray scattering, and NMR spectroscopy. While the cluster readily forms from methanolic solutions, dissolution in acetonitrile likely results in aggregation into average larger species in solution, and, ultimately, precipitation of Ce_12_.

## Introduction

Cerium-based materials have found application in areas ranging from catalysis to downconverters in light emitting diodes.^1−7^ These applications, together with the relative abundance and low cost of cerium, have motivated efforts focused on structure–property relationships and hence materials development. Among cerium compounds, CeO_2_ (ceria) stands out as having uniquely diverse properties. One of the most notable industrial applications of ceria is its use in three-way catalysts (TWCs); ceria can serve several purposes for TWCs, including acting as an oxygen storage component due to its reduction potential.^8,9^ In fact, the accessibility of the +3 and +4 oxidation states prompts many efforts that seek to harness the redox chemistry of cerium toward uses in optics, solid-oxide fuels, and oxygen storage.^10−12^ Largely motivated to (1) better understand the catalytic behavior of CeO_2_ and (2) develop novel Ce-based catalysts that can be fine-tuned for specific applications, the chemistry community has become interested in cerium-oxo clusters. These species can be regarded as molecular scale nanoparticles of CeO_2_, and their properties seem to depend on the nuclearity and composition of the Ce cluster.^13^ As a testament, Christou et al. recently demonstrated the ability of Ce-oxo clusters to scavenge reactive oxygen species, and the activity of these clusters was dependent on the number of Ce^III^ atoms present in the cluster.^14^ It seems likely that the catalysis applications space for Ce-oxo clusters is wide and diverse because there are a large number of Ce-oxo clusters that can be prepared. For example, synthetic chemists have discovered reproducible synthetic methods for molecules that have between two cerium atoms (homometallic dimers; Ce_2_) and clusters with up to one hundred cerium atoms (Ce_100_). In an attempt to further diversify the landscape of Ce-oxo cluster chemistry, new avenues in Ce-oxo cluster synthesis are currently being explored by many research teams.^6,14−25^

In addition to serving as a platform for realizing novel materials properties, Ce-oxo clusters may also provide important insight into the chemical behavior of the lower valent actinides.^26,27^ Cerium has historically served as a surrogate for plutonium, owing to similarities in ionic radii and accessible +3 and +4 oxidation states.^28−32^ Specifically regarding cluster chemistry, there are several phases, including the M_6_-, M_22_-, and M_38_-oxo clusters, that have been isolated for both Ce and Pu ions.^20,24,25,33−35^ Similarities between Ce and Pu suggest that Ce may serve as a potential guide for expanding Pu cluster chemistry. The latter is significant because nanosized Pu-oxo cluster chemistry is of potential relevancy for plutonium processing chemistry and fate and transport in the environment.^36−39^ In this regard, defining Ce-oxo cluster chemistry will advance our overall understanding of lanthanide vs actinide cluster chemistry, thereby improving our ability to control the chemistry of f-element oxo-clusters and advance our predictive capabilities in this area. Indeed, the potential translatability of Ce to actinide cluster chemistry motivates our current efforts.

Previously, our group reported the synthesis and characterization of a Ce_38_-oxo cluster that was isolated from halide media using K^1+^ counterions.^24^ The compound was prepared through evaporation and exhibited a solid-state phase transformation that underscored the surface lability and reactivity of chloride ligated Ce-oxo clusters. Herein, we sought to limit synthetic challenges by controlling the surface lability and Ce redox chemistry. We achieved this control by leveraging nonaqueous alcoholic solutions and through the use of acetylacetonate (acac^1–^) as a bidentate cluster capping agent. Note, acac^1–^ has been used previously in lanthanide cluster chemistry.^40,41^ Toward this end, we examined reactions between cerium and acac^1–^ in alcoholic solvents: methanol and ethanol. We then developed synthetic methodology for monomeric Ce(acac)_4_ as well as for two novel acac^1–^ capped clusters: [Ce_10_O_8_(acac)_14_(CH_3_O)_6_(CH_3_OH)_2_]·10.5MeOH (**Ce-10**) and [Ce_12_O_12_(OH)_4_(acac)_16_(CH_3_COO)_2_]·6(CH_3_CN) (**Ce-12**). The structures were characterized by single-crystal X-ray diffraction. Bond valence summation values for the Ce sites^42,43^ suggested that Ce(acac)_4_ contained Ce^IV^, **Ce-10** contained both Ce^III^ and Ce^IV^, and **Ce-12** also contained Ce^III^ and Ce^IV^. Examination of **Ce-10** using X-ray absorption spectroscopy (XAS) was consistent with the mixed oxidation state Ce^III^/Ce^IV^ formulation. The vibrational properties of the compounds are also described. The stability of the **Ce-10** cluster in solution was examined via ^1^H NMR spectroscopy, small-angle X-ray scattering (SAXS), and electrospray ionization mass spectrometry; these data suggest that upon dissolution in acetonitrile, **Ce-10** aggregates into particles with a larger average size than the **Ce-10** and **Ce-12** clusters. Overall, this work points to the utility of β-diketonate ligands in the isolation of Ce-oxo clusters and the importance of solvent on cluster topology. The latter is evidenced by the precipitation of monomers from ethanol, decamers from methanol, and dodecamers from acetonitrile. This observation highlights the unique solvent role in Ce-oxo formation, and we are excited at the prospect of better defining solvent effects in the following studies. Moreover, given the simplicity and facile reproducibility of the synthesis, this organic system may be an entry to differentiating cluster chemistry for plutonium and, potentially, other actinide elements.

## Experimental Section

### Materials

The following chemicals were purchased from commercial suppliers and used as received: Ce(NO_3_)_3_·6H_2_O (ACROS Organics), acetylacetone (Hacac; TCI America), methanol (Fisher Chemical), 200 proof ethanol (The Warner-Graham Company), triethylamine (Sigma-Aldrich), acetonitrile (MeCN; Fisher Chemical), and hexanes (Fisher Chemical). All of the following reactions were performed under ambient conditions.

### Synthetic Details

Compound **Ce-1**, Ce(acac)_4_, was obtained by dissolving Ce(NO_3_)_3_·6H_2_O (0.3262 g, 1 mmol) and Hacac (204 μL, 2 mmol) in EtOH (3 mL) in a glass vial (7 mL). The solution was sonicated for five min, and then triethylamine (557 μL, 4 mmol) was added dropwise to the solution. The solution was then cooled to 15 °C. After 1 week, orange, rod-like crystals of **Ce-1** and an unidentified yellow–orange powder had precipitated. The mother liquor was removed from the vial, and the reaction product was washed (3 × 3 mL) with a one-to-one mixture of EtOH-to-hexanes. The crystals were left to dry under ambient conditions (1 h). Note that based on visual inspection and PXRD (Figure S13), **Ce-1** was a minor phase in the reaction product.

Compound **Ce-10**, [Ce_10_O_8_(acac)_14_(CH_3_O)_6_(CH_3_OH)_2_]·10.5MeOH, was prepared using various synthetic conditions, as detailed in the Supporting Information. The synthesis described here is readily reproducible and yields a pure phase. Ce(NO_3_)_3_·6H_2_O (0.3262 g, 1 mmol) and Hacac (204 μL, 2 mmol) were added to a glass vial (7 mL) that contained MeOH (3 mL). The solution was sonicated (5 min) until the Ce(NO_3_)_3_·6H_2_O dissolved. Once dissolved, triethylamine (557 μL, 4 mmol) was added dropwise to yield a dark-orange solution. The solution was then capped and cooled to 15 °C. After 24 h, orange crystals formed, and the mother liquor was removed. The crystals were washed (3 × 3 mL) with a one-to-one mixture of MeOH-to-hexanes. Then the crystals were left to dry under ambient conditions (1 h). Yield based on Ce: 20%. Elemental analysis calc (obs) C: 29.60 (29.81), H: 3.89 (3.88).

Compound **Ce-12**, [Ce_12_O_12_(OH)_4_(acac)_16_(CH_3_COO)_2_]·6(MeCN), was obtained by combining [Ce_10_O_8_(acac)_14_(CH_3_O)_4_(CH_3_OH)_4_]·10.5MeOH (**Ce-10**, 10 mg, 0.003 mmol) and acetonitrile (0.5 mL) in a glass vial (7 mL). A yellowish–brown suspension formed after the mixture was sonicated (10 min). The vial was capped, and after 1 week, yellow single crystals (rod shaped) of **Ce-12** formed alongside an unidentified light brown powder. Note that the compound is formulated with two acetate ligands that are presumed to form *in situ*, vide infra.

#### Structure Determination by Single Crystal X-ray Diffraction

Crystals of **Ce-1**, **Ce-10**, and **Ce-12** were isolated from the bulk reaction products and mounted on MiTeGen loops in Paratone oil. Single crystal X-ray diffraction data were collected at 100 K on a Bruker D8 Quest diffractometer equipped with a Mo Kα microfocus source (λ = 0.71073 Å), an Oxford 700 Cryostream, and a Photon100 detector. The data were integrated using the SAINT software package included in APEX3, and absorption corrections were applied using a multiscan technique in SADABS. The structures were solved using SHELXT and refined by full matrix least-squares on F2 using SHELXL software on shelXle64.^44−48^ Crystal data and structure refinement details are provided in Table 1; further details of the structure refinements are provided in the Supporting Information.

**Table 1:** Crystallographic Structure Refinement Details for **Ce-1**, **Ce-10**, and **Ce-12**

	**Ce-1**	**Ce-10**	**Ce-12**
Formula	C_30_H_28_CeO_8_	C_78_ H_122_ Ce_10_ O_44_	[C_84_H_118_Ce_12_O_52_](C_2_H_3_N)_6_
MW (g mol^–1^)	536.54	3164.95	3887.54
*T* (K)	100	100	100
crystal color/habit	orange rod	orange block	yellow block
crystal system	monoclinic	monoclinic	triclinic
λ (Å)	0.71073	0.71073	0.71073
Space group	*C*2/*c*	P2_1_/n	P-1
*a* (Å)	21.5200(13)	14.7545(8)	14.173(5)
*b* (Å)	8.3715(5)	20.6763(11)	16.254(6)
*c* (Å)	13.9042(9)	20.1617(11)	16.770(9)
α (deg)	90	90	112.390(14)
β (deg)	114.353(2)	108.647(2)	108.333(18)
γ (deg)	90	90	98.191(13)
*V* (Å^3^)	2282.0(2)	5827.8(5)	3236(3)
Z	2	2	2
ρ (mg m^–3^)	1.562	1.804	1.995
μ (mm^–1^)	2.035	3.892	4.209
*R*_1_	0.0184	0.0484	0.0448
w*R*_2_	0.095	0.1017	0.1477
GOF	1.414	1.028	1.035
CCDC	2257004	2257005	2257002

#### Bulk Solid-State Characterization Methods

Powder X-ray diffraction data for the bulk samples from which crystals of **Ce-1**, **Ce-10**, and **Ce-12** were isolated were obtained using Cu–Kα radiation (λ = 1.542 Å) on a Rigaku Ultima IV X-ray diffractometer from 3 to 40° in 2θ with a step speed of 1 degree/min (Figures S13–S15). IR spectra for **Ce-10** and **Ce-12** were collected on single crystals using a Nicolet iN10 Infrared Microscope FTIR-ATR with a Ge ATR tip over Δν 675–4000 cm^–1^ (Figures S17–S18). For **Ce-10**, combustion elemental analysis was performed on the bulk sample by using a PerkinElmer model 2400 elemental analyzer.

### X-ray Absorption Spectroscopy (XAS)

A sample of **Ce-10**, and oxidation state standards, CeO_2_ and Ce(acac)_3_·(H_2_O)_*x*_, were prepared as detailed in the Supporting Information. Ce L_3_-edge XAS data were collected in transmission mode using an in-house XAS spectrometer at Los Alamos National Laboratory.^49^ Fifty scans were obtained and averaged per cerium sample, and four scans were obtained and averaged for the Cr foil (used for energy calibration). Data manipulation and analysis were conducted as previously described by Solomon and co-workers.^50^ Further details on the instrument configuration, data collection, and data analysis are available in the Supporting Information.

### Solution State Characterization Methods

#### Small Angle X-ray Scattering (SAXS)

X-ray scattering data were collected on an Anton Paar SAXS instrument using Cu–Kα radiation (1.54 Å) equipped with line collimation. A 2-D image plate was used for data collection in the *q* = 0.018–2.5 Å^–1^ range. The lower *q* resolution is limited by the beam attenuator. The solution obtained by dissolving **Ce-10** in acetonitrile was filtered with a 0.45 μm membrane filter and then filled in a 1.5 mm glass capillary (Hampton Research) for the SAXS measurement. Scattering data of neat solvent were also collected for background subtraction. Scattering was measured for 30 min for each experiment. SAXSQUANT software was used for data collection and post processing (normalization, primary beam removal, background subtraction, desmearing, and smoothing to remove extra noise created by the desmearing routine). Data were analyzed using IRENA macros with IgorPro 6.3 (Wavemetrics) software.^51^ Simulated scattering patterns of the **Ce-10** and **Ce-12** clusters were generated using SolX utilizing structural files (.xyz) containing a selected portion of the structure that did not include solvent or coordinated ligands.^52^

#### ^1^H Nuclear Magnetic Resonance Spectroscopy

Crystals of **Ce-10** were dissolved in deuterated acetonitrile (CD_3_CN) and filtered through Celite. A ^1^H NMR spectrum was then collected using a Varian 400-MR NMR. The spectrum (Figure S22) and peak assignments (Table S6) are provided as Supporting Information.

#### Electrospray Ionization Mass Spectrometry

Mass spectra were collected by using nanospray ionization and a quadrupole time-of-flight instrument (QStar XL, Sciex, Ontario, Canada). Crystals of **Ce-10** (4.5 mg) were dissolved in acetonitrile, and the solution was diluted to approximately 50 μM. The sample solution was then loaded into a pulled borosilicate capillary (1 mm o.d. 0.75 mm i.d. pulled to 5 μm tip size) with its tip placed ∼1 cm from the curtain plate inlet of the mass spectrometer. A platinum electrode was inserted into the back of the capillary to make electrical contact with the solution for nanospray generation. Voltages of 2200–2400 V were applied to the solution while the spectrometer plate was held at 1100 V. The instrument was operated in TOF mode without any collision-induced dissociation gas in Q2 using DP1 = 50 V and DP2 = 10 V. These conditions produced a gentle ion sampling while helping desolvate the noncovalently bound solvent molecules.

## Results and Discussion

### Structure Descriptions

**Figure 1:**
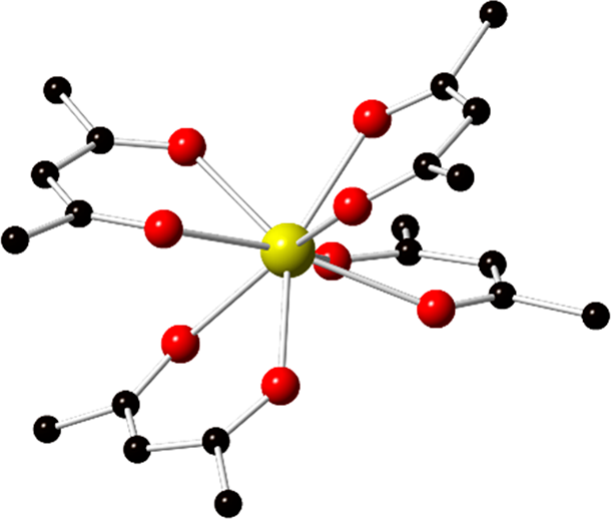
Ball and stick illustration of the Ce(acac)_4_ monomer. Ce is shown in yellow, O in red, and C in black. Hydrogen atoms have been removed for clarity.

Compound **Ce-1**, Ce(acac)_4_, crystallized in the monoclinic space group, *C2/c.* The structure consists of a Ce^IV^ monomeric unit, Ce(acac)_4_, that is composed of an eight-coordinate cerium(IV) metal center bound to oxygen atoms from four acac^1–^ ligands as shown in Figure 1. The acac^1–^ ligands bind in a bidentate fashion, with Ce–O bond distances ranging from 2.311(2)–2.348(2) Å. Note that **Ce-1** is isostructural with a Ce(acac)_4_ monomer reported by Matkovic and Grdenic;^53^ however, the compound crystallizes with a different unit cell.

**Figure 2:**
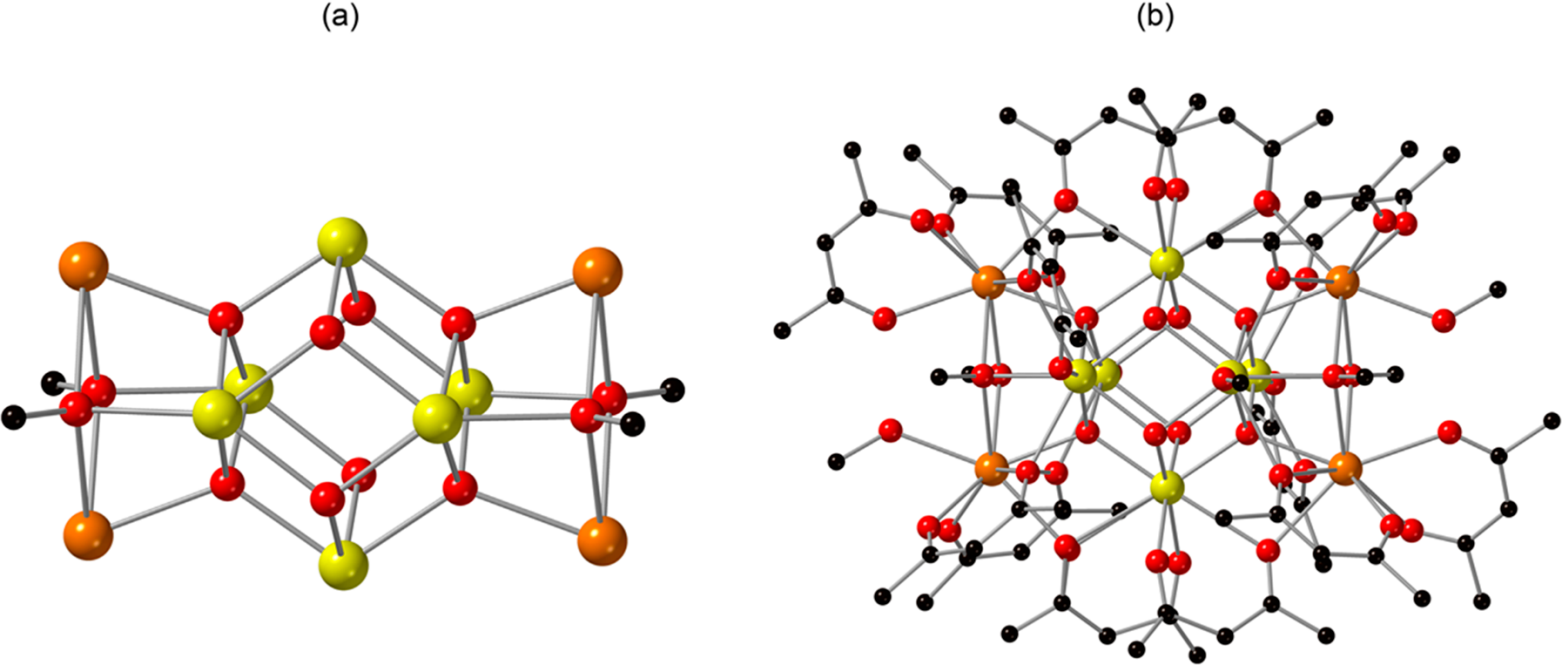
Illustration of the Ce_10_ cluster of **Ce-10** (a) highlighting the [Ce_6_^IV^Ce_4_^III^O_8_(Ome)_4_]^16+^ core; Ce^III^ and Ce^IV^ sites are shown in orange and yellow, respectively, and (b) showing the acac^1–^ decorated [Ce_10_O_8_(acac)_14_(CH_3_O)_6_(CH_3_OH)_2_] cluster. Ce is shown in yellow and orange, O in red, and C in black.

Compound **Ce-10**, [Ce_10_O_8_(acac)_14_(CH_3_O)_6_(CH_3_OH)_2_]·10.5 MeOH, crystallized in the *P*2_1_*/n* space group. Five crystallographically unique Ce sites constitute the structure of **Ce-10**. Further details on metal ion coordination numbers and Ce–O bond lengths are provided in the Supporting Information (Table S2). Overall, the structure is built from a decameric [Ce_6_^IV^Ce_4_^III^O_8_(OMe)_4_]^16+^ cluster core (Figure 2), wherein ten Ce sites are bridged by eight μ_3_/μ_4_-oxo dianions and four μ_3_-OMe^1–^ monoanions. Bond valence summation values, which are the sum of the individual valences for an atom that add up to the oxidation state,^54^ calculated for the **Ce-10** cluster were consistent with six Ce^IV^ and four Ce^III^ sites (Table S7). The Ce^IV^ sites are located at the center of the Ce_10_ core and adopt an oxo-bridged hexanuclear moiety with six Ce^IV^ cations connected by eight oxo-ligands; related structural units have been reported for both trivalent (e.g., Bi and Ce) and tetravalent (e.g., Zr, Hf, Th, U, Np, Pu) metal ions.^35,55−70^ By comparison, the Ce^III^ sites form methoxy-bridged dinuclear [Ce_2_(OMe)_2_] species and two of these structural units “cap” the hexamer via bridging μ_4_-oxo groups to generate the decameric cluster shown in Figure 2. Fourteen acac^1–^ ligands, two OMe^1–^, and two MeOH ligands bind the cluster core (Figure 2b).

**Figure 3:**
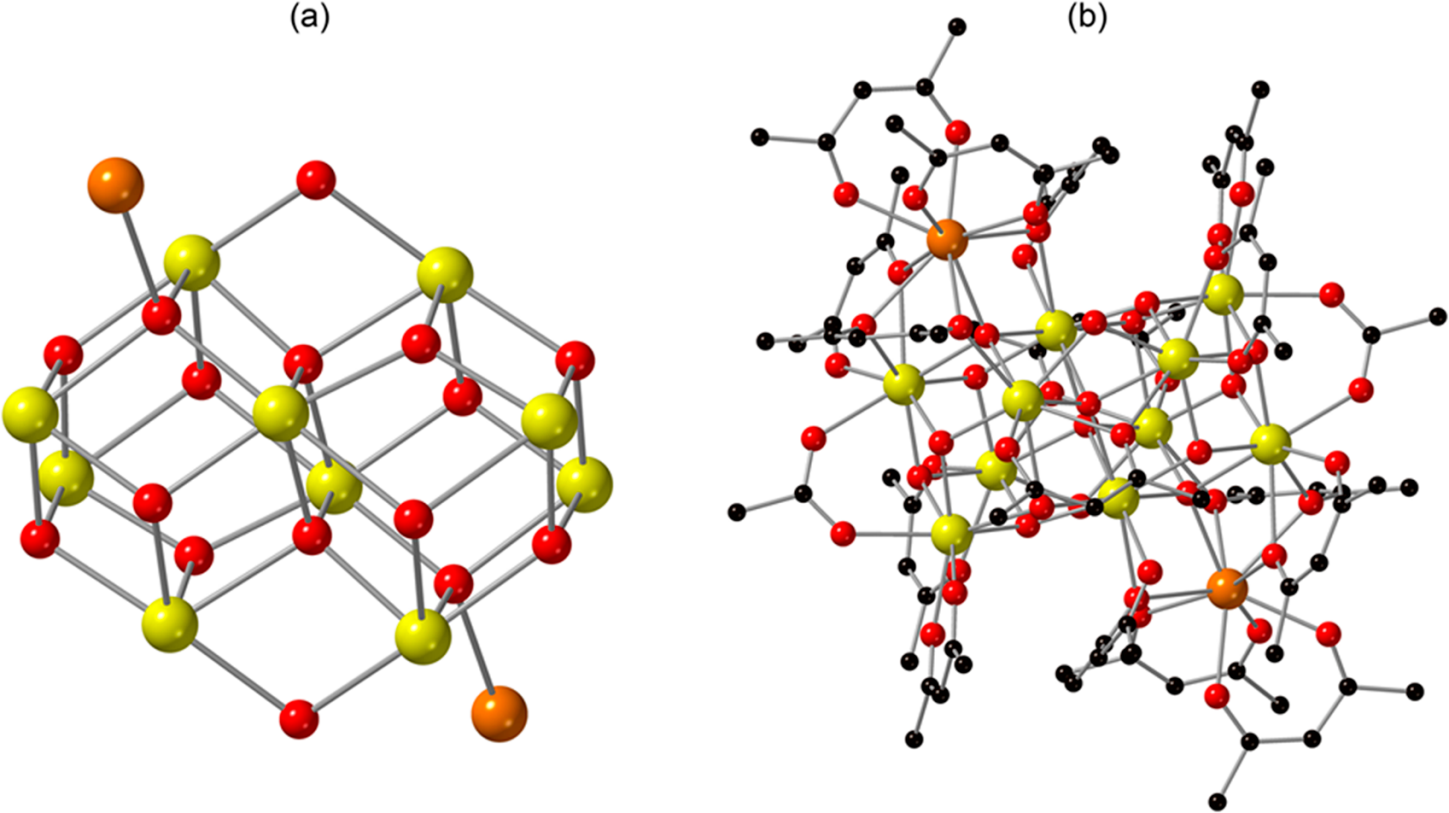
Illustration of **Ce-12** highlighting (a) the [Ce^IV^_10_Ce^III^_2_O_12_(OH)_4_]^18+^ cluster core that consists of both Ce^III^ (orange) and the Ce^IV^ (yellow) sites and (b) the acac^1–^ and acetate decorated [Ce_12_O_12_(OH)_4_(acac)_16_(CH_3_COO)_2_] cluster. Ce is shown in yellow and orange, O in red, and C in black. Acetonitrile molecules that reside in the lattice have been omitted for clarity.

Dissolution of **Ce-10** in acetonitrile reproducibly generated compound **Ce-12**, [Ce_12_O_12_(OH)_4_(acac)_16_(CH_3_COO)_2_]·6(CH_3_CN), which crystallized in the triclinic space group, *P-1*. Overall, the structure is composed of a Ce_12_-oxo/hydroxo cluster, [Ce^IV^_10_Ce^III^_2_O_12_(OH)_4_]^18+^, that is ligated by 16 acac^1–^ and two acetate groups to give a neutral cluster (Figure 3). Within the cluster, there are six crystallographically unique Ce sites, one of which (Ce1) is Ce^III^ and five of which (Ce2–Ce6) are Ce^IV^ based on BVS values (Table S10). As shown in Figure 3a, the five Ce^IV^ metal centers, together with their symmetry equivalent sites, are bridged through two μ_2_-hydroxo, two μ_3_-hydroxo, ten μ_3_-oxo, and two μ_4_-oxo groups to form a decameric unit that can be described as two edge-sharing Ce_6_ octahedra. These octahedra are linked through two μ_4_-oxo sites. The two Ce^III^ metal centers are located at opposite sides of the Ce_10_ core (related by an inversion center) to yield the Ce_12_ structural unit; the Ce^III^ cations bind through two μ_3_-oxo ions (of the Ce_10_ unit), which become μ_4_-oxo via coordination of the Ce^III^ sites. Location of the Ce^III^ metal centers at the periphery of the cluster is consistent with previous reports of Ce cluster chemistry,^24,71^ as well as the structure of compound **Ce-10**. As shown in Figure 3b, the [Ce^IV^_10_Ce^III^_2_O_12_(OH)_4_]^18–^ cluster is further coordinated to 16 acac^1–^ and two acetate ligands, with the latter presumably forming *in situ* through oxidative cleavage of the acac^1–^.^72^ Additionally, six acetonitrile molecules are present in the lattice (Figure S11). Further details on metal ion coordination numbers, Ce–O bond distances, and Ce–OH bond lengths are provided in the Supporting Information (Table S3).

#### Relationship to Other Ce-Oxo Clusters

Cerium cluster chemistry has seen a significant expansion over the past two decades.^18,20,24,73−78^ The **Ce-10** and **Ce-12** clusters exhibit unique arrangements of Ce metal centers that previously have not been reported. Nonetheless, some features of the clusters compare well with those of other known clusters. As summarized in the Supporting Information (Table S12), the most common core motif reported for Ce is the hexameric unit of composition [Ce_6_O_4_(OH)_4_]^12+^.^14,18,24,77,79^ This cluster has most frequently been isolated using carboxylate donors, and the Ce sites are usually tetravalent. Notably, the pervasiveness of the hexanuclear entity is similarly reflected in the cluster chemistry of other +3 and +4 metal ions including Bi^III^, Zr^IV^, Hf^IV^, Th^IV^, U^IV^, Np^IV^, and Pu^IV^.^35,64,65,80^

The Ce_6_ octahedral core manifests in both the **Ce-10** and **Ce-12** clusters reported herein. As noted above, the Ce_10_ assembly in **Ce-10** consists of a central Ce^IV^_6_ oxo bridged moiety that is effectively capped by two methoxy bridged Ce^III^ dimers to generate a decamer. By comparison, **Ce-12** is composed of edge-sharing Ce^IV^ octahedra; two Ce^III^ monomers are located at opposite ends of the core. The observation of the Ce_6_ unit as a component of larger assemblies is similarly reflected in the literature. For example, Christou et al. recently reported (pyH)_8_[Ce_10_O_4_(OH)_4_(O_3_PPh)_12_(NO_3_)_12_] that consisted of a face capped [Ce_6_O_4_(OH)_4_]^12+^ core.^77^ Additionally, Loiseau et al. reported a Ce dodecamer, [{Ce_6_(μ_3_-O)_4_(μ_3_–OH)_3_(μ_4_-O)_2_-Ce_6_(μ_3_-O)_4_(μ_3_OH)_3_}(CH_3_COO)_13_(SiW_9_O_34_)_2_]^11–^, that is perhaps best described as two hexameric units bridged via oxo groups.^81^

In addition to previously reported homometallic Ce_10_ decamers,^77^ which exhibit different arrangements of Ce atoms as compared to **Ce-10**, there are few heterometallic assemblies that consist of Ce_10_ assemblies together with other metal ions such as Mn and Na.^20,82−84^ These Ce_10_ cluster cores vary in the arrangement of the Ce metal centers and range from edge-sharing Ce_6_ octahedra to three Ce_3_ units surrounding a central Ce site.^20,82−84^ Interestingly, the homo- and heterometallic structures that consist of edge-sharing Ce_6_ octahedra, such as (pyH)_8_[Ce_10_O_4_(OH)_4_(O_3_PPh)_12_(NO_3_)_12_] and [Ce_10_Mn_14_O_24_(O_2_CPh)_32_], adopt the same decameric unit that is observed in **Ce-12**. Such units have likewise been observed for the actinides.^85,86^ For example, [U_10_O_8_(OH)_6_(PhCO_2_)_14_I_4_(H_2_O)_2_(MeCN)_2_] consists of edge-sharing octahedra.^86^ Dodecameric species have previously been reported for lanthanide ions including Pr, Nd, Gd, and Dy.^87−91^ Yet there are only a handful of homo- and heterometallic Ce_12_ units reported.^5,81,87,88,92^ Perhaps most notably, Ce_12_(C_2_)_3_I_17_ adopts a similar core to that observed in compound **Ce-12**.^87^ However, although the cluster in **Ce-12** exists as an isolated structural unit, Ce_12_(C_2_)_3_I_17_ adopts extended chains with C_2_ units in interstitial positions.

Finally, it is worth noting the location of the Ce^III^ and Ce^IV^ sites in the clusters. It is fairly well established in the cerium literature that for mixed oxidation state clusters, Ce^III^ and Ce^IV^ tend to locate to the cluster surface and core, respectively.^18^ Indeed, the oxidation state assignments of the Ce sites in **Ce-10** and **Ce-12** based on bond valence summation values are consistent with literature precedence.^71^ For **Ce-10**, the Ce^IV^ sites form a hexanuclear core that is capped by Ce^III^ dimers. Compound **Ce-12** has a similar arrangement of Ce^III^ and Ce^IV^ sites, with ten Ce^IV^ forming edge-sharing octahedra, and two Ce^III^ located at the periphery of the cluster. This tendency is also manifested in heterometallic clusters. For example, Gupta et al. characterized [Ce_6_Mn_12_O_17_(O_2_CPh)_26_], which consisted of four {Mn_3_(μ_3_-O)_2_} surrounding a Ce^IV^_6_ core.^93^ Likewise, Thuijs et al. reported [Ce_3_Mn_8_O_8_(O_2_CPh)_18_(HO_2_CPh)_2_] for which the central position in the cluster core was occupied by Ce^IV^ and the surface was composed of two Ce^III^ and eight Mn^III^ sites.^94^ Nonetheless, it is important to note that this is not the only arrangement observed. For example, Kögerler et al. reported a Ce decamer that consisted of Ce^IV^ cations surrounding a central Ce^III^.^20^

### Synthetic Considerations

An interesting attribute associated with the formation of **Ce-10** was that its formation was dependent on the solvent identity and the cerium starting material used in the reaction. For example, monomeric Ce(acac)_4_ was isolated from ethanolic solutions. Meanwhile, **Ce-10** that consists of methoxy bridged units was isolated from methanolic solutions. We rationalized these results based on the realization that methanol is more acidic than ethanol, which can explain why a methoxide is formed and the ethoxide equivalent does not form.^95^ Interestingly, the **Ce-10** cluster was pervasive across a range of reaction conditions as detailed in the Supporting Information. The structural unit was found to precipitate irrespective of the Ce source. For example, Ce^III^-chloride, nitrate, triflate, and sulfate salts as well as Ce^IV^(SO_4_)_2_ all generated the **Ce-10** cluster. Thus, **Ce-10** formed irrespective of the Ce oxidation state in the starting materials, with oxidation of Ce^III^ possibly occurring by action of ambient O_2_,^96^ and reduction of Ce^IV^ plausibly occurring via oxidation of acac^1–^, as reported previously.^97,98^ In addition to **Ce-10**, two other structures built from **Ce-10** were isolated (see Supporting Information). These phases differ primarily in packing due to differences in solvent inclusion into the lattice. Finally, **Ce-12** was prepared through dissolution of **Ce-10** in MeCN, and efforts to synthesize the phase from cerium salts were unsuccessful. The ^1^H NMR and SAXS data are consistent with rearrangement of the structural units in solution and ligand dissociation from the cluster. Peaks in the ^1^H NMR spectrum (Figure S22; Table S6) are observed at approximately 3.6, 5.45, and 5.6 ppm and are attributed to free Hacac (3.6 and 5.6 ppm) and bound acac^1–^ (5.45 ppm, 5.6 ppm).^99^ The presence of acetate ligands in **Ce-12** may result from oxidative cleavage of acac^1–^, *in situ*. Such oxidative cleavage has previously been reported in MeCN using mild catalysts such as quaternary ammonium iodide and H_2_O_2_.^72^ The oxidative cleavage of acac^1–^ to yield acetate in this work points to the reactivity of the Ce clusters.

### Vibrational Spectroscopy

The infrared (IR) spectra for **Ce-10** (Figure S20) and **Ce-12** (Figure S21) are reported. The spectra are dominated by vibrations attributed to acac^1–^. Assignments are provided in Tables S4 and S5.

### ^1^H Nuclear Magnetic Resonance Spectroscopy

A ^1^H NMR spectrum was collected for the solution obtained by adding **Ce-10** to deuterated acetonitrile (Figure S22), mimicking the procedure used to obtain **Ce-12**. The peak observed at approximately 3.6 ppm is consistent with the keto form of free Hacac, and the peak at 5.45 ppm is assigned to bound acac^1–^. The peak at approximately 5.6 ppm may be attributed to the enol tautomer of Hacac or bound acac^1–^. These assignments point toward evidence of ligand dissociation of **Ce-10** when dissolved in solution, and SAXS, discussed below, points to species larger than **Ce-10** or **Ce-12** in solution. Crytals of **Ce-12** precipitate from the solution, along with an amorphous material that is likely cerium oxyhydroxide.

### Small Angle X-ray Scattering (SAXS)

**Figure 4:**
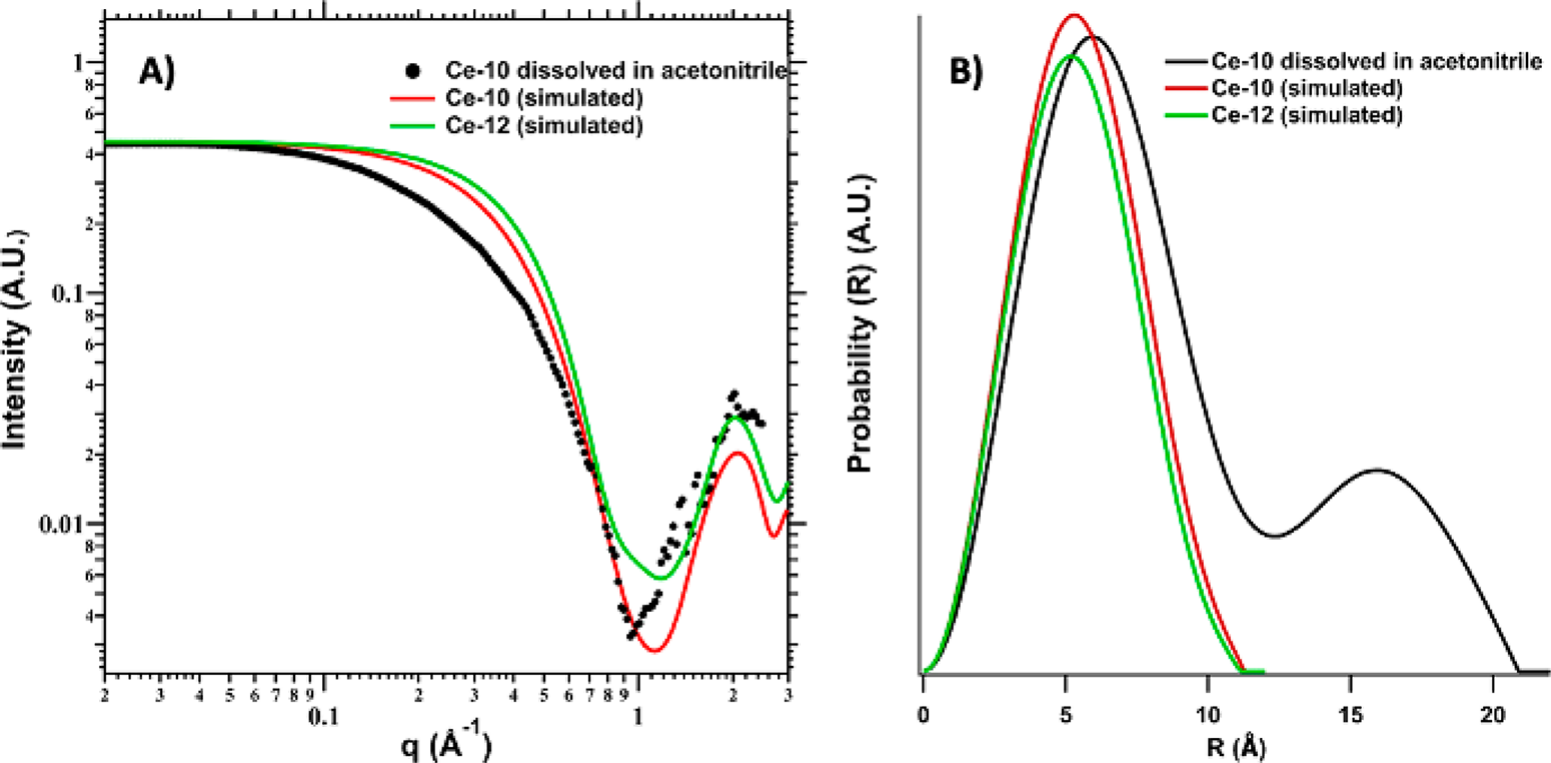
(a) Scattering spectra from **Ce-10** dissolved in acetonitrile, along with simulated scattering for **Ce-10** and **Ce-12**. (b) Pair distance distribution function (PDDF) of the experimental and simulated scattering data.

We conducted SAXS experiments to determine if any information could be gleaned about the stability of **Ce-10** in acetonitrile and/or the formation of **Ce-12**, which (along with amorphous precipitate) was deposited from the MeCN solution of **Ce-10**. Certainly, rearrangement is necessary given the differences in topology and the differences in the Ce^IV^:Ce^III^ ratio of **Ce-10** and **Ce-12**. Comparison of the experimental scattering data for the **Ce-10**/MeCN solution and the simulated scattering for both **Ce-10** and **Ce-12** suggest that **Ce-10** reacts in MeCN to make clusters that are larger than **Ce-10** and **Ce-12**. As shown in Figure 4a, the simulated scattering curves of **Ce-10** and **Ce-12** are similar given the similar size and shape of the clusters. Yet the simulated scattering curve for **Ce-10** suggests a slightly larger size (based on the slight shift to lower q of the Guinier elbow at *q* ≈ 0.32 Å^–1^), despite its smaller nuclearity. In fact, for **Ce-12**, the distance between the periphery Ce^III^ ions is 10.8 Å. On the other hand, comparing the longest Ce–Ce distance within the **Ce**_**10**_ core of **Ce-12** (8.2 Å) to the longest Ce–Ce distance of **Ce-10** (9.3 Å) shows that the **Ce-10** core is actually bigger. This is because the **Ce-10** core contains six Ce^IV^ and four Ce^III^, while the **Ce-12** core contains ten Ce^IV^, with commensurate shorter Ce–O bond distances, on average. Clearly there is a mismatch in the Guinier region between the experimental scattering and the simulated scattering for both **Ce-10** and **Ce-12**; the experimental scattering shows a shift to lower *q*, indicating a larger average size species than both of the crystallized clusters or some aggregation. Fourier transform of the scattering data gives a pair distance distribution function (PDDF) representation (Figures 4b and S19), which is a probability distribution map of scattering vectors through the scattering species, and also provide shape information based on characteristic PDDF profiles.^100^

The PDDFs of the simulated **Ce-10** and **Ce-12** give Gaussian distribution of scattering vectors, as expected for approximately spherical, dense particles, and respective radii of gyration (*R*_g_) of 4.15 and 4.05 Å (Figure S19). *R*_g_ is the size-independent, root–mean–squared average of the electrons from the center of the cluster, similar to the radius and related by ∼√(5/3)*R*_g_ = radius for a spherical particle. The distance (*R*) where the probability goes to zero is ∼11 Å for both **Ce-10** and **Ce-12**, consistent with the longest Ce–O distance within the cluster core. On the other hand, the PDDF for **Ce-10** dissolved in acetonitrile is consistent with the presence of clusters associated as dimers in solution, and an average *R*_g_ of 6.9 Å.^100^ This sort of association is reminiscent of V_10_ dimerization via H-bonding, observed by SAXS in solution (also observed in the solid state).^101^ Because the acac^1–^ ligands are noted to dissociate from the cluster core via ^1^H NMR in acetonitrile, we suspect the SAXS data indicates that cluster–cluster association in solution is important to the **Ce-10** to **Ce-12** conversion.

### Electrospray Ionization Mass Spectrometry (ESI-MS)

**Figure 5:**
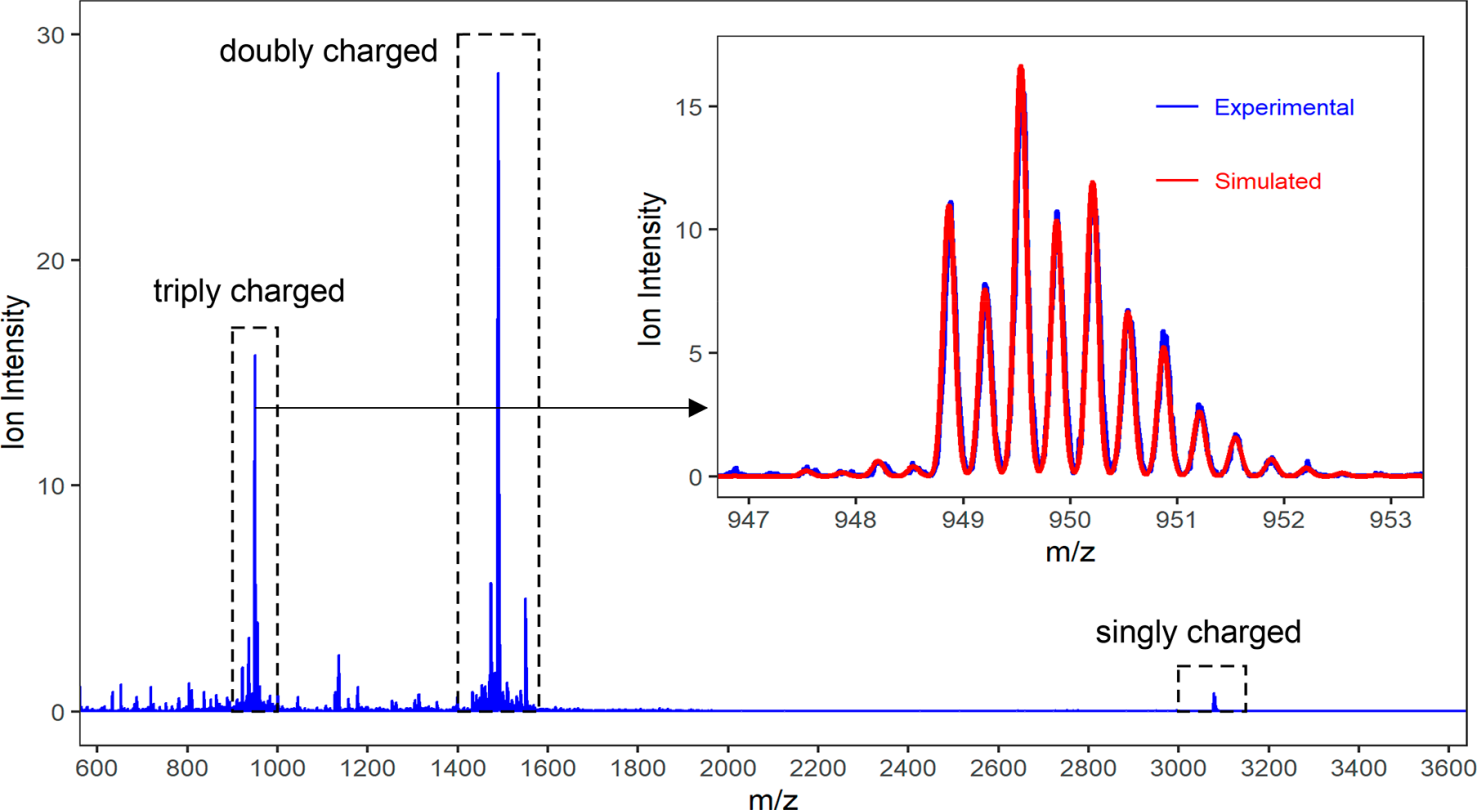
Mass spectra from 600 to 3600 *m*/*z* of 50 μM **Ce-10** in a MeCN solution. The boxes with dotted lines highlight the singly, doubly, and triply charged groups of ions. The inset shows the major triply charged ion with a monoisotopic peak at *m*/*z* 948.87, with the experimental data from the **Ce-10** solution in blue and the simulated data of **Core Ce-10(3+)** in red.

To further interrogate the identity of the Ce species present in solution upon the dissolution of **Ce-10** in MeCN, ESI-MS data were collected (Figure 5). Ions above 600 *m*/*z* were attributed to Ce clusters based on isotopic patterns. Three prominent groups of peaks were observed in the spectrum: peaks around *m*/*z* 950, around *m*/*z* 1490, and around *m*/*z* 3080 corresponded to triply, doubly, and singly charged ions, respectively, based on isotopic pattern spacings. Examination of the ions within these groups showed peaks that were spaced by mass differences corresponding to water and methanol. This was interpreted as the presence of clusters that differed in the number of OH^–^, MeO^–^, and O^2–^ ligands. To find potential molecular formulas, a combinatorial search was conducted using constraints of 5–15 cerium nuclearity, 7–30 acac ligands, 0–20 OH^–^ ligands, 0–20 O^2–^ ligands, and 0–20 MeO^–^ ligands. Only formulas that satisfied the ion charge (based on a combination of Ce^III^ and Ce^IV^) and were within 30 ppm of the experimental monoisotopic mass were retained.

For the major triply charged ion with monoisotopic peak at *m*/*z* 948.87 (Figure 5 inset), the constraints above resulted in 14 formulas, among which 3 were Ce_11_ and 11 were Ce_10_, indicating more likelihood of Ce_10_ clusters. Moreover, Ce_11_ clusters in the original list of 14 had poorer isotopic matching with the experimental data compared to that of Ce_10_ clusters. Further filtering the list using a constraint of 6 Ce^IV^ centers based on crystallographic data resulted in 4 formulas, all of which were Ce_10_ clusters (Table S11). Accordingly, it was concluded that the triply charged ion at *m*/*z* 948.8753 represents a Ce_10_ cluster. The inset of Figure 5 depicts isotopic envelope matching between the experimental data and one of the 4 potential formulas: Ce_10_(CH_3_COCHCOCH_3_)_12_(OH)_5_O_7_(CH_3_O)_2_^3+^ denoted as **Core Ce-10(3+)**. The doubly and singly charged ion groups in Figure 5 can be interpreted in relation to the triply charged ion at *m*/*z* 948.8753 via variations in number of ligands and are further discussed in the Supporting Information (Figures S23–S25).

### X-ray Absorption Spectroscopy (XAS)

**Figure 6:**
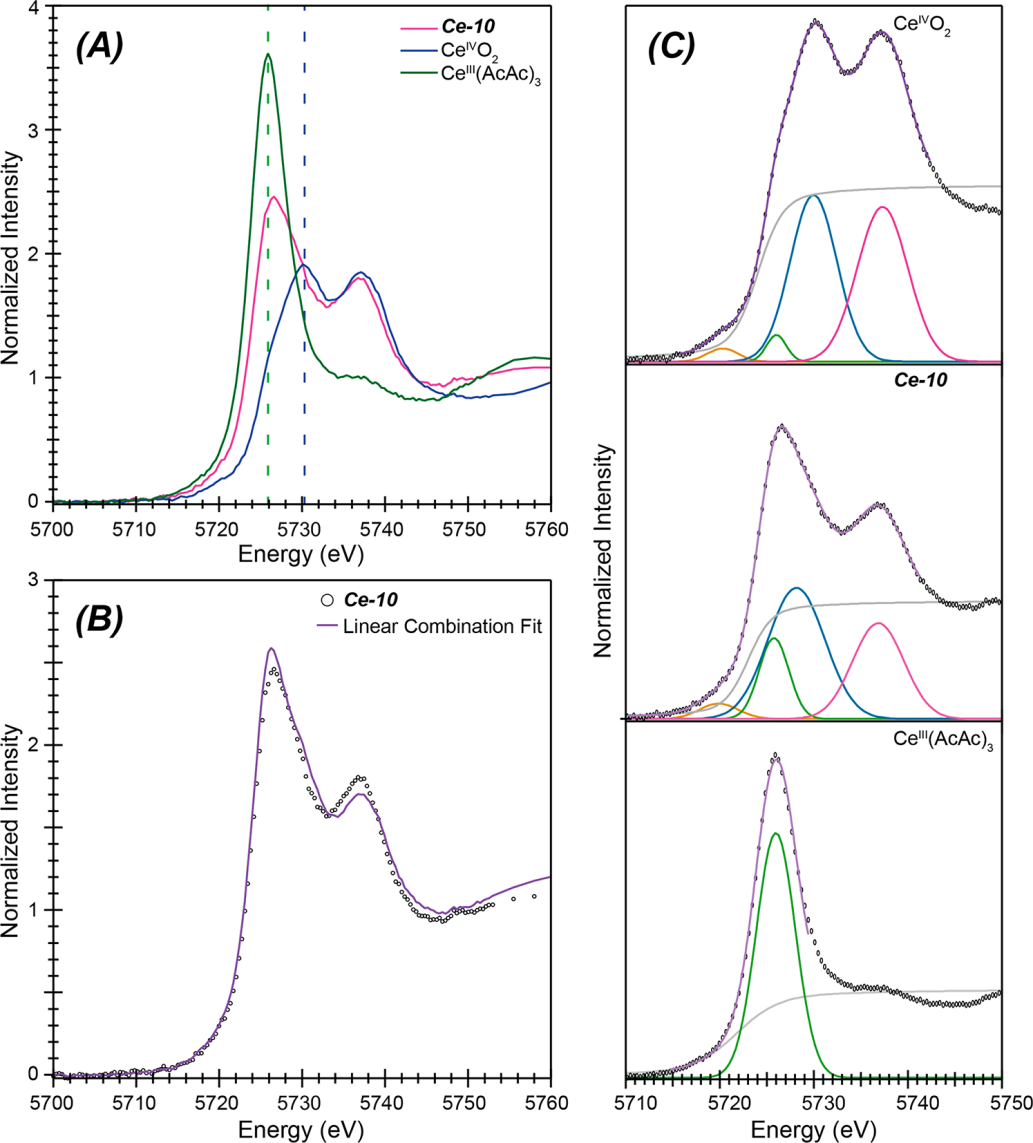
(A) Room temperature background subtracted and normalized Ce L_3_-edge XAS spectra (298 K) from **Ce-10** (pink trace). For comparison, spectra collected under analogous conditions from +4 and +3 cerium oxidation state standards were included, namely Ce^IV^O_2_ (blue trace) and Ce^III^(acac)_3_ (green trace). (B) The normalized Ce L_3_-edge spectrum from **Ce-10** (○) and a linear combination analysis fit (purple trace) comprised contributions from the +4 and +3 cerium oxidation state standards, specifically Ce^IV^O_2_ and Ce^III^(acac)_3_. Contributions from the +4 standard were 56(2)% and contributions from the +3 standard were 44(2)%. (C) Deconvolution of room temperature, background subtracted, and normalized Ce L_3_-edge spectra from CeO_2_ (top), **Ce-10** (middle), and Ce(acac)_3_ (bottom). Experimental data (○) were overlaid on the fit (purple trace) and functions used to generate the model. These included Gaussian functions (brown, green, blue, and pink traces) used to model the X-ray absorption peaks and a step function (1:1 combination of an arctangent and error function; gray trace).

**Ce-10** was examined via Ce L_3_-edge X-ray absorption spectroscopy (XAS). Note that limited sample size or impurities precluded similar investigations for **Ce-1** and **Ce-12**. The data were comparatively evaluated against two oxidation state standards: ceria (Ce^IV^O_2_) and cerium(III) acetylacetonate [Ce^III^(acac)_3_] (Figure 6a). The spectrum from **Ce-10** was hybridic in nature, meaning that it had attributes associated with both oxidation state standard extremes. It showed the double white line characteristic of cerium in a +4 oxidation state. However, the rising edge inflection point 5724.1(1) eV and peak maximum 5726.7(1) eV for the first feature were lower in energy than we expected for a Ce^IV^ complex and suggested Ce^III^. Another metric for deciphering Ce^III^ vs Ce^IV^ content was the branching ratio between the two absorption features of the double white line peak: .^24,102−106^ The +4 oxidation state standard, Ce^IV^O_2_, had a branching ratio of 0.51. In contrast, the double white line branching ratio (determined via curve fitting analysis) for **Ce-10** was 0.67. The high branching ratio in **Ce-10** reflected that the low energy absorption feature was substantially more intense than the high energy feature. Higher branching ratios can be indicative of compounds that contain both Ce^III^ and Ce^IV^, which was consequently how we interpreted this spectrum.^107^

In **Ce-10**, there are eight oxo dianions, 14 acac^1–^ monoanions, and six methoxide ligands; the total number of negative charges is 36. The presence of a double white line absorption feature in the Ce L_3_-edge X-ray absorption spectrum unambiguously refuted homovalent Ce^III^ models of the spectrum. It was also difficult to rationalize that the XAS data could originate from a cluster that only had Ce^IV^ cations because of (1) the low energy associated with the rising edge inflection point, (2) the low energy for the first peak maximum, and (3) the relative intensities from the two features (high branching ratio). Instead, the experiments suggested that two contributions to the Ce_10_ XAS spectrum existed: one from cerium in the +3 oxidation state and another from cerium in the +4 oxidation state. Under the aforementioned designation (with six methoxide ligands), there was one way to charge balance and generate a neutral cluster: four Ce^III^ and six Ce^IV^. We fit the data from **Ce-10** as a summation of spectra from the two oxidation state standards to test the validity of this description. This was achieved using the following equation:
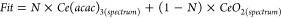


Here, *Ce*(*acac*)_3(*spectrum*)_ was the L_3_-edge XAS data from Ce(acac)_3_, *CeO*_2(*spectrum*)_ was the L_3_-edge XAS data from CeO_2_, and *N* was a linear combination mixing coefficient. The resulting model fit excellently the features, relative intensities, and overall line shape of the Ce L_3_-edge XAS data from **Ce-10** (*R*-factor = 3.6%). The model consisted of a 44(2)% contribution from the +3 standard [Ce(acac)_3_] and 56(2)% contribution from the +4 standard CeO_2_ (Figure 6b). These experimentally determined +3 vs +4 contribution values were equivalent to the charge balanced description of **Ce-10** mentioned above and the bond valence summation values from the structural analysis: four Ce^III^ cations and six Ce^IV^ cations.

## Conclusion

A discrete Ce-oxo cluster, Ce_10_O_8_(acac)_14_(CH_3_O)_6_(CH_3_OH)_2_]·10.5 MeOH (**Ce-10**), and a monomeric molecule, Ce(acac)_4_, were isolated from methanol and ethanol solutions, respectively. Dissolution of **Ce-10** in acetonitrile led to the isolation of another discrete Ce-oxo cluster, [Ce_12_O_12_(OH)_4_(acac)_16_(CH_3_COO)_2_](MeCN)_6_ (**Ce-12**). Bond valence summation performed for both clusters showed Ce^III^/Ce^IV^ mixed oxidation state cluster cores, with XAS data confirming this assignment for the Ce-10 cluster. The stability of **Ce-10** in solution was probed using SAXS and ESI-MS. The ESI-MS exhibits peaks consistent with decanuclear (Ce_10_) species. SAXS identifies the presence of clusters in solution (either **Ce-10** and/or **Ce-12**) that are associated via dispersive or hydrophobic interactions, which may be important for the conversion of **Ce-10** to **Ce-12**. Overall, this study points to the utility of β-diketonate ligand scaffolds in stabilizing novel cluster cores and the role that solvent identity has on cluster formation. Note, in methanol, we isolated methoxy bridged polynuclear species, and in ethanol, we isolated monomeric structural units. Given ongoing interest in Ce-oxo cluster chemistry in catalysis application spaces and for advancing fundamental insight into lanthanide and actinide oxo cluster chemistry, our ongoing efforts center on identifying solution stable species and probing their chemical reactivity and catalytic behavior. Additionally, we are excited about the prospect of extending these results to actinide systems and characterizing similarities and/or differences between Ce and Pu cluster chemistry.

## Supporting Information Available

Crystallographic refinement details, thermal ellipsoid plots, packing diagrams for **Ce-1**, **Ce-10**, and **Ce-12**, descriptions of Ce coordination chemistry in **Ce-10** and **Ce-12**, powder X-ray diffraction patterns, small-angle X-ray scattering PDDF, IR and NMR spectra, bond valence summation values, XAS sample preparation, instrument configuration, data analysis, summary of previously reported homometallic cerium-oxo clusters (PDF)
