# Supplementary material for: Synthesis and Characterization of Cerium-Oxo Clusters Capped by Acetylacetonate
Source: Inorg Chem. 2023 Oct 4;63(21):9406–17. doi: 10.1021/acs.inorgchem.3c02141 (PMC11134509; doi:10.1021/acs.inorgchem.3c02141)
Supplement: Supplementary file 1 — ic3c02141_si_001.pdf [file ic3c02141_si_001.pdf]

SUPPORTING INFORMATION

Synthesis and Characterization of Cerium-Oxo Clusters Capped by Acetylacetonate

Anamar Blanes-Díaz,[1] Mohammad Shohel,[2] Natalie T. Rice,[3] Ida Piedmonte,[3] Morgan A. McDonald,[1] Kaveh Jorabchi,[1] Stosh A. Kozimor,[3] Jeffery A. Bertke,[1] May Nyman,[2] Karah E. Knope[1,]*

[1] Department of Chemistry, Georgetown University, 37th and O Streets NW, Washington, D.C. 20057, USA
[2] Department of Chemistry, Oregon State University, Corvallis, OR 97331, USA
[3] Los Alamos National Laboratory (LANL), P.O. Box 1663, Los Alamos, New Mexico 87545, USA

*To whom correspondence should be addressed: kek44@georgetown.edu

I. Summary of various synthetic conditions that yielded Ce-10, [$Ce_{10}O_8(acac)_{14}(CH_3O)_4(CH_3OH)_4$] 2
II. Crystallographic Refinement Details 4
III. ORTEP diagrams for Ce-1, Ce-10 and Ce-12 6
IV. Packing diagrams for Ce-1, Ce-10 and Ce-12 9
V. Description of Ce Coordination Chemistry in Ce-10 and Ce-12 17
VI. Powder X-ray diffraction (PXRD) patterns 20
VII. Small-angle X-ray scattering (SAXS) 26
VIII. Infrared (IR) spectra 27
IX. ^{1}H NMR spectrum of Ce-10 30
X. BVS for compounds Ce-10, Ce-10.2, Ce-10.3 and Ce-12 31
XI. X-ray Absorption Spectroscopy (XAS) Sample Preparation 33
XII. X-ray absorption spectroscopy (XAS) instrument configuration and data analysis 33
XIII. Electrospray Ionization Mass Spectrometry 35
XIV. Homometallic Ce-oxo clusters 38
XV. References 42

I. SUMMARY OF VARIOUS SYNTHETIC CONDITIONS THAT YIELDED CE-10, $[CE_{10}O_8(ACAC)_{14}(CH_3O)_4(CH_3OH)_4]$

All the reaction conditions listed below reproducibly yielded compound **Ce-10** in different yields and purity.

Materials

The materials used for the syntheses that follow were $Ce(NO_3)_3{\bullet}6H_2O$ (ACROS Organics), $CeCl_3$ (STREM CHEMICALS), $Ce_2(SO_4)_3{\bullet}8H_2O$ (Aldrich Chemical Company, Inc.), $Ce(CF_3O_3S)_3$ (STREM CHEMICALS), acetylacetone (TCI America), methanol (Fisher Chemical), 200 proof ethanol (The Warner-Graham Company), triethylamine (Sigma-Aldrich), acetonitrile (Fisher Chemical), and hexanes (Fisher Chemical). All chemicals were purchased from commercial suppliers and used as they were received.

$CeCl_3$

$CeCl_3$ (0.2465 g , 1 mmol), and 204 μL acetylacetone (2 mmol) were added to 3 mL of MeOH in a 7 mL glass vial. The solution was sonicated for 5 minutes until the $CeCl_3$ had dissolved. Once dissolved, 557 μL triethylamine (4 mmol) were added dropwise to the solution, yielding an orange color solution that was then capped and the reaction cooled to 15 °C. After 24 hrs, the mother liquor was removed, and orange crystals remained in the vial. The crystals were washed with 3X 3 mL of a 50/50 MeOH/hexanes mixture, and they were left to dry for an hour. The structure was determined to be **Ce-10** via single crystal X-ray diffraction (scXRD). Powder diffraction on the bulk sample showed a mixture of compounds **Ce-10** and **Ce-10.3** (see below).

$Ce(NO_3)_3$

$Ce(NO_3)_3{\bullet}6H_2O$ (0.3262 g, 1 mmol), and 204 μL acetylacetone (2 mmol) were added to 3 mL of MeOH in a 7 mL glass vial. The solution was sonicated for 5 minutes until the $Ce(NO_3)_3$ had dissolved. Once dissolved, 557 μL triethylamine (4 mmol) were added dropwise to the solution, yielding a dark orange color solution that was then capped and the reaction cooled to 15 °C. After 24hrs, the mother liquor was removed, and orange crystals remained in the vial. The crystals were washed with 3X 3 mL of a 50/50 MeOH/hexanes mixture, and they were left to dry for an hour. The structure was determined to be **Ce-10** via single crystal X-ray diffraction (scXRD). The bulk sample was pure by pXRD.

$Ce_2(SO_4)_3$

$Ce_2(SO_4)_3{\bullet}8H_2O$ (0.3322 g, 1 mmol), and 204 μL acetylacetone (2 mmol) were added to 3 mL of MeOH in a 7 mL glass vial. The solution was sonicated for 5 minutes. Once dissolved, 557 μL triethylamine (4 mmol) were added dropwise to the solution, yielding an orange color solution that was then capped and the reaction cooled to 15 °C. After 24hrs, the mother liquor was removed, and orange crystals remained in the vial amongst undissolved $Ce_2(SO_4)_3{\bullet}8H_2O$. The crystals were washed with 3X 3 mL of a 50/50 MeOH/hexanes mixture, and they were left to dry for an hour. The structure was determined to be **Ce-10.3** via single crystal X-ray diffraction (scXRD).

Ce(III)triflate

$Ce(CF_3O_3S)_3$ (0.5873 g, 1 mmol), and 204 µL acetylacetone (2 mmol) were added to 3 mL of MeOH in a 7 mL glass vial. The white solution was sonicated for 5 minutes. Afterwards, 557 µL triethylamine (4 mmol) were added dropwise to the solution, creating a brown solution that layered on top of the white solution. The reaction vial was then capped, and the reaction was cooled to 15 °C for 24hrs. The mother liquor was removed, and orange crystals remained in the vial along with brown precipitate. The product, which contained crystals, was washed with water (3X 1 mL) and MeOH (1X 1 mL) and left to dry in ambient conditions for an hour. The structure was determined to be **Ce-10** via single crystal X-ray diffraction (scXRD).

$Ce(NO_3)_3$

$Ce(NO_3)_3$•$6H_2O$ (0.3262 g, 1 mmol), and 204 µL acetylacetone (2 mmol) were added to 3 mL of MeOH in a 7 mL glass vial. The solution was sonicated for 5 minutes until the $Ce(NO_3)_3$ had dissolved. Once dissolved, 557 µL triethylamine (4 mmol) were added dropwise to the solution, turning it to a dark orange color. Afterwards, 5 µL H_2O was added to the solution. The reaction vial was capped, and the reaction cooled to 15 °C. After 24hrs, the mother liquor was removed, and orange crystals remained in the vial. The crystals were washed with 3X 3 mL of a 50/50 MeOH/hexanes mixture, and they were left to dry for an hour. The structure was determined via single crystal X-ray diffraction (scXRD) to be $[Ce_{10}O_8(acac)_{14}(CH_3O)_6(CH_3OH)_2]$ (**Ce-10.2**) that crystallized with the unit cell a = 19.5145(9) Å, b = 21.4075(10) Å, c = 25.5733(13) Å, α = β = γ = 90°, V = 10683.4(9) $Å^3$.

$Ce(NO_3)_3$

$Ce(NO_3)_3$•$6H_2O$ (0.3262g, 1 mmol), and 204 µL acetylacetone (2 mmol) were added to 3 mL of MeOH in a 7 mL glass vial. The solution was sonicated for 5 minutes until the $Ce(NO_3)_3$ had dissolved. Once dissolved, 2 mL of KOH were added dropwise to the solution, yielding a dark orange solution that was then capped and the reaction cooled to 15 °C. After 24hrs, the mother liquor was removed, and orange crystals remained in the vial covered in a thin layer of orange precipitate. The crystals were washed with 3X 3 mL of a 50/50 MeOH/hexanes mixture, and they were left to dry for an hour. The structure was determined to be **Ce-10** via single crystal X-ray diffraction (scXRD).

$Ce(NO_3)_3$

$Ce(NO_3)_3$•$6H_2O$ (0.3262g, 1 mmol), and 204 µL acetylacetone (2 mmol) were added to 3 mL of MeOH in a 7 mL glass vial. The solution was sonicated for 5 minutes until the $Ce(NO_3)_3$ had dissolved. Once dissolved, 557 µL triethylamine (4 mmol) were added dropwise to the solution, yielding a dark orange solution. Afterwards, 1mL THF was added to the solution, creating a yellow precipitate. The solution was then capped and cooled to 15 °C for 24hrs. The mother liquor was removed, and orange crystals remained in the vial with a yellow precipitate. The structure was determined to be **Ce-10** via single crystal X-ray diffraction (scXRD).

$Ce(NO_3)_3$

$Ce(NO_3)_3$•$6H_2O$ (0.3262g, 1 mmol), and 256 µL acetylacetone (2.5 mmol) were added to 10 mL of MeOH in a 40 mL glass vial. The solution was sonicated for 5 minutes until the $Ce(NO_3)_3$ had dissolved. Once dissolved, 700 µL triethylamine (5 mmol) were added dropwise to the solution,

yielding a dark orange color. The solution was then capped and cooled to 15 °C for 6 days. The mother liquor was removed, and orange crystals remained in the vial. The crystals were washed with 3X 3 mL of a 50/50 MeOH/hexanes mixture, and they were left to dry for an hour. The structure was determined to be **Ce-10** via single crystal X-ray diffraction (scXRD).

Note that the same general synthetic protocol as that described for **Ce-10**, but at room temperature, yielded a polymorph of **Ce-10.2**, [$Ce_{10}O_8(acac)_{14}(CH_3O)_6(CH_3OH)_2$] (**Ce-10.3**). A preliminary structure refinement is provided as Supplemental Information. Cell parameters for this phase are: a = 15.0944(8) Å, b = 22.5302(12) Å, c = 15.6923(7) Å, α = 90 °, β = 101.044(2) °, γ = 90 °. The PXRD is shown in Figure S6.

Ce(SO$_4$)$_2$

$Ce(SO_4)_2$ (0.3322 g, 1 mmol) and 204 µL of acetylacetone (2 mmol) were dissolved in 3 mL of MeOH. The solution was sonicated for 5 minutes, with some undissolved $Ce(SO_4)_2$ left at the bottom of the vial. After sonicating, 557 µL of triethylamine (4 mmol) were added dropwise, creating a bottom layer of yellow powder and a solution in the top layer that was a yellow brown color. The solution was cooled to 15 °C and, after 7 days, crystals were noticed in the vial. The crystals were confirmed to be **Ce-10.2** by SCXRD.

II. CRYSTALLOGRAPHIC REFINEMENT DETAILS

Ce(acac)$_4$ (Ce-1)

A structural model consisting of 1/2 of the target $Ce(acac)_4$ molecule per asymmetric unit was developed. Methyl H atom positions, R-CH_3, were optimized by rotation about R-C bonds with idealized C-H, R--H and H--H distances. The remaining H atoms were included as riding idealized contributors. Methyl H atom U's were assigned as 1.5 times U_{eq} of the carrier atom; remaining H atom U's were assigned as 1.2 times carrier U_{eq}.

[Ce$_{10}$O$_8$(acac)$_{14}$(CH$_3$O)$_4$(CH$_3$OH)$_4$] (Ce-10)

A structural model consisting of the compound plus 10.5 disordered methanol solvate molecules was developed; however, positions for the idealized solvate molecules were poorly determined. Since positions for the solvate molecules were poorly determined a second structural model was refined with contributions from the solvate molecules removed from the diffraction data using the bypass procedure in PLATON (Spek, 2015). No positions for the compound network differed by more than two su's between these two refined models. The electron count from the "squeeze" model converged in good agreement with the number of solvate molecules predicted by the complete refinement. The "squeeze" data are reported here.

Three of the acac ligands are disordered over two positions. The like O-C and C-C distances were restrained to be similar (esd 0.01 Å). The atoms of one disordered orientation were restrained to be relatively planar (esd 0.01 Å). One coordinated methanol is disordered over two positions. The like O-C and Ce-O distances were restrained to be similar. The methyl group of one coordinated methanol is disordered over two orientations. The like O-C distances were restrained to be similar (esd 0.01\%A). Similar displacement amplitudes (esd 0.01) were imposed on disordered sites overlapping by less than the sum of van der Waals radii.

The hydroxyl H atoms for the methanol molecule could not be located in the difference map and thus were left off of the model. Methyl H atom positions, R-CH_3, were optimized by rotation about R-C bonds with idealized C-H, R--H and H--H distances. Remaining H atoms were included as riding idealized contributors. Methyl H atom U's were assigned as 1.5 times U_{eq} of the carrier atom; remaining H atom U's were assigned as 1.2 times carrier U_{eq}.

$[Ce_{12}O_{12}(OH)_4(acac)_{16}(CH_3COO)_2](CH_3CN)_6$ (Ce-12)

A structural model consisting of the compound and six acetonitrile solvate molecules was developed. The acetonitrile molecule was disordered across a symmetry site; therefore, it was refined with negative PART commands. Methyl H atom positions, R-CH_3, were optimized by rotation about R-C bonds with idealized C-H, R--H and H--H distances. Remaining H atoms were included as riding idealized contributors. Methyl H atom U's were assigned as 1.5 times U_{eq} of the carrier atom; remaining H atom U's were assigned as 1.2 times carrier U_{eq}.

Table S1. Crystallographic details for compound **Ce-10.2** and **Ce-10.3**

	**Ce-10.2**	**Ce-10.3**
Formula	$C_{78}H_{122}Ce_{10}O_{44}$	$C_{78}H_{122}Ce_{10}O_{44}$
MW (g mol^{-1})	3164.95	3164.95
T (K)	100	100
crystal color/ habit	orange block	orange plate
crystal system	Orthorhombic	monoclinic
λ (Å)	0.71073	0.71073
Space group	Pbca	$P2_1/n$
a (Å)	19.5145(9)	15.0941(7)
b (Å)	21.4075(10)	22.5292(12)
c (Å)	25.5733(13)	15.6918(7)
α (°)	90	90
β (°)	90	101.047(2)
γ (°)	90	90
V (Å^3)	10683.4(9)	5237.2(4)
Z	4	2
ρ (mg m^{-3})	1.968	2.007
μ (mm^{-1})	4.246	4.331
R_1	0.0514	0.0429

wR_2	0.0811	0.1104
GOF	1.131	1.065
CCDC	2257006	2257003

III. ORTEP DIAGRAMS FOR CE-1, CE-10 AND CE-12

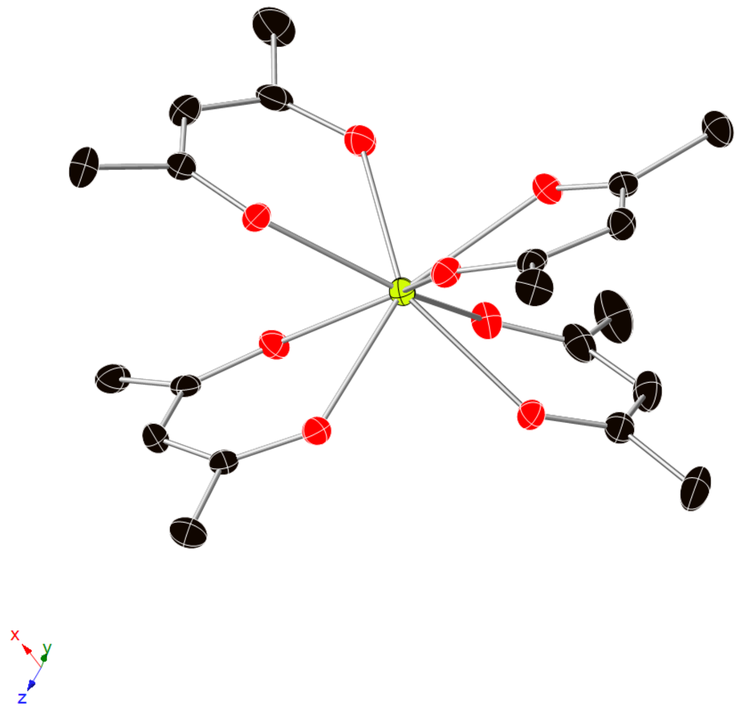

Figure S1. Thermal ellipsoid plot (50 % probability level) of **Ce-1** at 100 K.

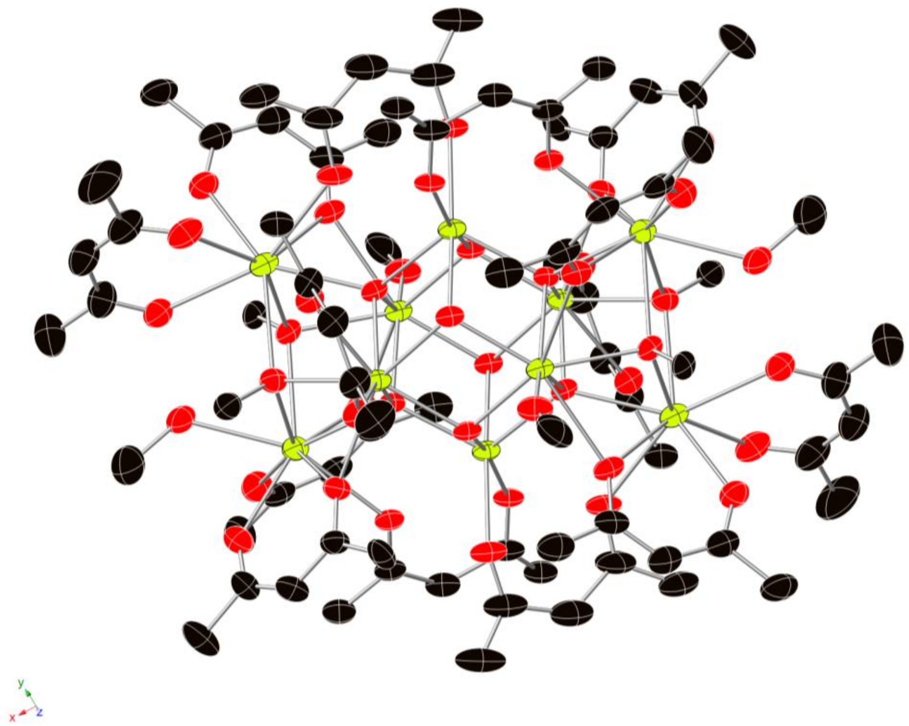

Figure S2. Thermal ellipsoid plot (50% probability level) of **Ce-10** at 100 K.

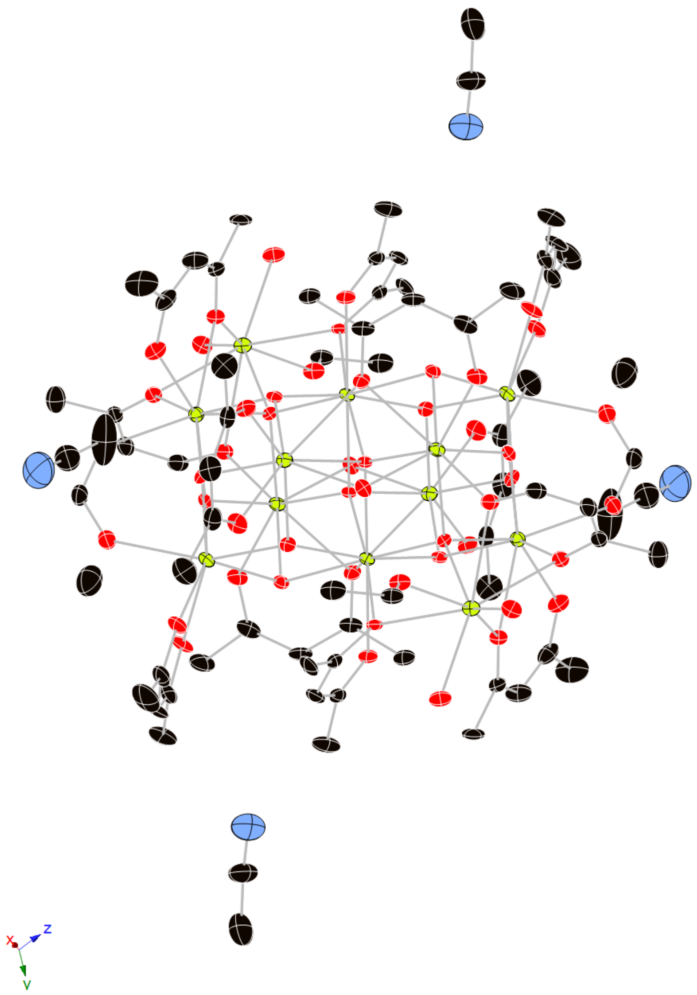

Figure S3. Thermal ellipsoid plot (50% probability level) of **Ce-12**, with 4 ACN in the outer coordination sphere, at 100 K.

IV. PACKING DIAGRAMS FOR CE-1, CE-10 AND CE-12

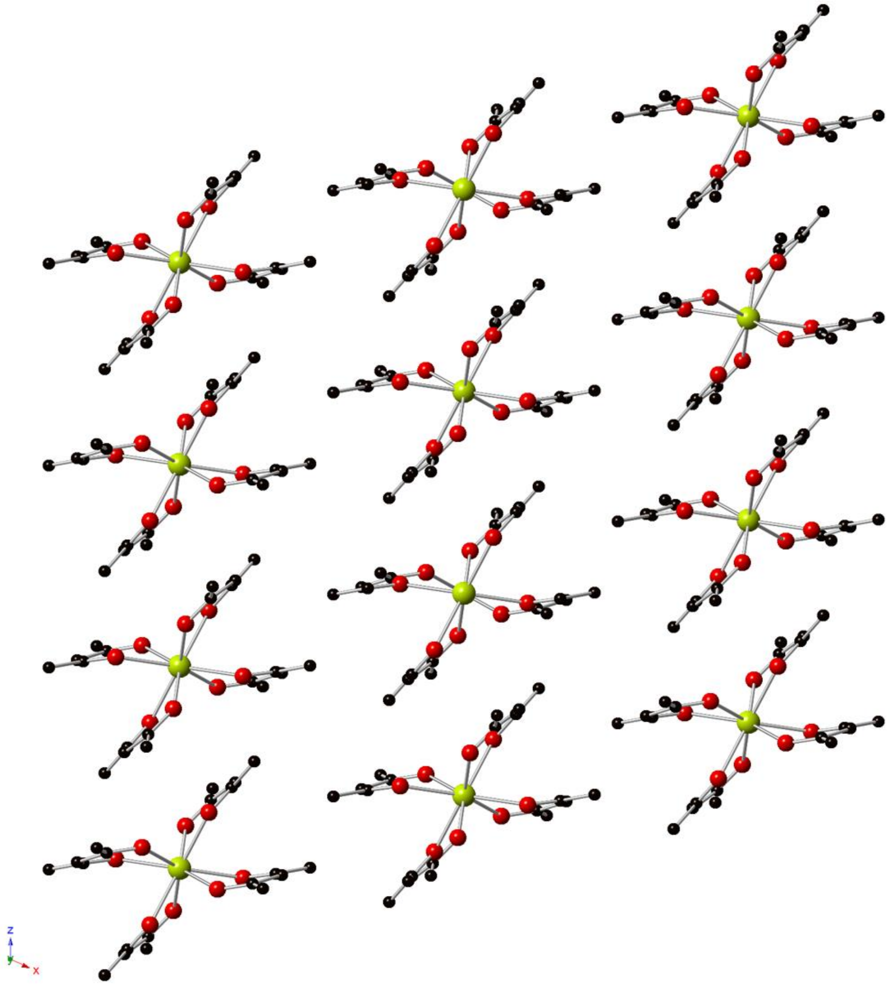

Figure S4. Packing diagram of **Ce-1** viewed down the [010]. The Ce atoms are yellow-green, O atoms in red, and C atoms in black.

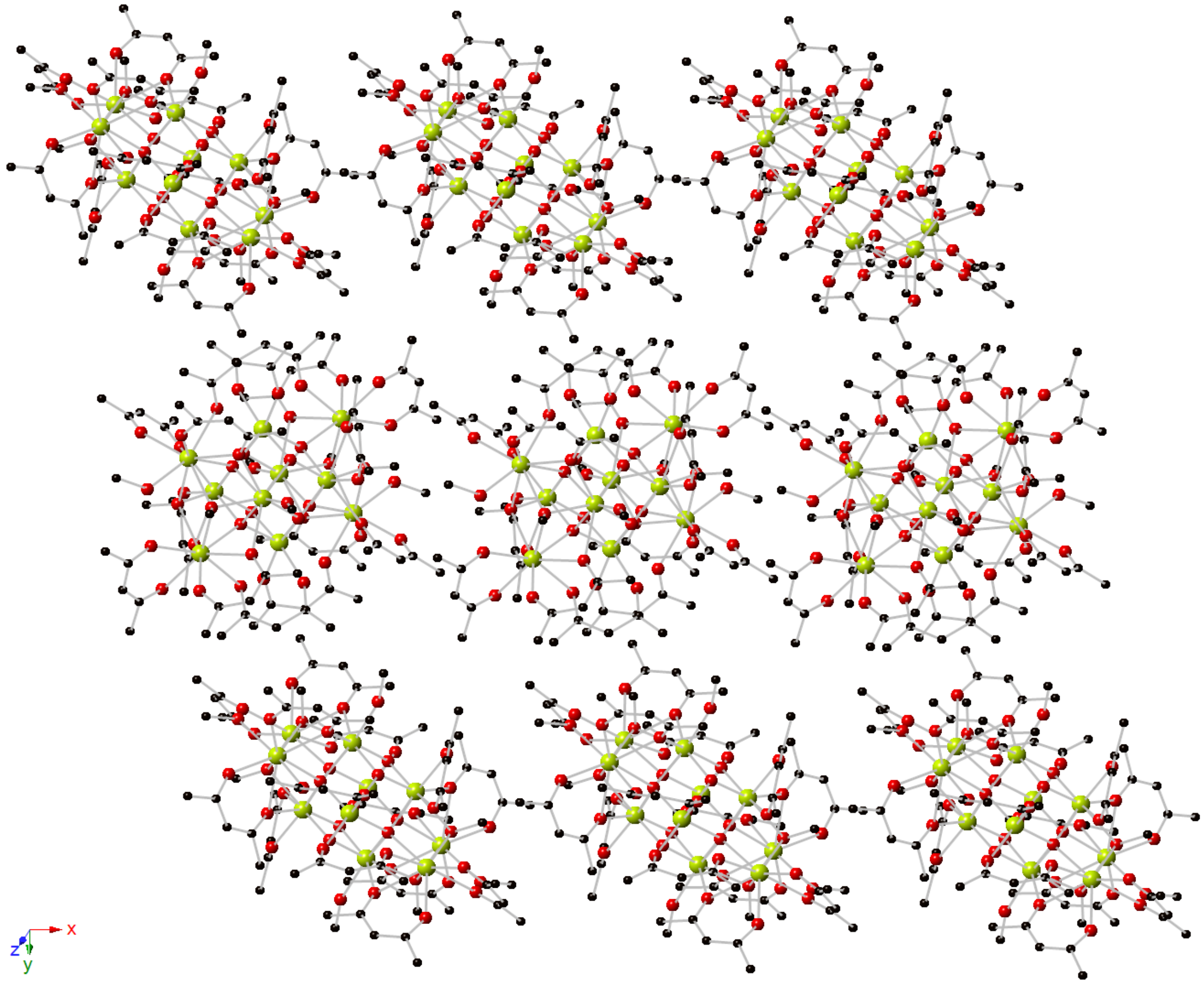

Figure S5. Packing diagram of **Ce-10**. The Ce atoms are in yellow-green, O in red and C in black.

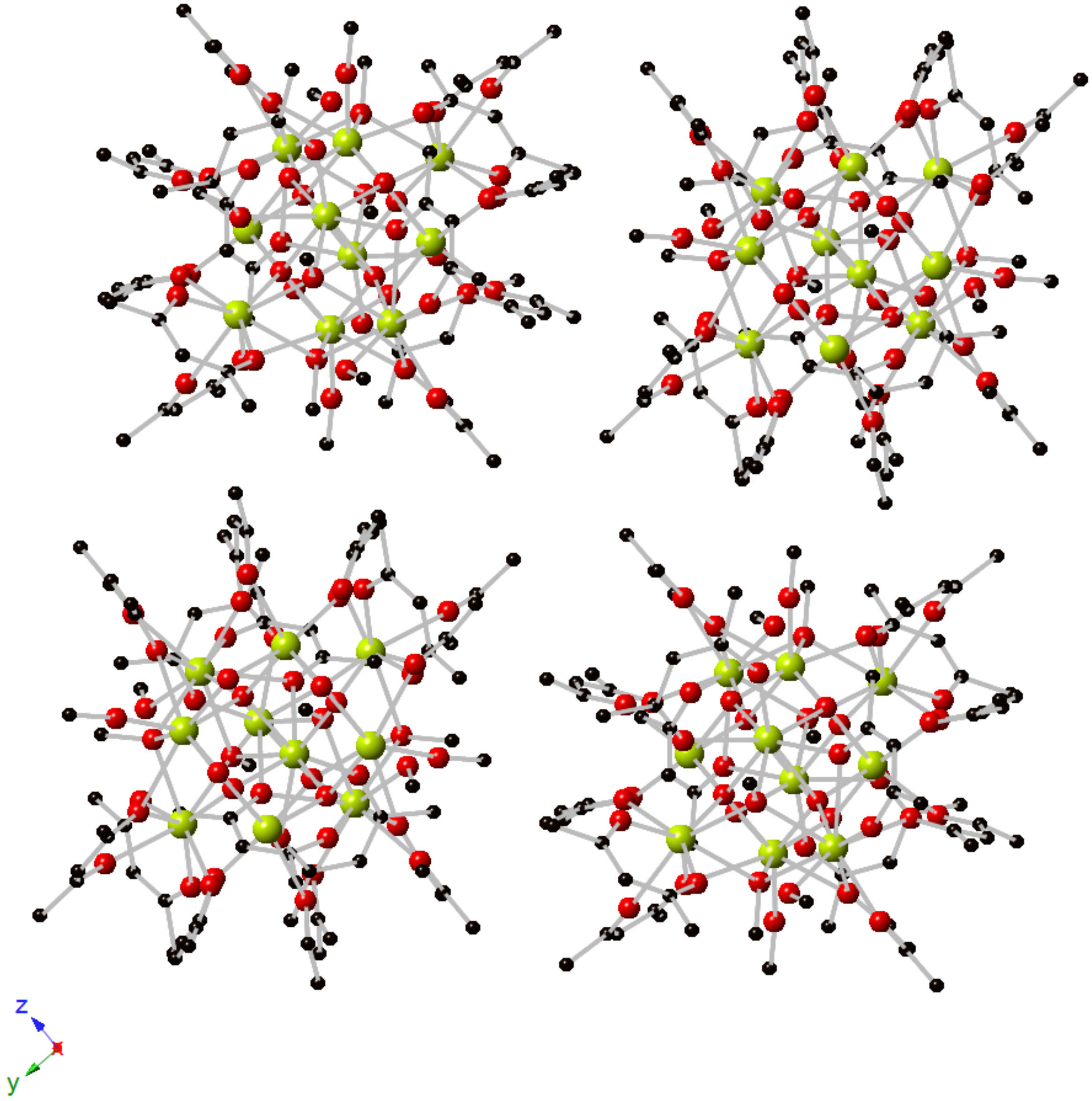

Figure S6. Packing diagram of **Ce-10** viewed down [100]. The Ce atoms are in yellow-green, O in red and C in black.

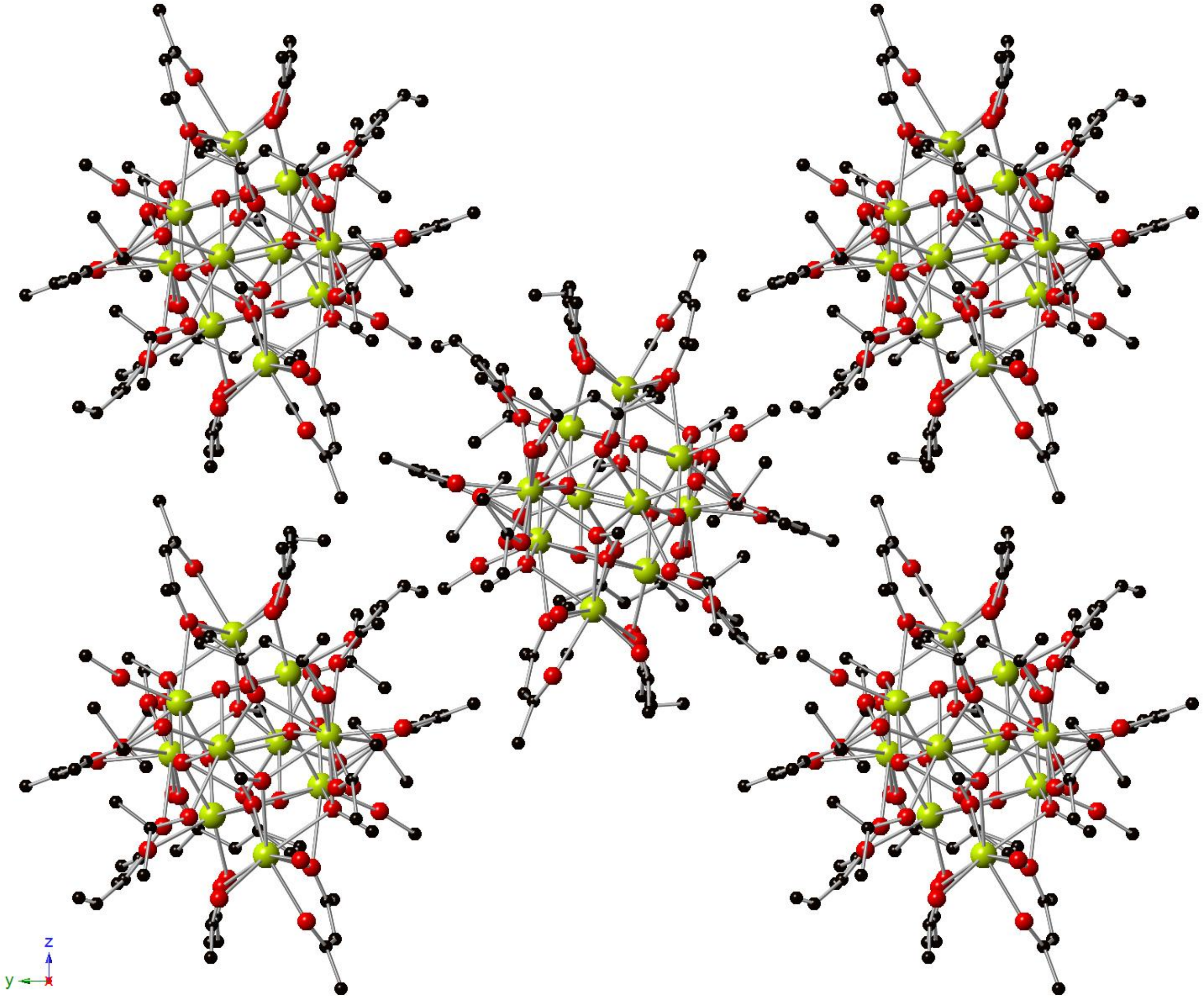

Figure S7. Packing diagram of **Ce-10.3** looking down the x-axis. The Ce atoms are in yellow-green, O in red and C in black. A different packing is seen compared to **Ce-10**.

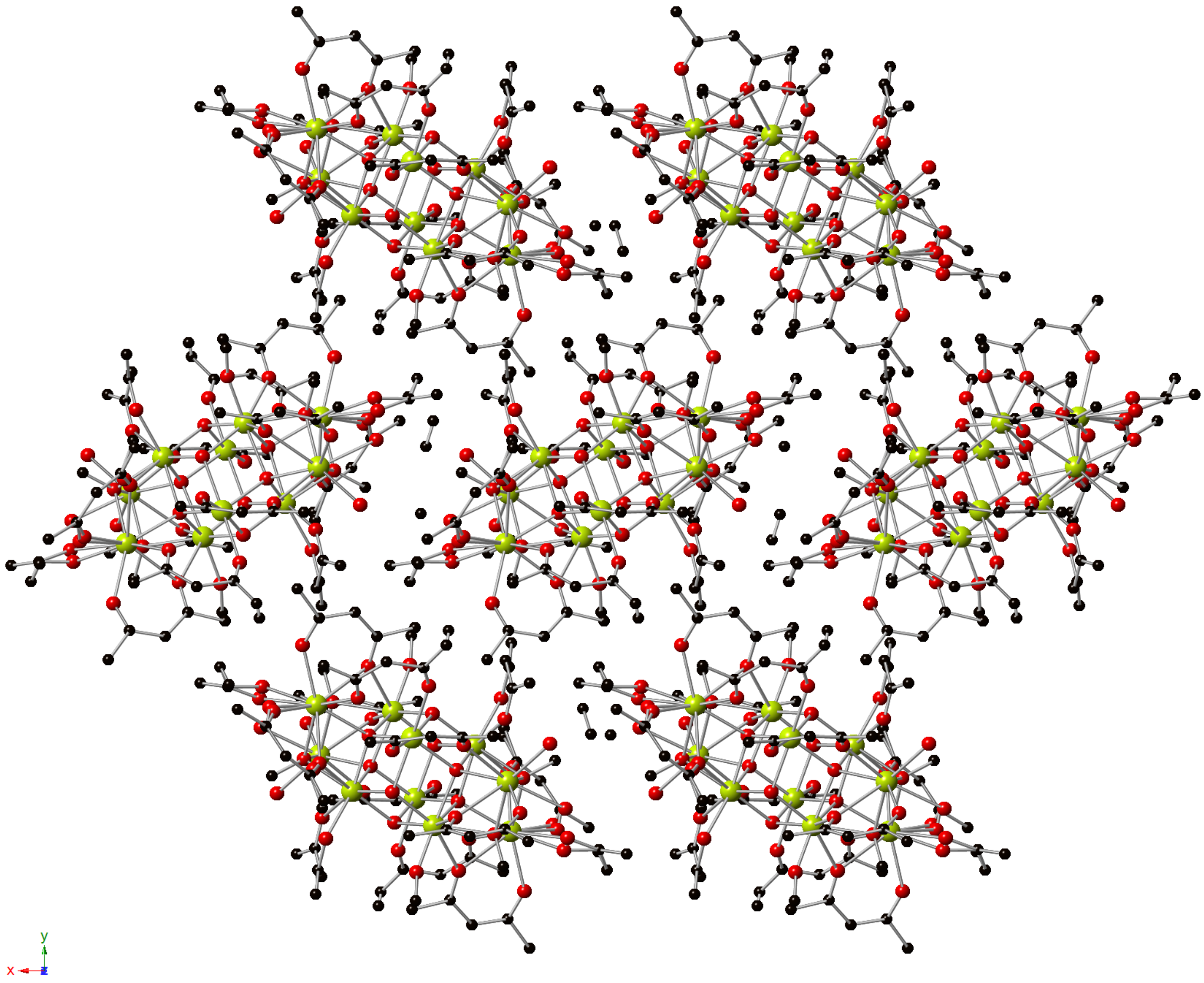

Figure S8. Packing diagram of **Ce-10.3** shown down [001]. The Ce atoms are in yellow-green, O in red and C in black.

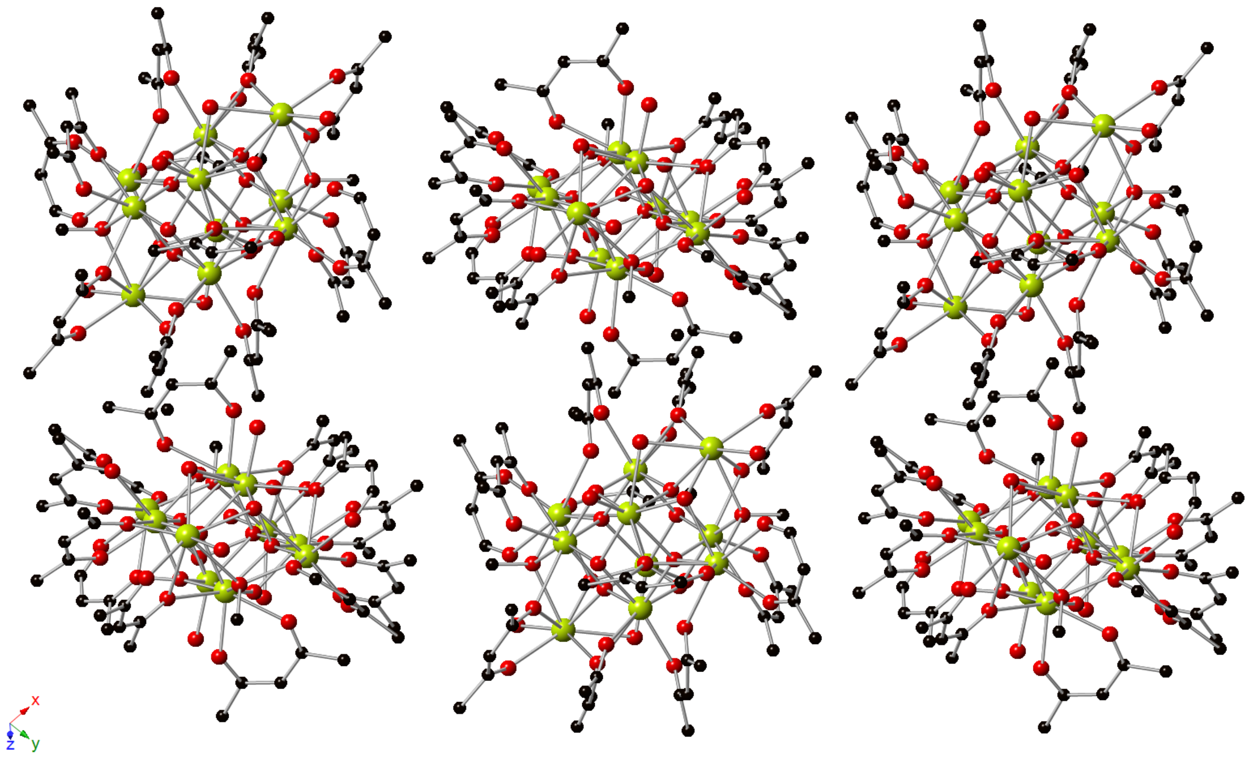

Figure S9. Packing diagram of compound **Ce-10.2**. The Ce atoms are in yellow-green, O in red and C in black.

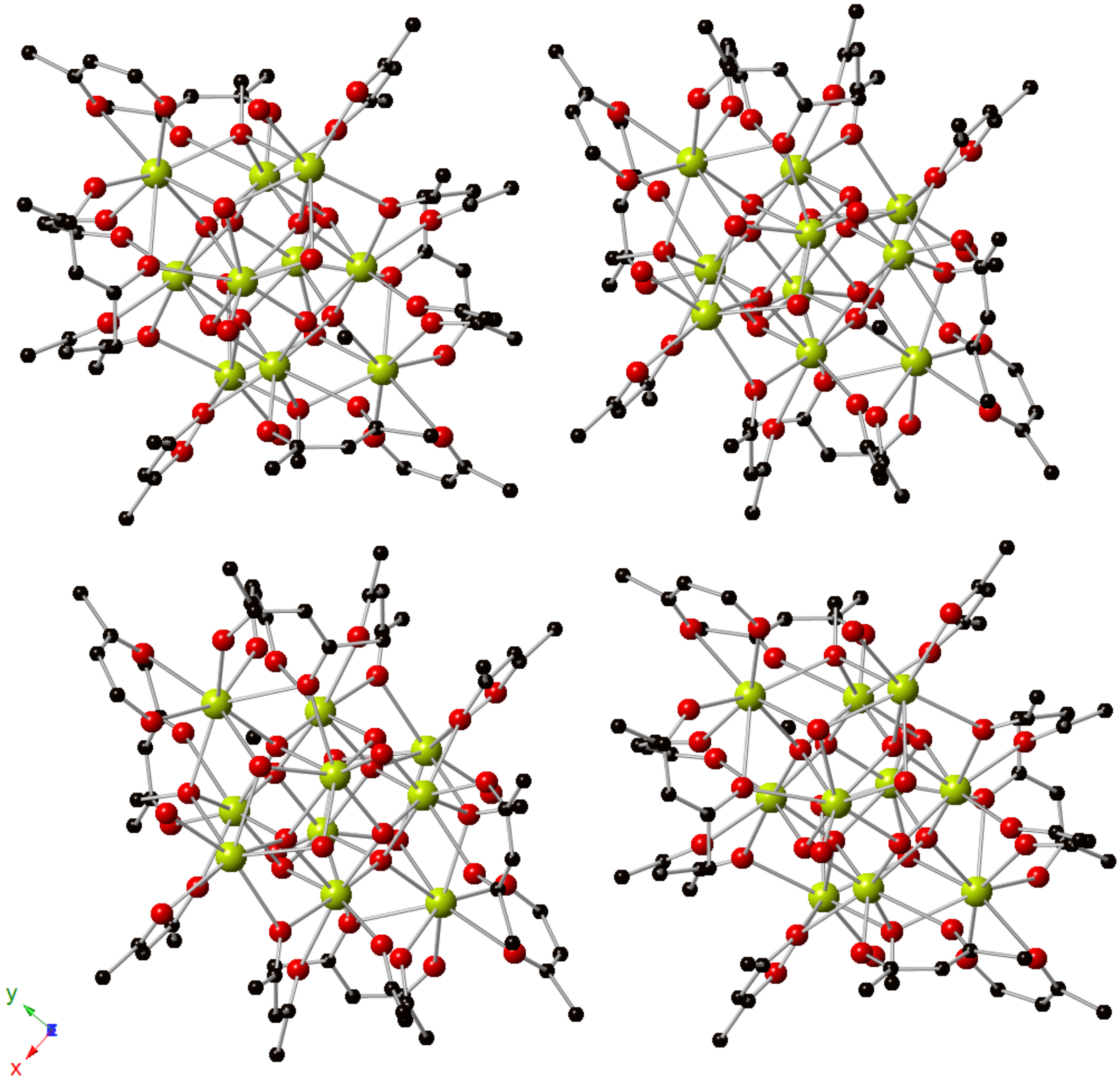

Figure S10. Packing diagram of compound **Ce-10.2** viewed down [001]. The Ce atoms are in yellow-green, O in red and C in black.

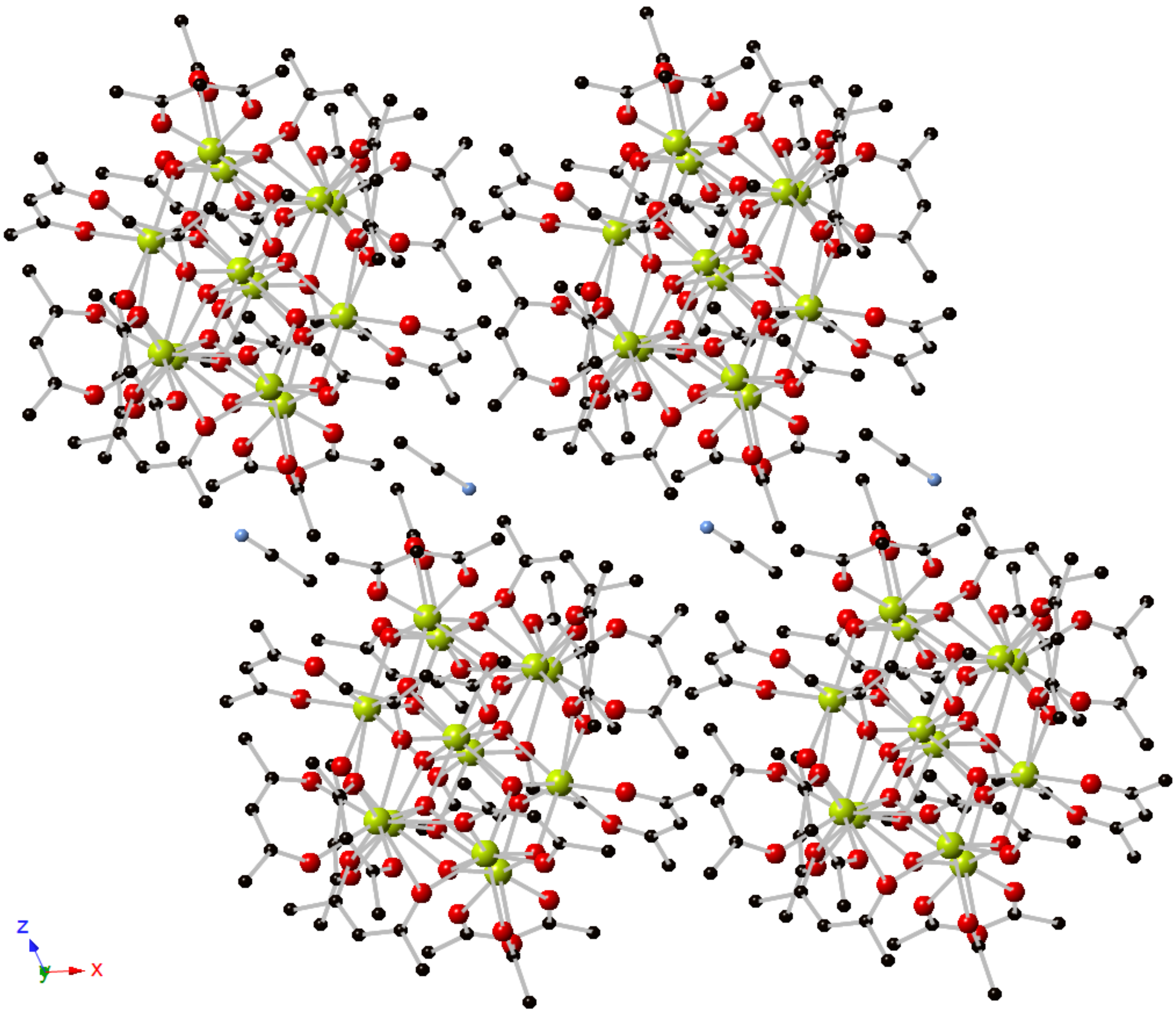

Figure S11. Packing diagram of **Ce-12** viewed down the [010] with the ACN molecules in the outer coordination sphere are shown. The Ce atoms are depicted in yellow-green, O in red, N in blue, and C in black.

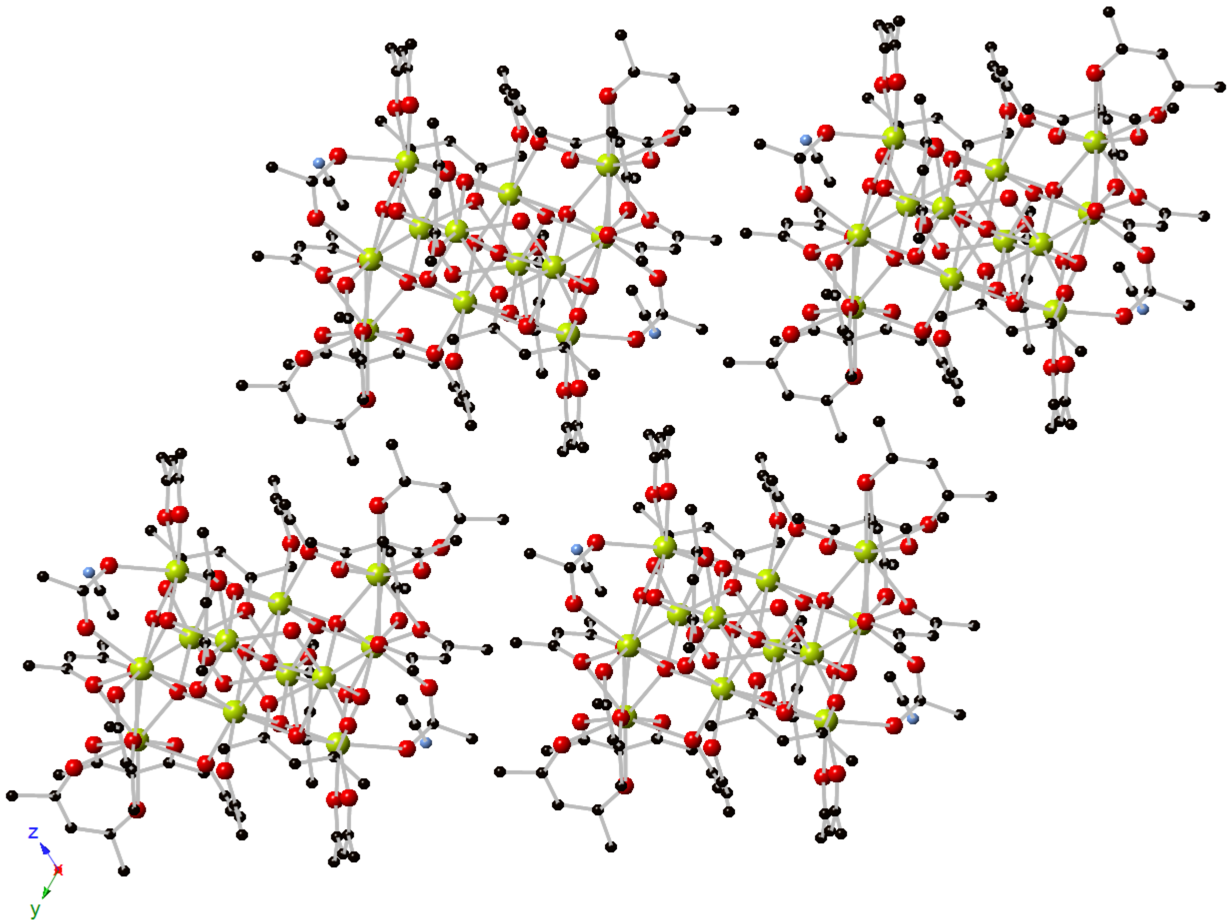

Figure S12. Packing diagram of **Ce-12** viewed down the [100] with the ACN molecules in the outer coordination sphere are shown. The Ce atoms are depicted in yellow, O in red, N in blue, and C in black.

V. DESCRIPTION OF CE COORDINATION CHEMISTRY IN CE-10 AND CE-12

Compound **Ce-10**, Ce_{10}. Five crystallographically unique Ce sites constitute the structure of **Ce-10**. The Ce^{IV} hexamer at the center of the decamer consists of Ce(1), Ce(2), Ce(4), and their symmetry equivalent sites, each of which is eight-coordinate. Ce(1) is bound to two chelating, bridging $acac^-$ ligands, two μ_3-oxo and two μ_4-oxo groups. Ce(2) is similarly bound to two $acac^-$ ligands, two μ_3-oxo, two μ_4-oxo; however, the acac are each bound through one oxygen. As such Ce(2) is also bound to one μ_3-OMe, and one terminal OMe^- group. Ce(4) is also bound to two $acac^-$ ligands; one exhibits a chelating coordination mode, while the other is monodentate, bridging. Ce(4) is further coordinated to two μ_4-oxo, two μ_3-oxo, and one μ_3-OMe group. By contrast, the

Ce^{III} sites (Ce(3) and Ce(5)) that form the methoxy bridged dinuclear structural units that cap two ends of the hexamer are nine-coordinate. Ce(3) is bound to four oxygen atoms from three $acac^-$ ligands; two are bound in a bidentate fashion and one is monodentate. Ce(3) is further coordinated to five oxygen atoms from one μ_4-oxo, two μ_3-OMe groups, and one terminal MeOH. Alternatively, Ce(5) is bound to six oxygen atoms from four $acac^-$ ligands (two chelating and two monodentate) and three oxygen atoms from two μ_3-OMe, and one μ_4-oxo.

Table S2. Average Ce-O bond distances in **Ce-10**. The oxidation state of the Ce site is clearly denoted.

**Ce-10**, Ce_{10}.						
	**CN**	**μ_4-O (Å)**	**μ_3-O (Å)**	**O_{acac} (Å)**	**O_{MeO} (Å)**	**O_{MeOH} (Å)**
**Ce^{IV}**						
Ce1	8	2.2670(5)	2.2529(5)	2.5478(11)	-	-
Ce2		2.3422(5)	2.2305(6)	2.6046(6)	2.3083(7)	-
Ce4		2.2991(5)	2.2155(6)	2.4652(10)	2.3911(6)	-
**Ce^{III}**						
Ce3	9	2.3964(5)	-	2.5423(8)	2.5558(6)	2.5371(8)
Ce5		2.5623(7)	-	2.5623(7)	2.5396(6)	-

Compound **Ce-12**, Ce_{12}. Ce1 has a coordination number of 9, which is comprised of two bidentate terminal acac ligands, three μ_2-acac ligands, and a μ_4-oxo group. Ce-O_{acac} bond distances for terminal acac and μ_2-acac range from 2.4059(4) to 2.4702(4) Å and 2.5876(4) to 2.6721(4) Å, respectively. The bond distance for the μ_4-O is 2.4673 Å, which is consistent with the longer distances seen for μ_4-O in **Ce-10**. Four of the five crystallographically unique Ce sites that make up the edge-sharing hexamers, Ce2-Ce5, are eight-coordinate while the remaining crystallographically unique Ce site, Ce6, is seven-coordinate. Ce2 is coordinated to one μ_2-O, two μ_3-O, two μ_4-O, and two acac ligands, one of which is binding in a bidentate fashion. The bond distances for Ce2-O_{acac} range from 2.3314 to 2.5044 Å, Ce-μ_2-O25 is 2.3534(4) Å, Ce2-μ_3-O19 and Ce2-μ_3-O20 are 2.2341(4) and 2.4586(4) Å, respectively, and Ce2-μ_4-O24 and Ce2-μ_4-O26 are 2.1735(4) Å and 2.3098(4) Å, respectively. Ce3 is coordinated to one acac ligand, three μ_3-O, and three μ_4-O ligands. Bond distances for Ce3-O_{acac} are 2.4585(4) and 2.4318(4) Å, Ce3-μ_3-O range from 2.2173(4) to 2.5136(4) Å, and Ce3-μ_4-O range from 2.2681(4) to 2.4276(4) Å. Ce4 is coordinated to one bidentate acac, one bridging acac, one μ_2-O, three μ_3-O, and one μ_4-O. Bond

distances for Ce4-O_{acac} range from 2.3104(4) to 2.6799(4) Å, Ce4-μ_2-O is 2.3105(4) Å, Ce4-μ_3-O range from 2.2544(4) to 2.2677(4) Å, and Ce4-μ_4-O is 2.3699(4) Å. The coordination sphere of Ce5 is comprised of one bidentate acac with one of the oxo groups forming a μ_2 bridge with Ce1, one μ_2-acac, one acetate, three μ_3-O, and one μ_4-O. The bond distances for Ce-O_{acac} range from 2.3319(4) to 2.5004(4) Å, Ce5-$O_{acetate}$ is 2.4090(4) Å, Ce5-μ_3-O range from 2.2115(4) to 2.2494(4) Å, and Ce5-μ_4-O is 2.3277(4) Å. The coordination sphere of Ce6 contains a bidentate acac, an acetate, and four μ_3-O. The bond distance for Ce6-$O_{acetate}$ is 2.4170(4) Å, for Ce6-μ_3-O the bond distance ranges from 2.1870(4) to 2.397(4) Å, and for Ce6-O_{acac}, the bond distances are 2.3132(4) and 2.3683(4) Å.

Table S3. Average Ce-O bond distances in **Ce-12**. The oxidation state of the Ce site is clearly denoted.

	**CN**	**μ_4-O (Å)**	**μ_3-O (Å)**	**μ_3-OH(Å)**	**μ_2-OH (Å)**	**O_{acac} (Å)**	**O_{Ac} (Å)**
**Ce^{IV}**							
Ce2	8	2.2417(4)	2.2341(4)	2.4586(4)	2.3534(4)	2.4179(4)	-
Ce3	8	2.3479(4)	2.2179(4)	2.5136(4)	-	2.4452(4)	-
Ce4	8	2.3699(4)	2.2612(4)	-	2.3105(4)	2.4952(4)	-
Ce5	8	2.3277(4)	2.2305(4)	-	-	2.4162(4)	2.4090(4)
Ce6	7	-	2.1992(4)	2.3979(4)	-	2.3408(4)	2.4170(4)
**Ce^{III}**							
Ce1	9	2.4673(4)	-	-	-	2.5390(4)	-

VI. POWDER X-RAY DIFFRACTION (PXRD) PATTERNS

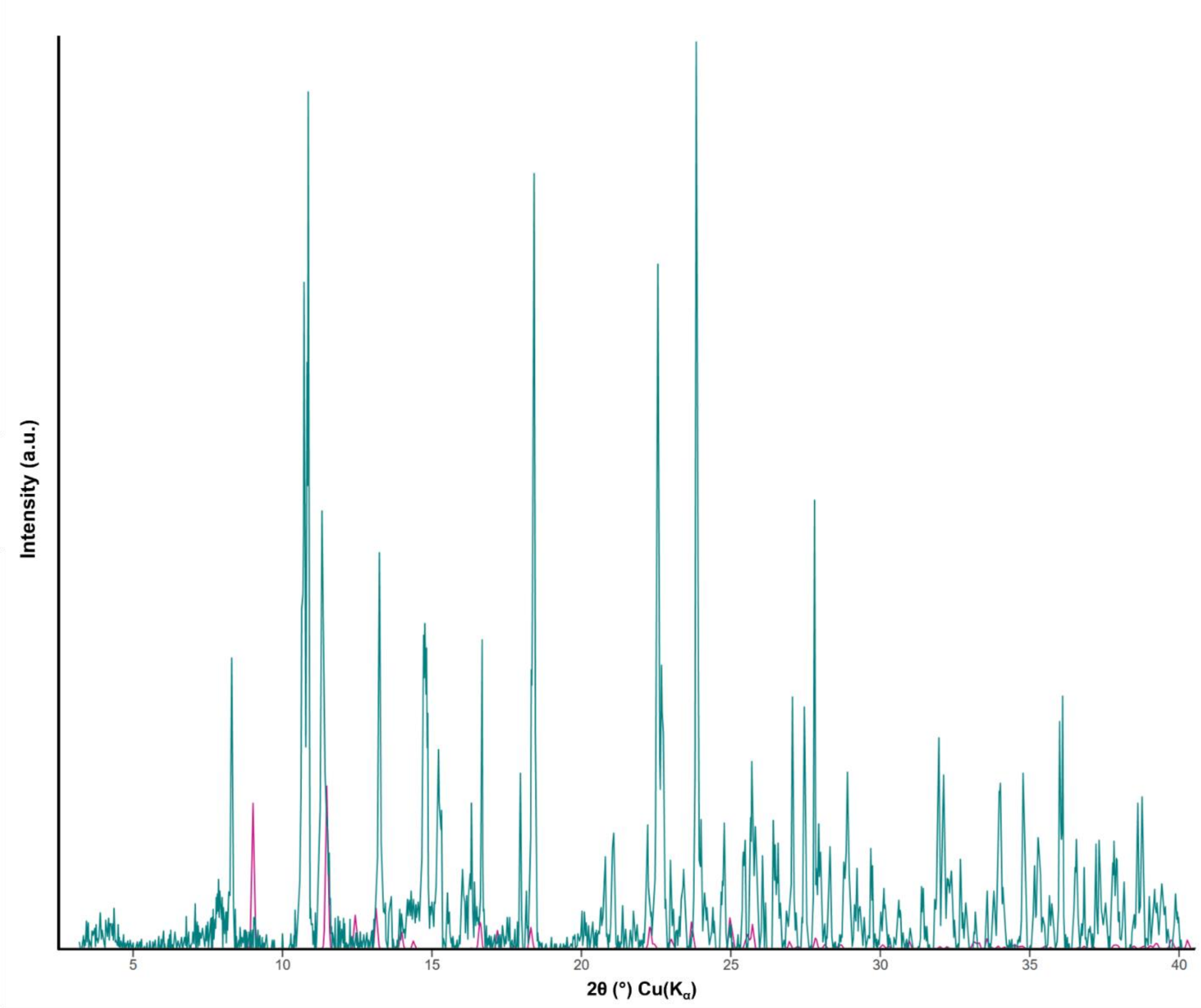

Figure S13. The pXRD pattern calculated from the single crystal data of Ce-1 (pink) overlaid with the pattern collected for the reaction product (teal). The data shows that Ce-1 is a minor phase. Efforts to identify the other phase(s) were unsuccessful.

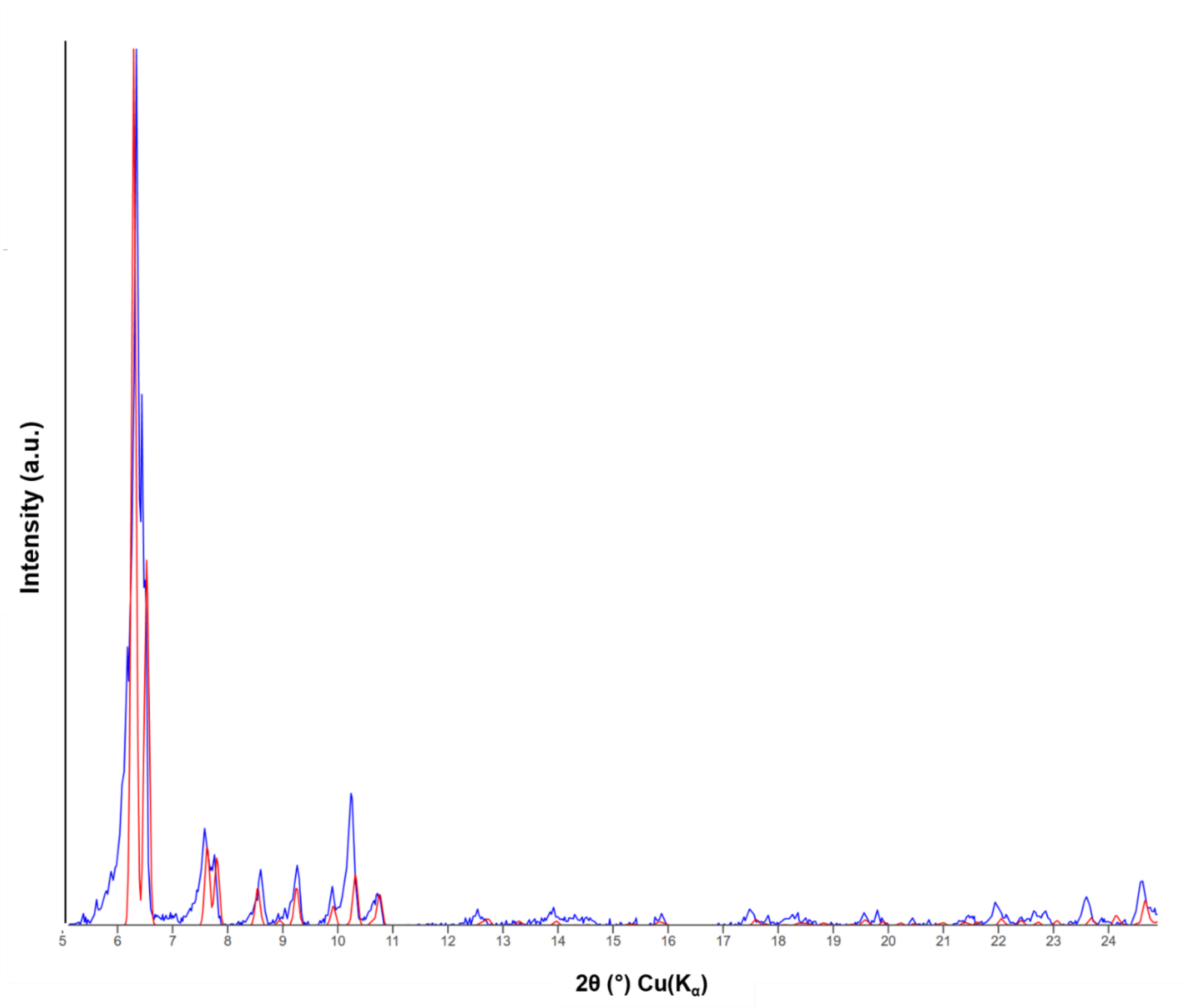

Figure S14. Calculated (red) and experimental (blue) pXRD patterns for Ce-10 shown over 5 to 25 ° 2θ. Agreement between the calculated and experimental patterns suggests that Ce-10 is the major reaction product.

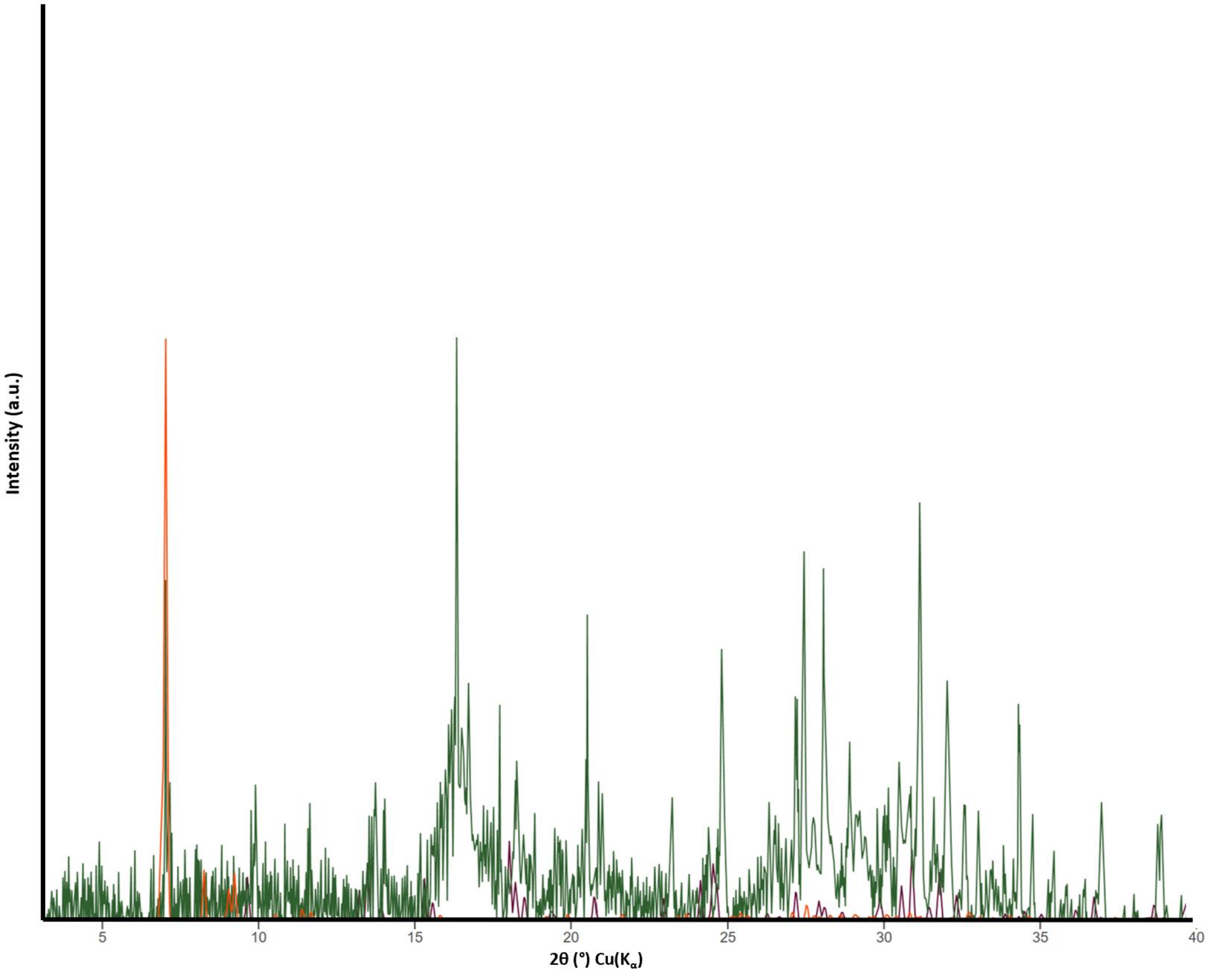

Figure S15. Calculated (Ce-10.3; orange) and experimental (green) pXRD patterns for the product obtained from $Ce_2(SO_4)_3$ shown over 5 to 40° 2θ. The powder pattern shows that Ce-10.3 (orange) is present as a minor phase. The other phases were not identified.

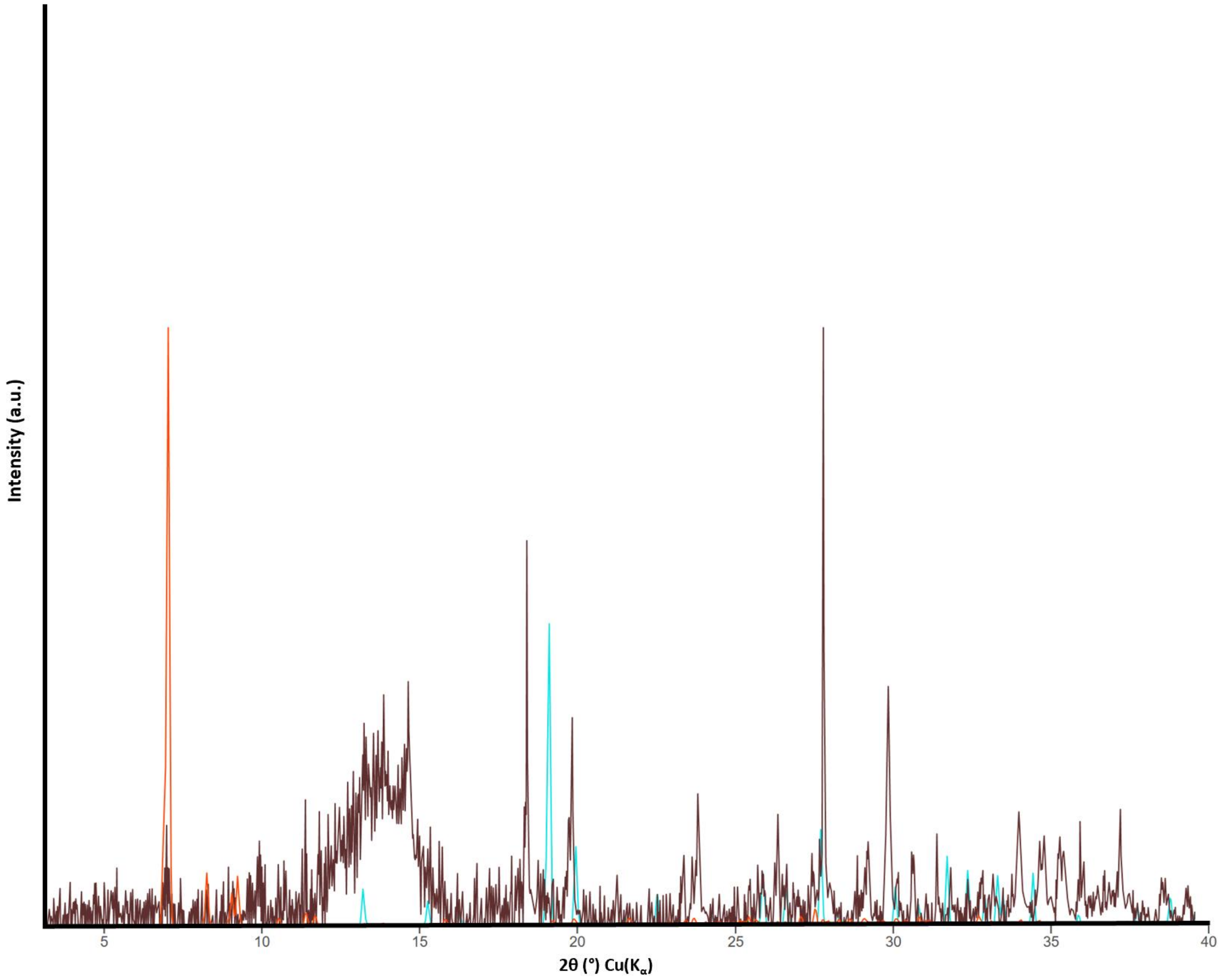

Figure S16. Calculated (**Ce-10.2**; orange) and experimental (brown) pXRD patterns for the product obtained from $Ce(SO_4)_2$ shown over 5 to 40° 2θ. **Ce-10.2** is present as a minor phase. Peaks consistent with $Ce(SO_4)_2$ (light blue) are observed; however, efforts to identify the other reaction products were unsuccessful.

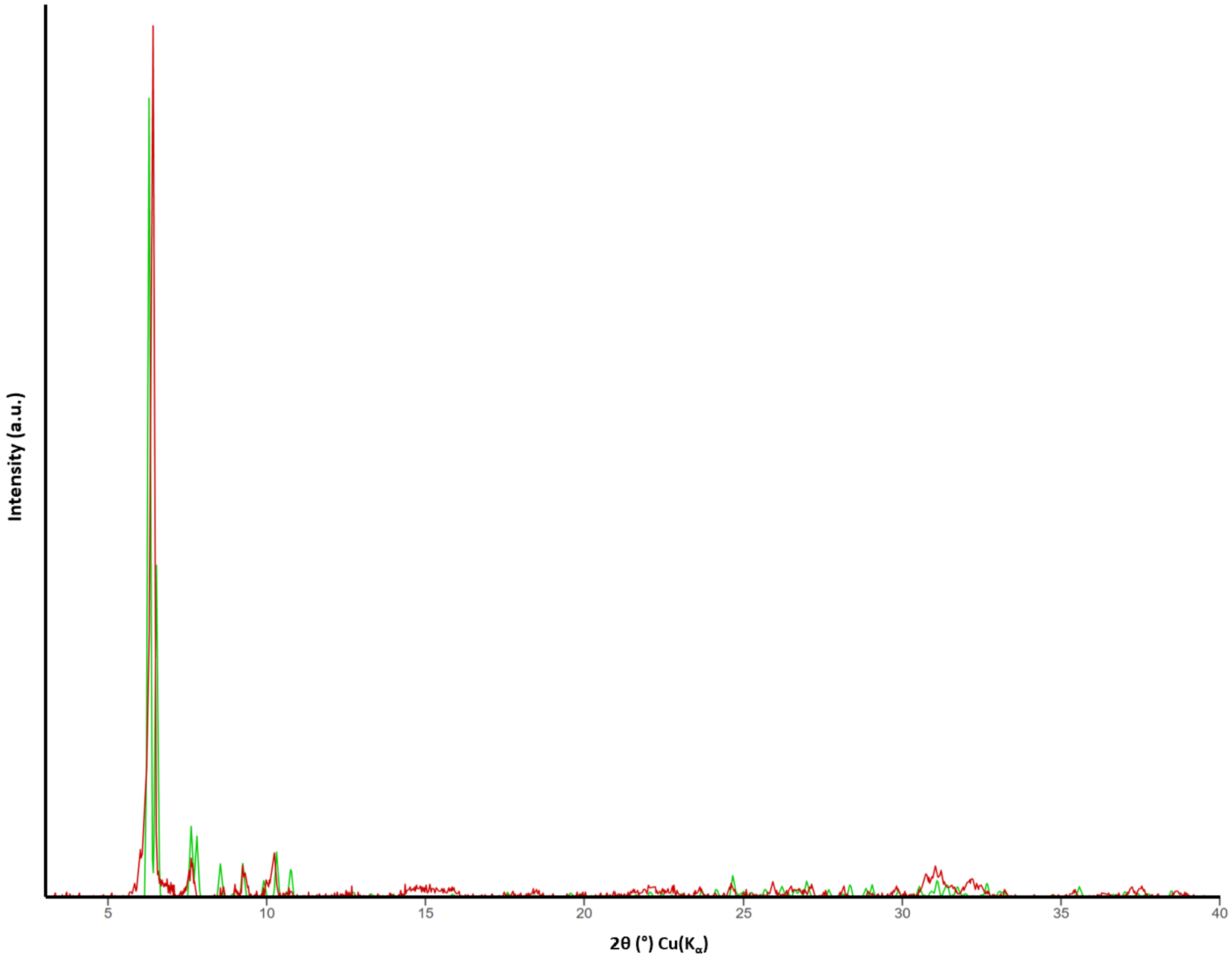

Figure S17. Calculated (**Ce-10**, green) and experimental (red) pXRD patterns for the product obtained from Ce(triflate)$_3$ shown over 5 to 40° 2θ. **Ce-10** is identified as the major phase.

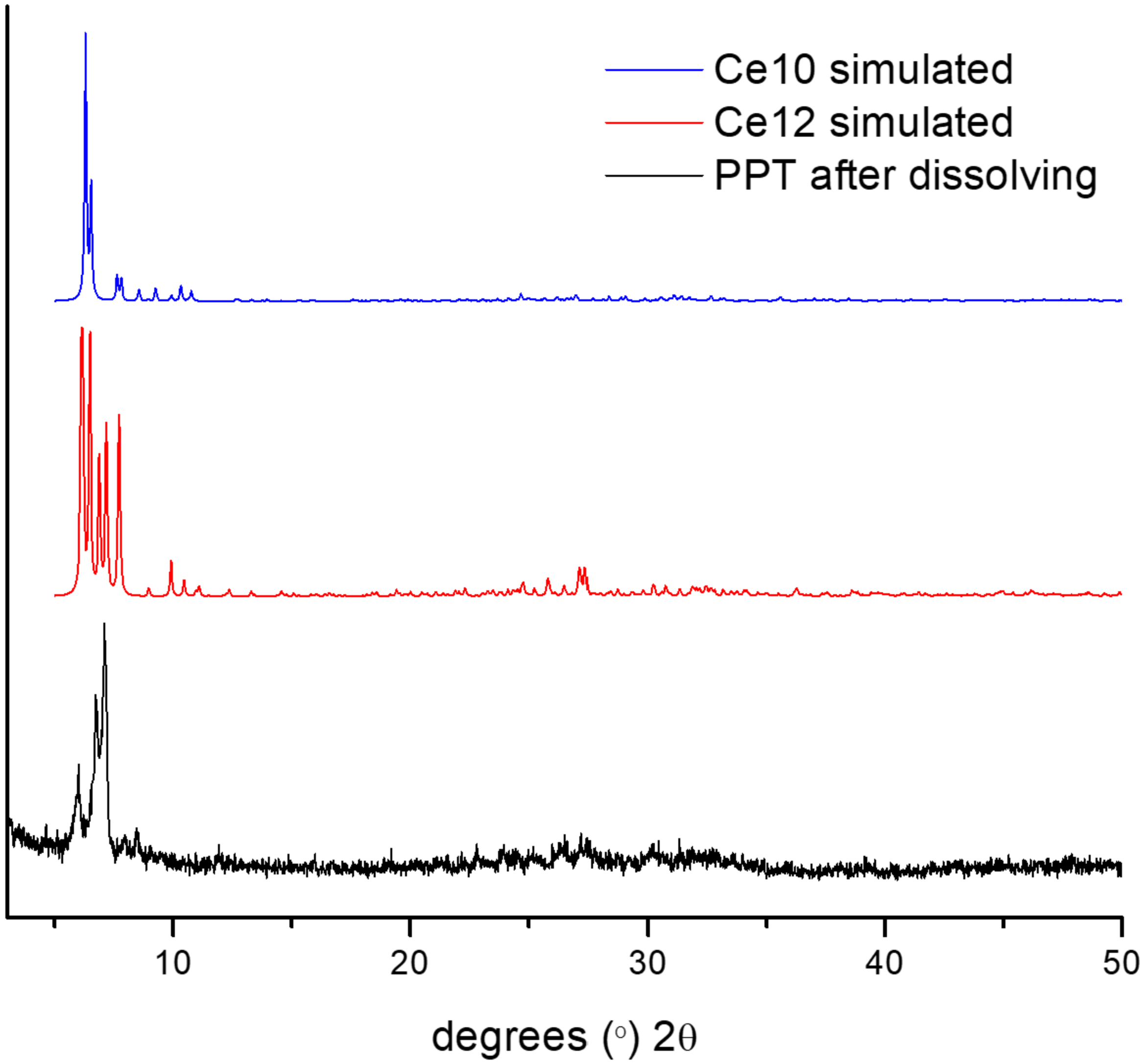

Figure S18. Calculated pXRD patterns for the Ce-10 (blue) and Ce-12 (red) and experimental pXRD pattern collected on the precipitate that forms from a solution of Ce-10 in ACN (black).

VII. SMALL-ANGLE X-RAY SCATTERING (SAXS)

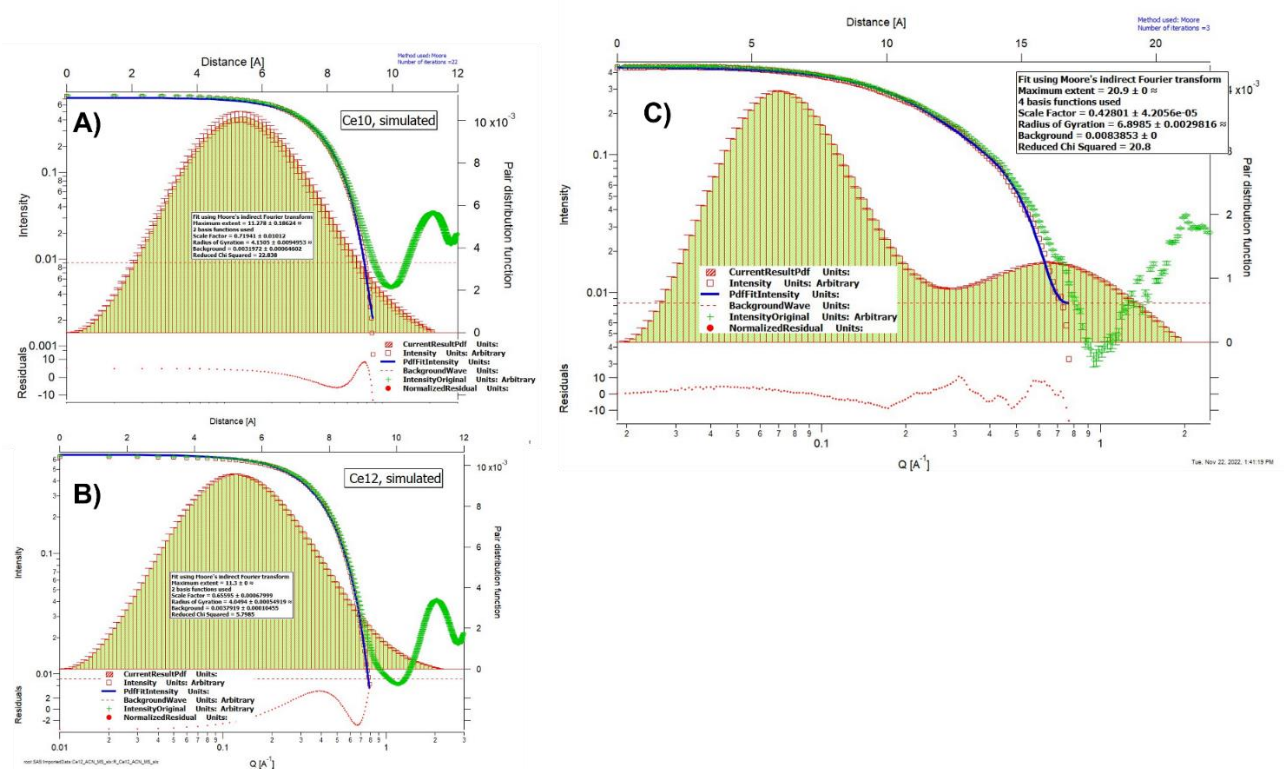

Figure S19. Pair distribution distance analysis (PDDF) of scattering data for simulated Ce_{10} **(A)**, simulated Ce_{12} **(B)**, and experimental Ce-10 dissolved in acetonitrile **(C)**.

VIII. INFRARED (IR) SPECTRA

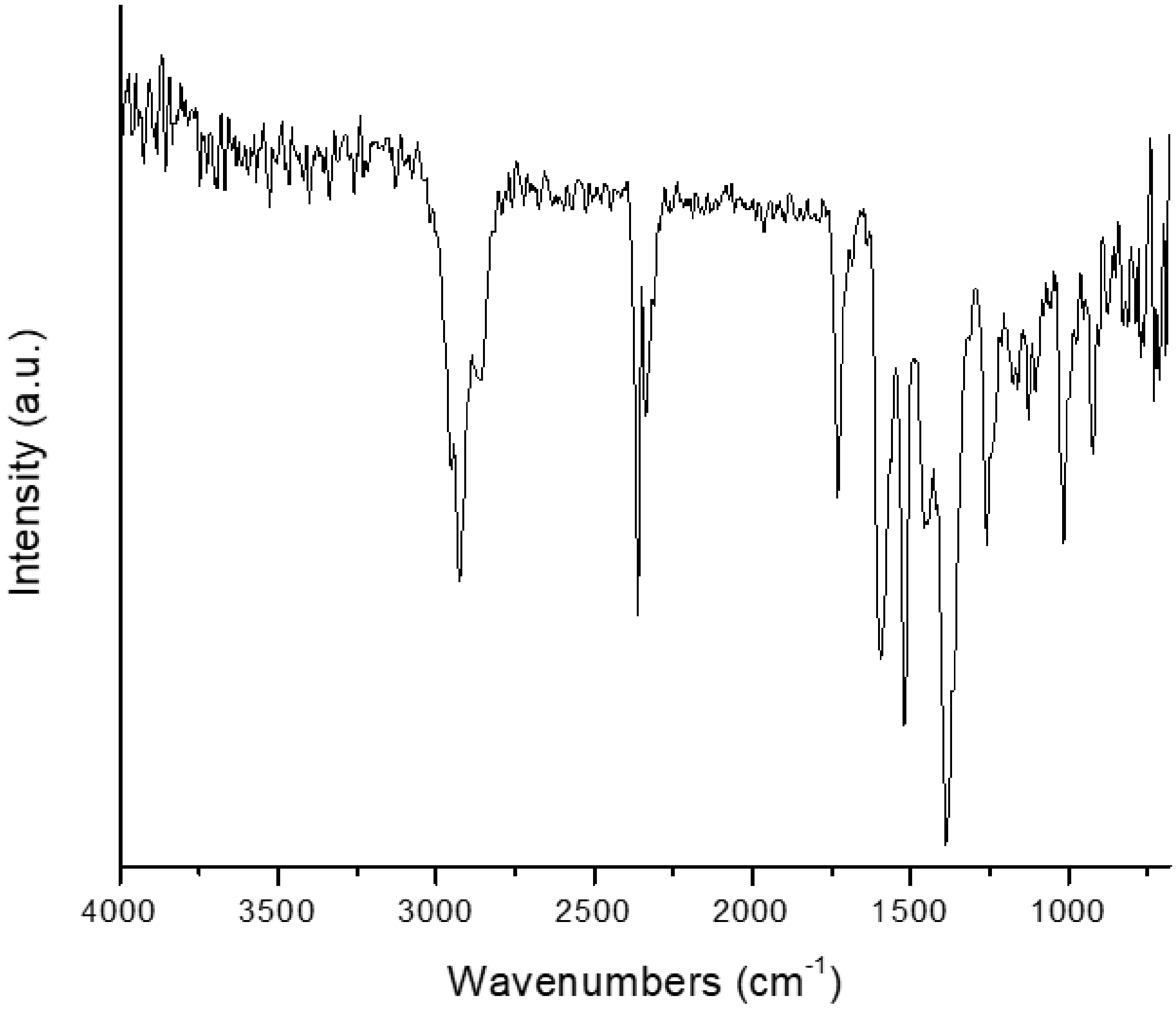

Figure S20. The IR spectrum for compound **Ce-10** obtained using a Nicolet iN10 IR Microscope with a spectral window from 675 – 4000 cm^{-1}. A list of the peaks with their assignment are provided in Table S1.

Table S4. IR peak positions and assignment for **Ce-10**.

IR (cm^{-1})	Assignment
921	ν(C⋯C) + ν(C⋯O)[1]$_{acac}$
1014	$\rho_r(CH_3)$[1]$_{acac}$
1126	
1257	acac[2]; C-C stretch + C-CH_3 stretch[3]
1388	$\delta_d(CH_3)$[1]$_{acac}$
1454	δ(CH) + ν(C⋯C)[1]$_{acac}$
1519	C=O stretch + CH bend[3]$_{acac}$
1592	C=C stretch [3]$_{acac}$
1681	
1731	acac[2]
2337	CO_2
2360	CO_2/acac[2]
2854	
2927	$\nu(CH_3)$ acac [1]
2954	$\nu(CH_3)$ acac[1]

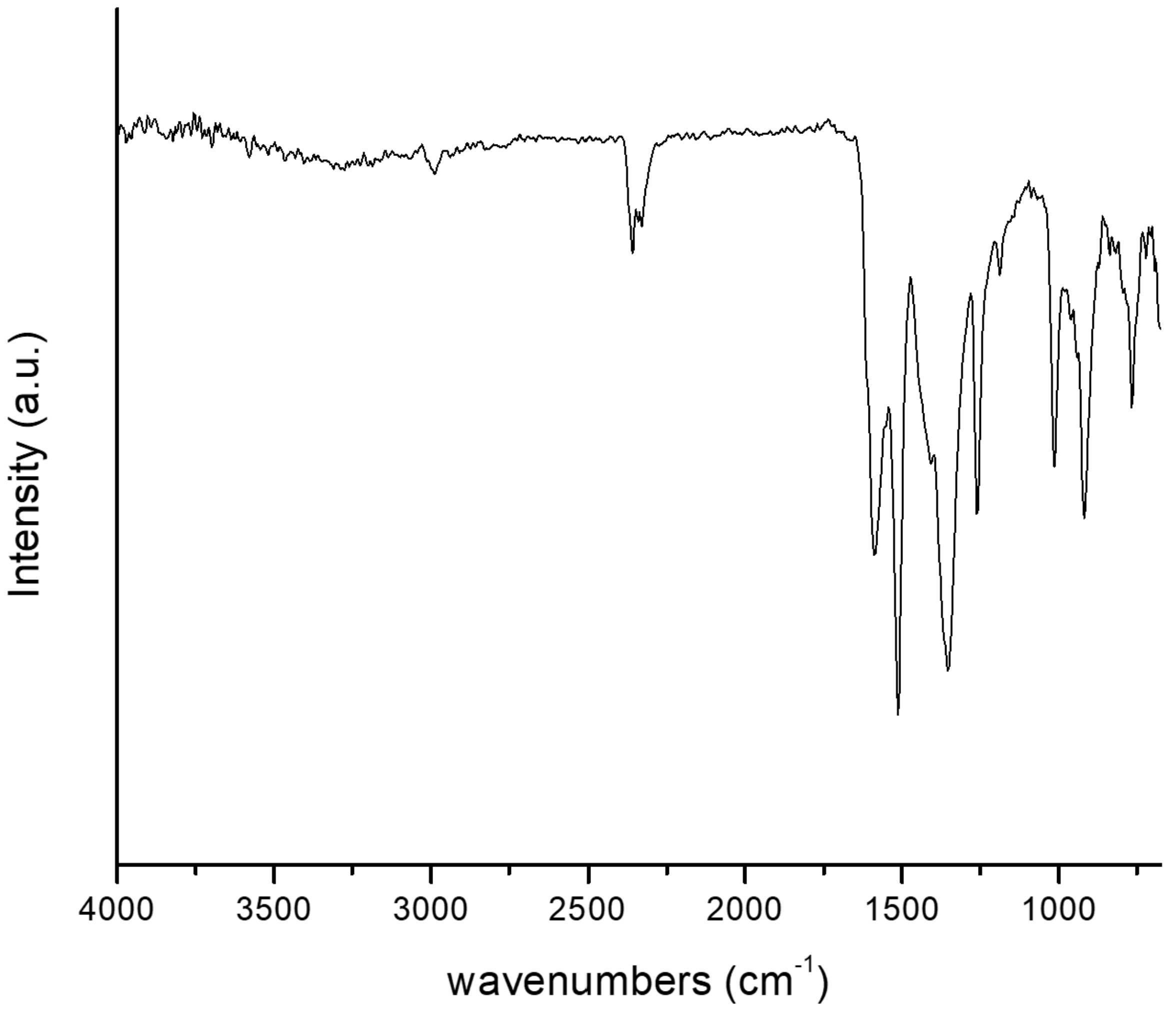

Figure S21. The IR spectrum for compound **Ce-12** obtained using a Nicolet iN10 IR Microscope with a spectral window from 675 – 4000 cm^{-1}. A list of the peaks with their assignment are provided in Table S2.

Table S5. IR peak positions and assignments for **Ce-12**.

IR (cm^{-1})	Assignment
767	$\pi(CH)_{acac}$
917	
960	
1014	$\rho_r(CH_3)$[1]$_{acac}$
1187	
1261	acac[2]; C-C stretch + C-CH_3 stretch[3]
1353	$\delta_s(CH_3)_{acac}$
1407	$\delta_{as}(CH_3)$, $\nu_s(CO)_{CH3COO}$[4]
1511	C=O stretch + CH bend[3]$_{acac}$

1589	C=C stretch [3]$_{acac}$
2360	CO_2/acac[2]
2989	$\nu(CH_3)_{acac}$

IX. ¹H NMR SPECTRUM OF CE-10

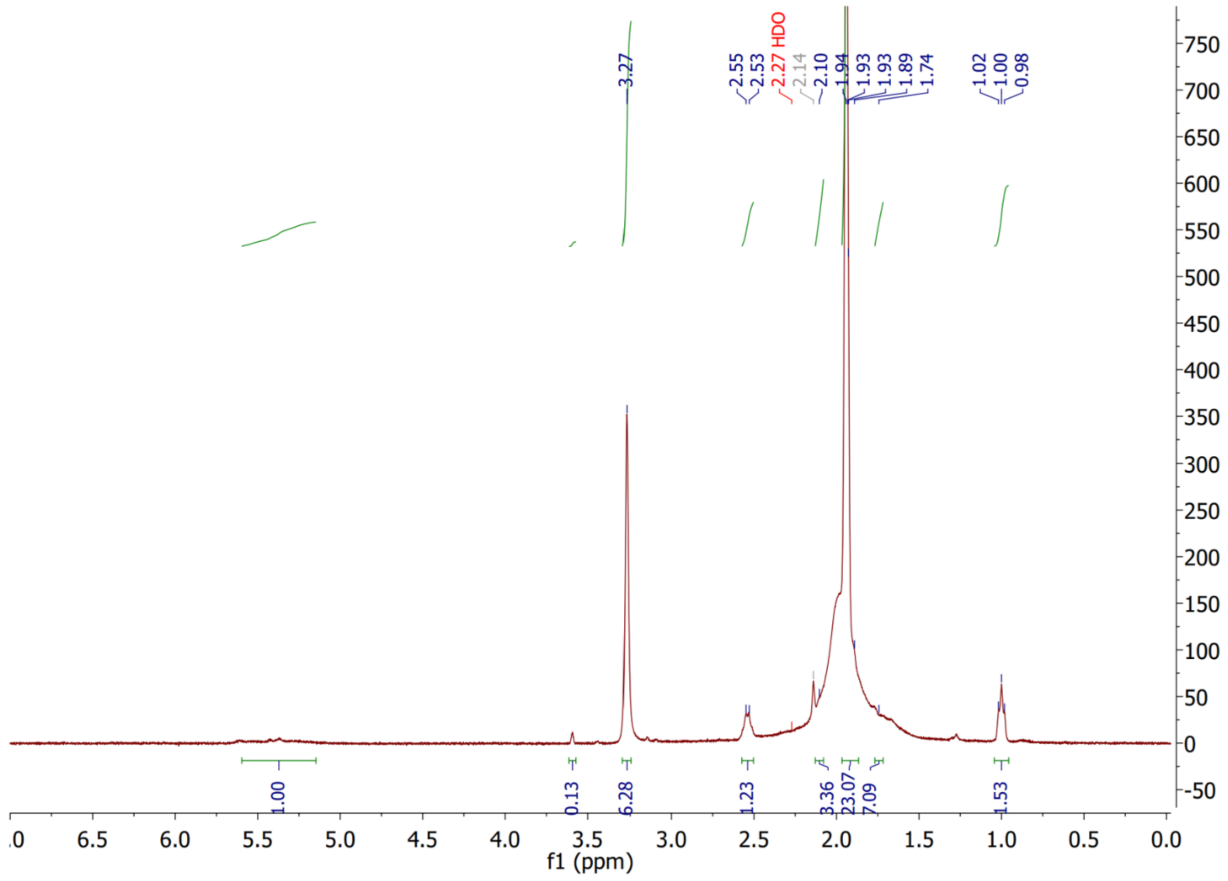

Figure S22. ¹H NMR spectrum of **Ce-10** in CD_3CN. The solvent peak is displayed as a sharp peak at 1.94 ppm.

Table S6. ^{1}H NMR peaks and their assignments for **Ce-10** dissolved in deuterated acetonitrile.

Peak position (ppm)	Assignment
1.93	acetate
2.10	MeOH
3.27	MeOH
3.6	C<u>H</u>$_2$ free Hacac[5]
5.45	C<u>H</u> bound acac^{1-} [5]
5.6	C<u>H</u> enolate form Hacac[5]

Peak assingment for ^{1}H NMR showed the presence of free acetylacetone just over 3.5 ppm and the enolate form of acetylacetone in the region between 5.45 to 5.6 ppm.[5] Evidence of MeOH is also present in the spectrum, with peaks at 2.10 ppm (CH) and 3.27 ppm (CH_3). There is also an acetate peak that is displayed at 1.93 ppm.

X. BVS FOR COMPOUNDS CE-10, CE-10.2, CE-10.3 AND CE-12

Table S7. BVS values and assignment for Ce and O atoms in {Ce_{10}}, **Ce-10**. BVS values are consistent with six Ce(IV) and four Ce(III) sites in the cluster core.

Atom	BVS value	assignment
**Ce1**	3.94	Ce^{IV}
**Ce2**	4.10	Ce^{IV}
**Ce3**	2.63	Ce^{III}
**Ce4**	4.21	Ce^{IV}
**Ce5**	2.60	Ce^{III}
**O16**	2.07	O^{2-}
**O17**	2.15	O^{2-}
**O18**	2.06	O^{2-}
**O19**	2.12	O^{2-}

Table S8. BVS values and assignment for Ce and O atoms in {Ce_{10}}, **Ce-10.2**. BVS values are consistent with six Ce(IV) and four Ce(III) sites in the cluster core.

Atom	BVS value	Assignment
**Ce1**	2.73	Ce^{III}
**Ce2**	3.73	Ce^{IV}
**Ce3**	4.05	Ce^{IV}
**Ce4**	3.98	Ce^{IV}

**Ce5**	2.64	Ce^{III}
**O1**	2.16	O^{2-}
**O2**	2.07	O^{2-}
**O3**	2.05	O^{2-}
**O4**	2.13	O^{2-}

Table S9. BVS values and assignment for Ce and O atoms in $\{Ce_{10}\}$, **Ce-10.3**. BVS values are consistent with six Ce(IV) and four Ce(III) sites in the cluster core.

Atom	BVS value	Assignment
**Ce1**	2.61	Ce^{III}
**Ce2**	3.96	Ce^{IV}
**Ce3**	3.75	Ce^{IV}
**Ce4**	4.21	Ce^{IV}
**Ce5**	2.70	Ce^{III}
**O8**	2.13	O^{2-}
**O10**	2.08	O^{2-}
**O11**	2.07	O^{2-}
**O14**	2.14	O^{2-}

Table S10. BVS values and assignment for Ce and O atoms in $\{Ce_{12}\}$, **Ce-12**. BVS values are consistent with ten Ce(IV) and two Ce(III) sites in the cluster core.

Atom	BVS value	Assignment
**Ce1**	2.66	Ce^{III}
**Ce2**	3.99	Ce^{IV}
**Ce3**	3.91	Ce^{IV}
**Ce4**	3.88	Ce^{IV}
**Ce5**	4.01	Ce^{IV}
**Ce6**	3.95	Ce^{IV}
**O19**	2.09	O^{2-}
**O20**	1.14	OH^{-}
**O21**	2.14	O^{2-}
**O22**	2.09	O^{2-}
**O23**	2.05	O^{2-}
**O24**	2.20	O^{2-}
**O25**	1.06	OH^{-}
**O26**	2.08	O^{2-}

XI. X-RAY ABSORPTION SPECTROSCOPY (XAS) SAMPLE PREPARATION

General

Boron nitride [BN, Alfa Aesar], ceria (CeO_2, Acros Organics), and cerium(III) acetylacetonate hydrate [$Ce(acac)_3\bullet(H_2O)_x$, MilliporeSigma] were obtained commercially and used as received. The cerium cluster, [$Ce_{10}O_8(acac)_{14}(CH_3O)_6(CH_3OH)_2$], was prepared at Georgetown University and shipped to LANL for characterization by X-ray absorption spectroscopy (XAS).

Sample Preparation

The Ce L_3-edge XAS samples were prepared on the benchtop at Los Alamos National Laboratory (LANL) with no attempt to exclude air and moisture. Each sample (1.8 mg for CeO_2, 2 mg for $Ce_{10}O_8(acac)_{14}(CH_3O)_6(CH_3OH)_2$ and 4.78 mg for $Ce(acac)_3\bullet(H_2O)_x$) was mixed with BN (~36 mg) and ground by hand with a mortar and pestle (10 min). We observed that this ratio of sample to BN provided a balance between generating reasonable signal, an edge jump near 1 (pre-normalization edge jump: ~0.5) and minimal self-absorption. Meanwhile, the back side of a slotted XAS sample holder was equipped with Kapton tape (0.5 mil). The holder was 18 mm tall, 25 mm wide, and 1 mm thick. The slot was 4×20 mm. The aforementioned finely ground analyte and BN mixture was loaded into a slotted aluminum sample holder using a spatula. The front side of the holder was sealed with another layer of Kapton tape (0.5 mil). Holders were then loaded into LANL's "in-house" XAS spectrometer, which has been affectionally named the "Ditter-A-Tron."

XII. X-RAY ABSORPTION SPECTROSCOPY (XAS) INSTRUMENT CONFIGURATION AND DATA ANALYSIS

Instrument configuration

Ce L_3-edge XAS data were collected on an in-house XAS spectrometer at Los Alamos National Laboratory. At a high level, the instrument has a high-power X-ray source (Varex 50 W Pd X-ray

tube), an Amptek X-123 Si-PIN diode detector, and a spherically bent crystal analyzer (Si 4,2,2) affixed to linear translation stages and “steering bars.” The motorized translation stage enabled alignment between the source, detector, and crystal analyzer to be rigorously maintained as the source and detector positions (and hence Bragg angle) were scanned along a 1-m Rowland circle. The geometry enabled access to Bragg angles between 55º and 85º. The source was masked with lead tape that contained a cylindrical slit size (diameter ~3 mm). The detector was equipped with a 3D printed slit (12 x 6.5 mm), to which the sample was affixed with electrical tape. A helium filled box (equipped with Kapton windows) was placed in the flight path between the source and crystal analyzer as well as between the crystal analyzer and the detector.

The spectrometer was configured for Ce L_3-edge XAS as follows. First the crystal analyzer was oriented to maximize signal on the appropriate crystal reflection (ROI = 251 to 283 on this instrument). To achieve this, the crystal was rotated around the incoming beam axis by ~20 cm, and the count rate on the detector at a given energy for a short exposure (10 sec) was determined. This procedure was repeated until the orientation that maximized detected count rates was obtained. The detector was then windowed on the appropriate crystal reflection for Ce measurements (the first reflection, E^0 = 5591.9 eV) using a Cr metal foil (which has a similar absorption edge to Ce). The offset for the detector was then determined by setting the position/angle of the detector and source to 5700 eV (78.82º). The detector was then scanned (+/- 0.4º) over the count rate peak maximum. The detector offset (detector offset = detector angle – source angle) was determined to be -0.3 ° by choosing the energy position with the greatest number of counts and subtracting that value from the original position. A dark spectrum (1 scan) was obtained for the excitation beam (I0; sample removed from the spectrometer) and for the I_1 (sample in place within the spectrometer) for each sample by setting the detector offset to 0.0 and collecting a spectrum. Note, setting the offset to zero pushes the beam off the detector and enables contributions to the data from unwanted X-ray scatter and electronic noise to be quantified. The darks were subtracted from the data during processing. The incident beam intensity (I_0) was determined by collecting 1 dark current scan using the -0.3° offset and 50 background scans with the sample removed from the spectrometer. The sample was fixed between the detector and the crystal analyzer. A single X-ray energy was selected to shine on the sample by rigorously controlling the source–crystal analyzer–detector angle. When the sample was not in the beam, typical count rates were between 2500 and 1500 (5623 to 6023 eV). That rate decreased to be on the order of 1200 to 400 counts when the samples were placed

in the beam path. The Ce L_3-edge XAS data were collected in transmission mode. Fifty scans were obtained and averaged per cerium sample and four scans were obtained and averaged for the Cr foil (used for energy calibration).

Data Analysis

Data manipulation and analysis were conducted as previously described by Solomon and co-workers.[6] The Ce L_3-edge XAS data were calibrated in energy to the first inflection point of the Cr K-edge from a metallic chromium foil (5989 eV), measured ex situ. Intensity from dark current measurements was subtracted from the sample signal. Then, the sample transmission intensity was ratioed against I_0 as $-ln\frac{I_0}{I_1}$; where I_0 was the background spectrum (collected ex situ, see above) and I_1 was the transmission signal associated with the sample. Using the Athena program, the data were analyzed by fitting a line to the pre-edge region (5692 eV to 5709 eV), which was subsequently subtracted from the experimental data to eliminate the background of the spectrum. Treating the data this way normalized each spectrum to a single Ce atom. Data were normalized by fitting a third order polynomial post-edge region of the spectrum (~5765 to 5910 eV, sample dependent) and setting the edge jump at 5725.7 eV to an intensity of 1.0. Linear combination analysis was also performed using the Athena program from 5705 to 5755 eV. A deconvolution model for the Ce L_3-edge XAS data was obtained using a modified version of EDG_FIT[7] within the IGOR 8.02 platform. Approximate peak positions were determined using first and second derivatives of each spectrum. Absorption peaks within the pre-edge and edge regions (~5719 to 5737 eV) were modeled using symmetry-constrained Gaussian functions and the absorption threshold was modeled with a function consisting of a 1:1 ratio of an arctangent function and an error function. The energy position for the step function was optimized near the values used previously[8] and then constrained for the final fit for both CeO_2 and **Ce-10** . Deconvolution of spectra was obtained over several energy ranges.

XIII. ELECTROSPRAY IONIZATION MASS SPECTROMETRY

To find potential molecular formulas, a combinatorial search was conducted using constraints of cerium nuclearity of 5-15, acac ligands of 7-30, OH^- ligands 0-20, O^{2-} ligands 0-20, MeO^- ligands 0-20. Only formulas that satisfied ion charge (based on a combination of Ce(III) and Ce(IV)) and

negative charges from ligands) and were within 30 ppm of the experimental monoisotopic mass were retained. For the triply charged ion with monoisotopic peak at m/z 948.8753, the constraints above resulted in 14 formulas among which 3 were Ce-11 and 11 were Ce-10., indicating more likelihood of Ce-10 clusters. Further filtering the list using a constrain of 6 Ce(IV) centers based on crystallographic data resulted in 4 formulas (shown in Table S11) all of which were Ce-10 clusters. Moreover, Ce-11 clusters in the original list of 14 had poorer isotopic matching with the experimental data compared to that of Ce-10 clusters.

Table S11: Table of potential Ce_{10} formulas for the major triply charged ions.

Formula for the major triply charged ion	Theoretical m/z	Difference from experimental m/z (ppm)
$Ce_{10}(CH_3COCHCOCH_3)_{13}O_{10}^{3+}$	948.8606	-15
$Ce_{10}(CH_3COCHCOCH_3)_{12}(OH)_5O_7(CH_3O)_2^{3+}$	948.8676	-8
$Ce_{10}(CH_3COCHCOCH_3)_{11}(OH)_{10}O_4(CH_3O)_4^{3+}$	948.8746	-0.7
$Ce_{10}(CH_3COCHCOCH_3)_{10}(OH)_{15}O(CH_3O)_6^{3+}$	948.8817	7

Ions in relation to **Core Ce-10 (3+)**:

The groups of doubly and singly charged ions in **Figure 5** relate to the triply charged ion and can be rationalized by variations in number of ligands. **Figures S23-S25** show the isotopic envelope matching in these ion groups using $Ce_{10}(CH_3COCHCOCH_3)_{12}(OH)_5O_7(CH_3O)_2^{3+}$ as an example formula (denoted as Core Ce-10 (3+)) for the triply charged ion. **Figure S23** depicts the main isotopic envelope of the doubly charged ion group. This envelope is explained by contributions from two main species. The major contributor is attributed to **Core Ce-10A (2+)** (red), which is **Core Ce-10 (3+)** + 1 acac + 1 OH + 1 CH_3O – 1 O (i.e. the conversion of one oxo to hydroxo and addition of a methoxo and acac). The minor contributor is attributed to **Core Ce-10B (2+)** (green), which is **Core Ce-10 (3+)** + 1 acac + 4 OH – 2 O (i.e. conversion of two oxo to two hydroxo by addition of two water molecules and addition of one acac). The second tallest isotopic envelope of the doubly charged ion group is shown in **Figure S24** and is attributed to **Core Ce-10C (2+)**, which is **Core Ce-10 (3+)** + 2 acac + 1 OH + 1 CH_3O - O + Na (i.e. conversion of one oxo to hydroxo and addition of methoxo and one acac, and possibly a non-covalent adduction of Na(acac)). **Figure S25** shows the isotopic envelope of the singly charged ion group and is attributed to **Core Ce-10 (1+)**, which is **Core Ce-10 (3+)** + 2 acac + 1 OH + 1 CH_3O – 1 O (i.e. the conversion of one oxo to hydroxo and addition of a methoxo and two acac ligands).

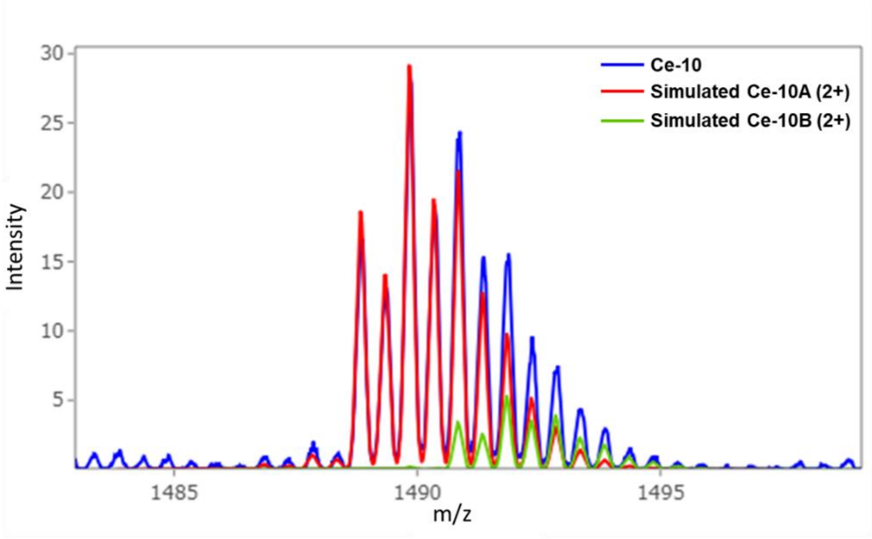

Figure S23: Experimental data of Ce-10 solution (blue) and simulated data of **Core Ce-10A (2+)** (red) and **Core Ce-10B (2+)** (green) at m/z 1488.84 and 1490.84.

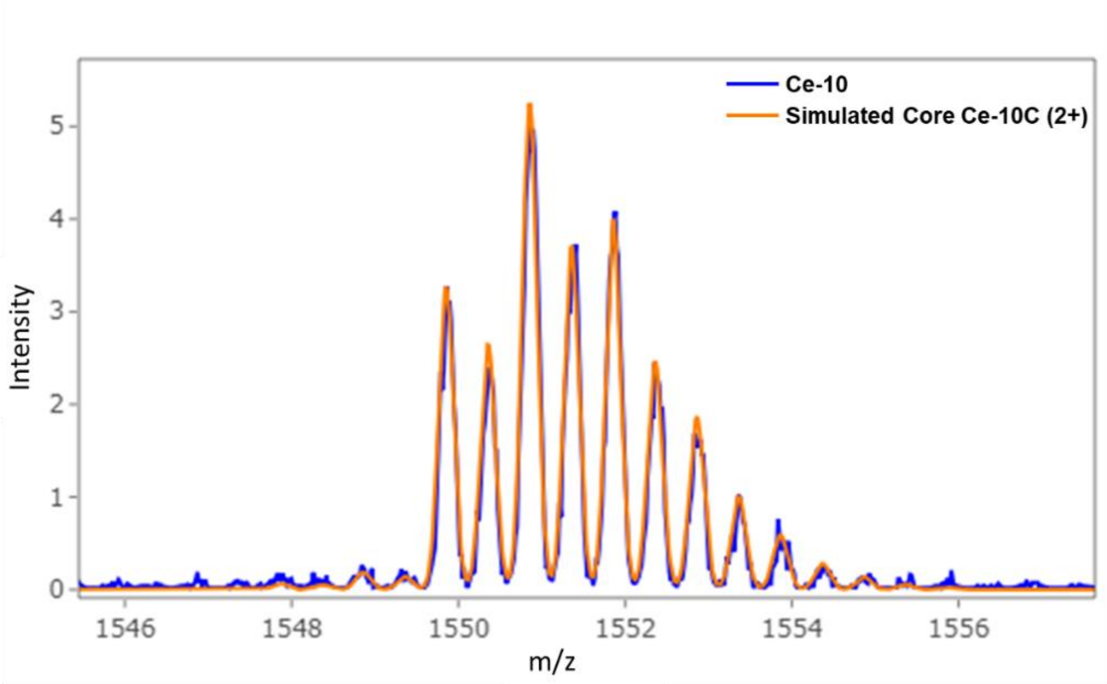

Figure S24: Experimental data of Ce-10 solution (blue) and simulated data of **Core Ce-10C (2+)** (orange) at m/z 1549.85.

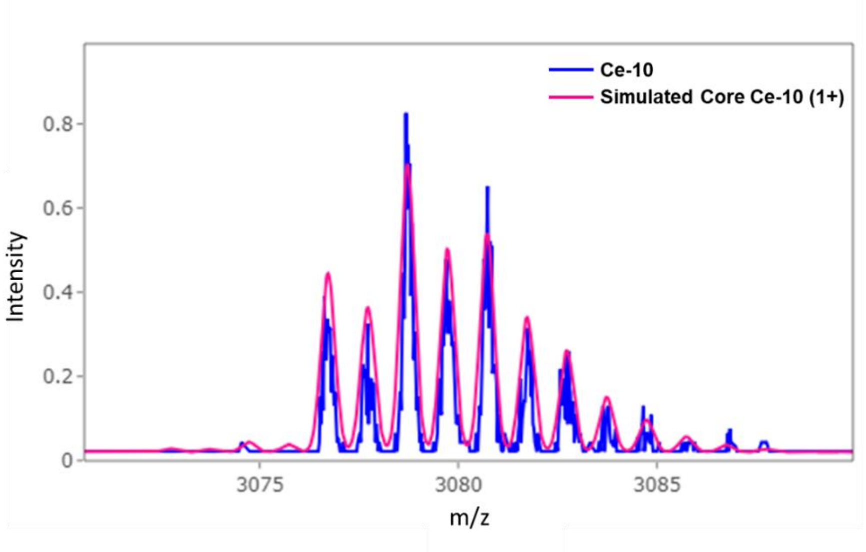

Figure S25: Experimental data of Ce-10 solution (blue) and simulated data of **Core Ce-10 (1+)** (pink) at m/z 3076.72.

XIV. HOMOMETALLIC CE-OXO CLUSTERS

Previously reported homometallic Ce clusters span from nuclearities of Ce_3 to Ce_{100}. The majority of these clusters are hexamers, with the Ce_6 core being present in higher nuclearity clusters as well. Herein reported is a table of homometallic Ce clusters with their cluster core and decorating ligands.

Table S12. Compound formula, cluster core, capping ligand, and references for previously reported Ce clusters.

Compound formula	Cluster core	Decorating ligands	reference
	**{Ce_3}**		

**$[Ce_3(\mu_3\text{-}O\mathit{t}Bu)_3(\mu\text{-}O\mathit{t}Bu)_3(O\mathit{t}Bu)\text{-}(HO\mathit{t}Bu)_2]$**	$\{Ce_3(\mu_3\text{-}O\mathit{t}Bu)_3(\mu\text{-}O\mathit{t}Bu)_3\}$	$^tBuO^-$, HO*t*Bu	9
**$[Ce_3(\mu_3\text{-}OH)_2(\mu_2\text{-}C_6H_{13}O)_3(C_6H_{13}O)_6]$**	$\{Ce_3(OH)_2(C_6H_{13}O)_3\}$	$C_6H_{13}O$	10
**$[Ce_3(OH)(NO_3)_4(LH_2)_2]\cdot 4.5dmf$**	$\{Ce_3(OH)\}$	NO_3^{2-}, LH	11
**$(pyH)[Ce_3O(NO_3)_2(LH)_2]\cdot xH_2O\cdot ydmso\cdot 2MeCN\cdot 1.5py\cdot MeOH$**	$\{Ce_3O\}$	NO_3^{2-}, LH	
**$[Ce_3(\mu_3\text{-}O)(\mu_3\text{-}OBu^t)(\mu_2\text{-}ONep)_3(ONep)_6]$**	$\{Ce_3(\mu_3\text{-}O)(\mu_3\text{-}OBu^t)(\mu_2\text{-}ONep)_3\}$	ONep	12
**$[Ce_3O(O^tBu)_{10}]$**	$\{Ce_3O(\mu_2\text{-}O^tBu)_3(\mu_3\text{-}O^tBu)\}$	O^tBu^-	13
**$[Ce_3(OCH_2{}^tBu)_9(NO_3)_2]$**	$\{Ce_3(OCH_2{}^tBu)_5\}$	NO_3^{2-}, $OCH_2{}^tBu$	14
**$[Ce_3(\mu_3\text{-}O^tBu)_2(\mu\text{-}O^tBu)_3(O^tBu)_6]$**	$\{Ce_3(\mu_3\text{-}O^tBu)_2(\mu\text{-}O^tBu)_3\}$	O^tBu^-	15
**$[Ce(O^iPr)_4]_3$**	$\{Ce_3(\mu_3\text{-}O^iPr)_2(\mu_2\text{-}O^iPr)_3\}$	O^iPr	16
**$\{Ce_4\}$**			
**$[Ce(\mu\text{-}ONep)_2(ONep)]_4$**	$\{Ce_4(ONep)_8\}$	$ONep^-$	9
**$[Ce_4(\mu_3\text{-}O\mathit{t}Bu)_3(\mu\text{-}O\mathit{t}Bu)_4(O\mathit{t}Bu)_5]$**	$\{Ce_4(\mu_3\text{-}O\mathit{t}Bu)_3(\mu\text{-}O\mathit{t}Bu)_4\}$	$^tBuO^-$	
**$[Ce_4(O_2CN^iPr_2)_{12}]$**	$\{Ce_4(\mu_2\text{-}O_2CN^iPr_2)_4\}$	$O_2CN^iPr_2$	17
**$[Ce_4(\mu_3\text{-}O)_2(O_2CN^iPr_2)_{12}]$**	$\{Ce_4O_2(\mu_3\text{-}O_2CN^iPr_2)_2\}$	$O_2CN^iPr_2$	
**$[Ce_4(\mu_3\text{-}OH)_4(\mu\text{-}tfa)_4(hfa)_4(phen)_4]$**	$\{Ce_4(\mu_3\text{-}OH)_4(\mu\text{-}tfa)_4\}$	hfa^-, phen	18
**$[Ce_4Br_{10}((CH_3)_2CHOH)_{10}(H_2O)_2(OH)_2]$**	$\{Ce_4(\mu_2\text{-}Br)_6(\mu_3\text{-}OH)_2\}$	H_2O, $(CH_3)_2CHOH$, Br	19
**$[Ce_4(\mu_4\text{-}O)(\mu_3\text{-}OPr^i)_2(\mu\text{-}OPr^i)_4(OPr^i)_8]$**	$\{Ce_4(\mu_4\text{-}O)(\mu_3\text{-}OPr^i)_2(\mu\text{-}OPr^i)_4\}$	OPr^i	20
**$[Ce_4^{IV}(TBC[8]\text{-}6H)_2(\mu_3\text{-}O)_2(dmf)_4]\cdot 5DMF\cdot hexane\cdot MeCN$**	$\{Ce_4(\mu_3\text{-}O)_2\}$	TBC[8]-6H, dmf	21
**$[Ce(III)_4(\mu_4\text{-}O)(L\text{–}H)_4(HLH\text{-}H)_3(H\text{-}val)]^{2+}(NO_3^-)_2$**	$\{Ce_4(\mu_4\text{-}O)$		22
**{Ce6}**			
**$[Ce_6O_4(OH)_4(O_2PPh_2)_4(O_2C^tBu)_8]$**	$\{Ce_6^{IV}(\mu_3\text{-}O)_4(\mu_3\text{-}OH)_4\}$	$Ph_2PO_2^-$, $^tBuCO_2^-$	23
**$[Ce_6O_4(OH)_4(O_2C^tBu)_{12}]$**	$\{Ce_6^{IV}(\mu_3\text{-}O)_4(\mu_3\text{-}OH)_4$	$^tBuCO_2^-$	24
**$[Ce_6O_4(OH)_4(BDC)_6]$**	$\{Ce_6O_4(OH)_4\}$	BDC^{2-}	25
**$[Ce_6O_4(OH)_4(FUM)_6]$**	$\{Ce_6O_4(OH)_4\}$	FUM^{2-}	
**$[Ce_6(\mu_3\text{-}OH)_4(\mu_3\text{-}O)_4(BDC)_{12}(H_2O)_6]$**	$\{Ce_6O_4(OH)_4\}$	BDC, H_2O	26
**$[Ce_6(\mu_3\text{-}OH)_4(\mu_3\text{-}O)_4(FBDC)_{12}(H_2O)_6]$**	$\{Ce_6O_4(OH)_4\}$	FBDC, H_2O	
**$[Ce_6(\mu_3\text{-}OH)_4(\mu_3\text{-}O)_4(ABDC)_{12}(H_2O)_6]$**	$\{Ce_6O_4(OH)_4\}$	ABDC, H_2O	
**$[Ce_6(\mu_3\text{-}OH)_4(\mu_3\text{-}O)_4(2,6\text{-}NDC)_{12}(H_2O)_6]$**	$\{Ce_6O_4(OH)_4\}$	2,6-NDC, H_2O	
**$NH_4[Ce_6(\mu_3\text{-}O)_5(\mu_3\text{-}OH)_3(\mu_2\text{-}C_6H_5COO)_9(NO_3)_3(DMF)_3].DMF.H_2O$**	$\{Ce_6O_5(OH)_3(C_6H_5COO)_9\}$	NO_3^{2-}, DMF	27

**[$Ce_6(\mu_3\text{-}O_4)_4(\mu_3\text{-}OH)_4(BPyDC)_6$]**	{$Ce_6(\mu_3\text{-}O_4)_4(\mu_3\text{-}OH)_4$}	$BpyDC^{2-}$	28
**[$Ce_6(\mu_3\text{-}O_4)_4(\mu_3\text{-}OH)_4(TDC)_4(OH)_4(H_2O)_4$]**	{$Ce_6(\mu_3\text{-}O_4)_4(\mu_3\text{-}OH)_4$}	TDC^{2-}, H_2O, OH^-	
**[$Ce_6(\mu_3\text{-}O_4)_4(\mu_3\text{-}OH)_4(PZDC)_4(OH)_4(H_2O)_4$]**	{$Ce_6(\mu_3\text{-}O_4)_4(\mu_3\text{-}OH)_4$}	$PZDC^{2-}$, OH^-, H_2O	
**[$Ce_6(\mu_3\text{-}O_4)_4(\mu_3\text{-}OH)_4(BTC)_4(OH)_6(H_2O)_6$]**	{$Ce_6(\mu_3\text{-}O_4)_4(\mu_3\text{-}OH)_4$}	BTC^{3-}, OH^-, H_2O	
**[$Ce_6O_4(OH)_4(OCOCH_2{}^tBu)_{12}(HOCOCH_2{}^tBu)_4$]**	{$Ce_6O_4(OH)_4$}	$OCOCH_2{}^tBu^-$, $HOCOCH_2{}^tBu$	29
**[$Ce_6(\mu_3\text{-}O_8)(O_2C^tBu)_8(C_4N_3H_{13})_4$]**	{Ce_6O_8}	$O_2C^tBu^-$, $C_4N_3H_{13}$	30
**[$Ce_6O_4(OH)_4(H_2O)_4(dmb)_{12}$]**	{$Ce_6(\mu_3\text{-}O)_4(\mu_3\text{-}OH)_4$}	H_2O, dbm	31
**[$Ce_6(OH)_4O_4(Muc)_6$]**	{$Ce_6(OH)_4O_4$}	Muc^{2-}	32
**[$Ce_6(OH)_4O_4(CDC)_6$]**	{$Ce_6(OH)_4O_4$}	CDC^{2-}	
**[$Ce_6(OH)_4O_4(BA)_6(TCPP\text{-}Zn)_{1.5}$]**	{$Ce_6(OH)_4O_4$}	BA^-, TCPP-Zn	
**[$Ce_6(OH)_4O_4(BA)_4(TCPP\text{-}SnCl_2)_2$]**	{$Ce_6(OH)_4O_4$}	BA^-, TCPP-$SnCl_2$	
**[$Ce_6(OH)_4O_4(BA)_4(TBAPy)_2$]**	{$Ce_6(OH)_4O_4$}	BA^-, TBAPy	
**[$Ce_6(\mu_3\text{-}O)_4(\mu_3\text{-}OH)_4(HCOO)_{10}(NO_3)_2(H_2O)_3$]$\cdot(H_2O)_{9.5}$**	{$Ce_6(\mu_3\text{-}O)_4(\mu_3\text{-}OH)_4$}	NO_3^{2-}, H_2O, $HCOO^-$	33
**[$Ce_6(\mu_3\text{-}O)_4(\mu_3\text{-}OH)_4(HCOO)_{10}(NO_3)_4$]$\cdot(NO_3)_3(NH_4)_5(H_2O)_5$**	{$Ce_6(\mu_3\text{-}O)_4(\mu_3\text{-}OH)_4$}	NO_3^{2-}, H_2O, $HCOO^-$	
**[$Ce_6(pro1)_6(\mu_3\text{-}O)_2(CHO(NMe_2)(OMe)$]$\cdot(H_2O)(CHO(NMe_2))$**	{$Ce_6(\mu_3\text{-}O)_2$}	Pro1	34
**[$Ce_6O_4(OH)_4(FDC)_4(OH)_4(H_2O)_4$]$\cdot$2DMF$\cdot 2H_2O$**	{$Ce_6O_4(OH)_4$}	FDC^{2-}, OH^-, H_2O	35
**[$Ce_6(\mu_3\text{-}O)_4(\mu_3\text{-}OH)_4(SUC)_4(OH)_4(H_2O)_4$]**	{$Ce_6(\mu_3\text{-}O)_4(\mu_3\text{-}OH)_4$}	SUC^{2-}, OH^-, H_2O	36
**[$Ce_6(\mu_3\text{-}O)_4(\mu_3\text{-}OH)_4(SUC)_4(NO_3)_2(OH)_2(H_2O)_2$]**	{$Ce_6(\mu_3\text{-}O)_4(\mu_3\text{-}OH)_4$}	NO_3^{2-}, OH^-, H_2O, SUC^{2-}	
**[$Ce_6(\mu_3\text{-}O)_4(\mu_3\text{-}OH)_4(H_2O)_6(DL\text{-}ASP)_6$]**	{$Ce_6(\mu_3\text{-}O)_4(\mu_3\text{-}OH)_4$}	H_2O, DL-ASP^{2-}	
**[$Ce_6(\mu_3\text{-}O)_4(\mu_3\text{-}OH)_4(H_2O)_6(D\text{-}ASP)_6$]**	{$Ce_6(\mu_3\text{-}O)_4(\mu_3\text{-}OH)_4$}	H_2O, D-ASP^{2-}	
**[$Ce_6(\mu_3\text{-}O)_4(\mu_3\text{-}OH)_4(H_2O)_6(L\text{-}ASP)_6$]**	{$Ce_6(\mu_3\text{-}O)_4(\mu_3\text{-}OH)_4$}	H_2O, L-ASP^{2-}	
**[$Ce_6O_8(O_2C^tBu)_4(L^1)_4$]**	{$Ce_6^{IV}O_8$}	O_2C^tBu, L^1	37
**[$Ce_6O_8(O_2CC_6H_5)_8(L^1)_4$]**	{$Ce_6^{IV}O_8$}	$O_2CC_6H_5$, L^1	
**[$Ce_6O_8(O_2CC_6H_4\text{-}4\text{-}OCH_3)_8(L^1)_4$]**	{$Ce_6^{IV}O_8$}	$O_2CC_6H_4$-4-OCH_3, L^1	
**[$Ce_6O_4(OH)_4(O_2C^tBu)_{12}(L^1)_2$]**	{$Ce_6^{IV}O_4(OH)_4$}	O_2C^tBu, L^1	
**[$Ce_6O_4(OH)_4(PDC)_6$]**	{$Ce_6O_4(OH)_4$}	PDC	38
**[$Ce_6^{IV}(TBC[8]\text{-}6H)_2(\mu_3\text{-}O)_2(\mu_2OMe)_4(\mu_2\text{-}O)_2(dmf)_4$]$\cdot$6DMF$\cdot$hexane**	{$Ce_6(\mu_3\text{-}O)_2(\mu_2OMe)_4(\mu_2\text{-}O)_2$}	TBC[8]-6H, dmf	21

$[Ce_6O_4(OH)_3F(O_2CPh)_{12}py_2]$	$\{Ce_6O_4(OH)_3F\}$	O_2CPh, py	39
$[C_{36}H_{56}Ce_6F_{36}O_{50}S_{12}]\cdot 2(C_5H_9N_2)\cdot 2(C_4H_8O)$			40
$[Ce_6(\mu_3\text{-}O)_4(\mu_3\text{-}OH)_4(NH_3CH_2COO)_8(NO_3)_4(H_2O)_6]Cl_8\cdot 8H_2O$	$\{Ce_6O_4(OH)_4\}$	NH_3CH_2COO, NO_3^{2-}, H_2O	41
$[Ce_6(\mu_3\text{-}O)_4(\mu_3\text{-}OH)_4(NH_3CH_2COO)_9(NO_3)_5H_2O](NO_3)7\cdot 6\text{–}7H_2O$	$\{Ce_6O_4(OH)_4\}$	NH_3CH_2COO, NO_3^{2-}, H_2O	
$[Ce_6(\mu_3\text{-}O)_4(\mu_3\text{-}OH)_4(NH_3CH_2COO)_9(NO_3)_6](NO_3)_6\cdot 24H_2O$	$\{Ce_6O_4(OH)_4\}$	NH_3CH_2COO, NO_3^{2-}	
$\{Ce_{>7}\}$			
$[(THF)_8Ce_8O_2S_2(SePh)_{16}]$	$\{Ce_8O_2S_2(SePh)_{14}\}$	THF, $SePh^-$	42
$(THF)_8Ce_8(\mu_3\text{-}O)_2(\mu_5\text{-}Se)_2(SePh)_{16}\cdot 6THF$	$\{Ce_8(\mu_3\text{-}O)_2(\mu_5\text{-}Se)_2\}$	SePh, THF	43
$(pyH)_8[Ce_{10}O_4(OH)_4(O_3PPh)_{12}(NO_3)_{12}]$	$\{Ce_6^{IV}(\mu_3\text{-}O)_4(\mu_3\text{-}OH)_4\}\{Ce^{IV}(\mu_3\text{-}PhPO_3)\}_4$	$PhPO_3^{2-}$, NO_3^{2-}	44
$[Ce_{13}O_8((OCH_2CH_2)_2N(C_6H_5))_{18}]$	$\{Ce_{13}O_8\}$	$(OCH_2CH_2)_2N(C_6H_5)$	45
$[Ce_{22}O_{20}(OH)_4(ib)_{26}(tme)_4]$	$\{Ce_{22}O_{24}\}$	Ib^-, tme^{3-}	46
$[Ce_{24}O_{28}(OH)_8(PhCO_2)_{30}(py)_4]$	$\{Ce_{24}O_{36}\}$	py, $PhCO_2^-$	47
$[Ce_{38}O_{54}(OH)_8(EtCO_2)_{36}(py)_8]$	$\{Ce_{38}O_{62}\}$	$EtCO_2^-$, py	47
$K_{10}[Ce^{IV}_{38-n}Ce^{III}_nO_{56-(n+1)}(OH)_{n+1}Cl_{51}(H_2O)_{11}]\cdot xH_2O$	$\{Ce_{38}O_{56-(n+1)}(OH)_{n+1}\}$	Cl^-, H_2O	8
$[Ce(IV)_{38}(C_6H_5COO)_{16}(\mu_4\text{-}O)_{34}(\mu_4\text{-}OH)_2(\mu_3\text{-}O)_{24}(\mu_2\text{-}OH)_8(OH)_{10}(H_2O)_4](H_2O)$	$\{Ce_{38}O_{58}(OH)_{20}\}$	$C_6H_5COO^-$, H_2O	48
$[Ce_{38}O_{54}(OH)_8(CH_3CH_2CO_2)_{36}(C_5H_5N)_8]$	$\{Ce_{38}O_{54}(OH)_8\}$	$CH_3CH_2CO_2$, C_5H_5N	
$[Ce_{40}O_{56}(OH)_2(MeCO_2)_{44}(MeCO_2H)_2(py)_4]$/$[Ce_{40}O_{56}(OH)_2(MeCO_2)_{44}(MeCN)_2(py)_4]$	$\{Ce_{40}O_{58}\}$	$MeCO_2^-$, py, $MeCO_2H$/ $MeCN_2^-$, MeCN, py	47
$[Ce_{100}O_{149}(OH)_{18}(O_2CPh)_{60}(PhCO_2H)_{12}(H_2O)_{20}]$	$\{Ce_{100}O_{167}\}$	O_2CPh, $PhCO_2H$, H_2O	44

XV. REFERENCES

1. Nakamoto, K., Applications in Coordination Chemistry. In *Infrared and Raman Spectra of Inorganic and Coordination Compounds*, Wiley: 2008; pp 1-273.
2. acetylacetone IR spectrum In *SDBSWeb*, National Institute of Advanced Industrial Science and Technology.
3. Nakamoto, K.; McCarthy, P. J.; Martell, A. E., Infrared Spectra of Metal Chelate Compounds. III. Infrared Spectra of Acetylacetonates of Divalent Metals[1]. *J. Am. Chem. Soc.* **1961,** *83* (6), 1272-1276.
4. Kakihana, M.; Kotaka, M.; Okamoto, M., Vibrational analysis of acetate ion molecules and estimation of equilibrium constants for their hydrogen isotopic exchange reactions. *J. Phys. Chem.* **1983,** *87* (14), 2526-2535.
5. Ostrowska, K.; Kaźmierska, A.; Rąpała-Kozik, M.; Kalinowska-Tłuścik, J., Ratiometric fluorescent $Zn^{2+}$and In^{3+}receptors of fused pyrazine with an aminopropanol chain in acetonitrile. *New J. Chem.* **2014,** *38* (1), 213-226.
6. Solomon, E. I.; Hedman, B.; Hodgson, K. O.; Dey, A.; Szilagyi, R. K., Ligand K-edge X-ray absorption spectroscopy: covalency of ligand–metal bonds. *Coord. Chem. Rev.* **2005,** *249* (1), 97-129.
7. George, G. N., EDG_FIT. Stanford Synchrotron Radiation Source.
8. Wacker, J. N.; Ditter, A. S.; Cary, S. K.; Murray, A. V.; Bertke, J. A.; Seidler, G. T.; Kozimor, S. A.; Knope, K. E., Reactivity of a Chloride Decorated, Mixed Valent $Ce^{III/IV38}$–Oxo Cluster. *Inorg. Chem.* **2022,** *61* (1), 193-205.
9. Boyle, T. J.; Tribby, L. J.; Bunge, S. D., Synthesis and Structural Characterization of a Series of Carboxylic Acid Modified Cerium(III) Alkoxides. *EurJIC* **2006,** *2006* (22), 4553-4563.
10. Lehn, J.-S. M.; Hoffman, D. M., *CSD Communication (Private Communication)* **2005**.
11. Bilyk, A.; Dunlop, J. W.; Fuller, R. O.; Hall, A. K.; Harrowfield, J. M.; Hosseini, M. W.; Koutsantonis, G. A.; Murray, I. W.; Skelton, B. W.; Sobolev, A. N.; Stamps, R. L.; White, A. H., Systematic Structural Coordination Chemistry of p-tert-Butyltetrathiacalix[4]arene: Further Complexes of Lanthanide Metal Ions. *EurJIC* **2010,** *2010* (14), 2127-2152.
12. Aspinall, H. C.; Bacsa, J.; Jones, A. C.; Wrench, J. S.; Black, K.; Chalker, P. R.; King, P. J.; Marshall, P.; Werner, M.; Davies, H. O.; Odedra, R., Ce(IV) Complexes with Donor-Functionalized Alkoxide Ligands: Improved Precursors for Chemical Vapor Deposition of CeO2. *Inorg. Chem.* **2011,** *50* (22), 11644-11652.
13. Schläfer, J.; Stucky, S.; Tyrra, W.; Mathur, S., Heterobi- and Trimetallic Cerium(IV) tert-Butoxides with Mono-, Di-, and Trivalent Metals (M = K(I), Ge(II), Sn(II), Pb(II), Al(III), Fe(III)). *Inorg. Chem.* **2013,** *52* (7), 4002-4010.
14. Friedrich, J.; Schneider, D.; Bock, L.; Maichle-Mössmer, C.; Anwander, R., Cerium(IV) Neopentoxide Complexes. *Inorg. Chem.* **2017,** *56* (14), 8114-8127.
15. Arnold, P. L.; Casely, I. J.; Zlatogorsky, S.; Wilson, C., Organometallic Cerium Complexes from Tetravalent Coordination Complexes. *Helv. Chim. Acta.* **2009,** *92* (11), 2291-2303.
16. Bock, L.; Tran, X.; Liang, Y.; Kramer, M.; Maichle-Mössmer, C.; Anwander, R., SOMC@Periodic Mesoporous Silica Nanoparticles: Meerwein–Ponndorf–Verley Reduction Promoted by Immobilized Rare-Earth-Metal Alkoxides. *Organometallics* **2020,** *39* (7), 1046-1058.
17. Baisch, U.; Dell' Amico, D. B.; Calderazzo, F.; Labella, L.; Marchetti, F.; Vitali, D., Reaction of a tetranuclear N,N-di-iso-propylcarbamato complex of cerium(III) with dioxygen: synthesis and X-ray characterization of both the oxidation product and its precursor. *J. Mol. Catal. A Chem.* **2003,** *204-205*, 259-265.
18. Wong, H.-Y.; Chan, W. T. K.; Law, G.-L., Assembly of Lanthanide(III) Cubanes and Dimers with Single-Molecule Magnetism and Photoluminescence. *Inorg. Chem.* **2018,** *57* (12), 6893-6902.

19. Vaughn, S. A.; Severance, R. C.; Smith, M. D.; zur Loye, H.-C., Crystal Growth, Structural Motifs, and Optical Properties of Molecular and Polymeric Cerium Halide Materials. *Cryst. Growth Des.* **2011,** *11* (11), 5072-5078.
20. Sirio, C.; Hubert-Pfalzgraf, L. G.; Bois, C., Facile thermal desolvation of $Ce_2(OPr^i)_8(Pr^iOH)_2$: Characterization and molecular structure of $Ce_4(\mu_4\text{-}O)(\mu_3\text{-}OPr^i)(\mu\text{-}OPr^i)_4(OPr^i)_8$. *Polyhedron* **1997,** *16* (7), 1129-1136.
21. Taylor, S. M.; Sanz, S.; McIntosh, R. D.; Beavers, C. M.; Teat, S. J.; Brechin, E. K.; Dalgarno, S. J., p-tert-Butylcalix[8]arene: An Extremely Versatile Platform for Cluster Formation. *Chem. Eur. J.* **2012,** *18* (50), 16014-16022.
22. Arumugam, S.; Shankar, B.; Mondal, K. C., Redox Active Hexanuclear Mixed Valence Dicationic Ce(III)/Ce(IV) Coordination Clusters. *EurJIC* **2020,** *2020* (43), 4127-4136.
23. Russel-Webster, B.; Lopez-Nieto, J.; Abboud, K. A.; Christou, G., *Dalton Trans.* **2021,** *50*, 15524.
24. Mereacre, V.; Ako, A. M.; Akhtar, M. N.; Lindemann, A.; Anson, C. E.; Powell, A. K., Homo- and Heterovalent Polynuclear Cerium and Cerium/Manganese Aggregates. *Helv. Chim. Acta.* **2009,** *92* (11), 2507-2524.
25. Lammert, M.; Wharmby, M. T.; Smolders, S.; Bueken, B.; Lieb, A.; Lomachenko, K. A.; Vos, D. D.; Stock, N., Cerium-based metal organic frameworks with UiO-66 architecture: synthesis, properties and redox catalytic activity. *ChemComm* **2015,** *51* (63), 12578-12581.
26. Zhang, Y.-F.; Wang, Q.; Xue, D.-X.; Bai, J., Single-Crystal Synthesis and Diverse Topologies of Hexanuclear Ce^{IV}-Based Metal–Organic Frameworks. *Inorg. Chem.* **2020,** *59* (16), 11233-11237.
27. Das, R.; Sarma, R.; Baruah, J. B., A hexanuclear cerium(IV) cluster with mixed coordination environment. *Inorg. Chem. Commun.* **2010,** *13* (6), 793-795.
28. Lammert, M.; Glißmann, C.; Reinsch, H.; Stock, N., Synthesis and Characterization of New Ce(IV)-MOFs Exhibiting Various Framework Topologies. *Cryst. Growth Des.* **2017,** *17* (3), 1125-1131.
29. Shirase, S.; Tamaki, S.; Shinohara, K.; Hirosawa, K.; Tsurugi, H.; Satoh, T.; Mashima, K., Cerium(IV) Carboxylate Photocatalyst for Catalytic Radical Formation from Carboxylic Acids: Decarboxylative Oxygenation of Aliphatic Carboxylic Acids and Lactonization of Aromatic Carboxylic Acids. *J. Am. Chem. Soc.* **2020,** *142* (12), 5668-5675.
30. Grebenyuk, D.; Martynova, I.; Tsymbarenko, D., Self-Assembly of Hexanuclear Lanthanide Carboxylate Clusters of Three Architectures. *EurJIC* **2019,** *2019* (26), 3103-3111.
31. Mitchell, K. J.; Goodsell, J. L.; Russell-Webster, B.; Twahir, U. T.; Angerhofer, A.; Abboud, K. A.; Christou, G., Expansion of the Family of Molecular Nanoparticles of Cerium Dioxide and Their Catalytic Scavenging of Hydroxyl Radicals. *Inorg. Chem.* **2021,** *60* (3), 1641-1653.
32. Smolders, S.; Struyf, A.; Reinsch, H.; Bueken, B.; Rhauderwiek, T.; Mintrop, L.; Kurz, P.; Stock, N.; De Vos, D. E., A precursor method for the synthesis of new Ce(IV) MOFs with reactive tetracarboxylate linkers. *ChemComm* **2018,** *54* (8), 876-879.
33. Hennig, C.; Ikeda-Ohno, A.; Kraus, W.; Weiss, S.; Pattison, P.; Emerich, H.; Abdala, P. M.; Scheinost, A. C., Crystal Structure and Solution Species of Ce(III) and Ce(IV) Formates: From Mononuclear to Hexanuclear Complexes. *Inorg. Chem.* **2013,** *52* (20), 11734-11743.
34. Zhu, W.; Wu, X.; He, C.; Duan, C., Proline adducts of metallo-organic, sextuply-stranded lanterns as homogeneous catalysts for asymmetric catalysis. *Tetrahedron* **2013,** *69* (48), 10477-10481.
35. Dreischarf, A. C.; Lammert, M.; Stock, N.; Reinsch, H., Green Synthesis of Zr-CAU-28: Structure and Properties of the First Zr-MOF Based on 2,5-Furandicarboxylic Acid. *Inorg. Chem.* **2017,** *56* (4), 2270-2277.
36. Jacobsen, J.; Achenbach, B.; Reinsch, H.; Smolders, S.; Lange, F.-D.; Friedrichs, G.; De Vos, D.; Stock, N., The first water-based synthesis of Ce(IV)-MOFs with saturated chiral and achiral C_4-dicarboxylate linkers. *Dalton Trans* **2019,** *48* (23), 8433-8441.

37. Mathey, L.; Paul, M.; Copéret, C.; Tsurugi, H.; Mashima, K., Cerium(IV) Hexanuclear Clusters from Cerium(III) Precursors: Molecular Models for Oxidative Growth of Ceria Nanoparticles. *Chemistry – A European Journal* **2015,** *21* (38), 13454-13461.
38. Waitschat, S.; Fröhlich, D.; Reinsch, H.; Terraschke, H.; Lomachenko, K. A.; Lamberti, C.; Kummer, H.; Helling, T.; Baumgartner, M.; Henninger, S.; Stock, N., Synthesis of M-UiO-66 (M = Zr, Ce or Hf) employing 2,5-pyridinedicarboxylic acid as a linker: defect chemistry, framework hydrophilisation and sorption properties. *Dalton Trans.* **2018,** *47* (4), 1062-1070.
39. Russell-Webster, B.; Abboud, K. A.; Christou, G., Molecular nanoparticles of cerium dioxide: structure-directing effect of halide ions. *ChemComm* **2020,** *56* (40), 5382-5385.
40. Blasberg, F.; Bolte, M.; Wagner, M., *CSD Communication (Private Communication)* **2022**.
41. Estes, S. L.; Antonio, M. R.; Soderholm, L., Tetravalent Ce in the Nitrate-Decorated Hexanuclear Cluster $[Ce_6(\mu_3\text{-}O)_4(\mu_3\text{-}OH)_4]^{12+}$: A Structural End Point for Ceria Nanoparticles. *J. Phys. Chem. C.* **2016,** *120* (10), 5810-5818.
42. Norton, K.; Banerjee, S.; Das, S.; Huebner, L.; Emge, T. J.; Brennan, J. G., Lanthanide oxochalcogenido clusters. *Dalton Trans.* **2010,** *39* (29), 6794-6800.
43. Banerjee, S.; Huebner, L.; Romanelli, M. D.; Kumar, G. A.; Riman, R. E.; Emge, T. J.; Brennan, J. G., Oxoselenido Clusters of the Lanthanides: Rational Introduction of Oxo Ligands and Near-IR Emission from Nd(III). *J. Am. Chem. Soc.* **2005,** *127* (45), 15900-15906.
44. Russell-Webster, B.; Lopez-Nieto, J.; Abboud, K. A.; Christou, G., Phosphorus-based ligand effects on the structure and radical scavenging ability of molecular nanoparticles of CeO2. *Dalton Trans.* **2021,** *50* (43), 15524-15532.
45. Yuan, F.; Gu, Z.; Li, L.; Sha, L., Novel cerium(IV)-diolate complex with a 13-nuclear cerium(IV)-oxo core: Synthesis, molecular structure and catalytic property for ε-caprolactone-polymerization. *Polyhedron* **2017,** *133*, 393-397.
46. Malaestean, I. L.; Ellern, A.; Baca, S.; Kogerler, P., Cerium oxide nanoclusters: commensurate with concepts of polyoxometalate chemistry? *Chem. Commun.* **2012,** *48* (10), 1499-501.
47. Mitchell, K. J.; Abboud, K. A.; Christou, G., Atomically-precise colloidal nanoparticles of cerium dioxide. *Nat. Commun.* **2017,** *8* (1), 1445.
48. Wasson, M. C.; Zhang, X.; Otake, K.-i.; Rosen, A. S.; Alayoglu, S.; Krzyaniak, M. D.; Chen, Z.; Redfern, L. R.; Robison, L.; Son, F. A.; Chen, Y.; Islamoglu, T.; Notestein, J. M.; Snurr, R. Q.; Wasielewski, M. R.; Farha, O. K., Supramolecular Porous Assemblies of Atomically Precise Catalytically Active Cerium-Based Clusters. *Chem. Mater.* **2020,** *32* (19), 8522-8529.
